# Association between *ELMO1* gene polymorphisms and diabetic kidney disease: A systematic review and meta-analysis

**DOI:** 10.1371/journal.pone.0295607

**Published:** 2024-01-26

**Authors:** Alireza Azarboo, Shaghayegh Hosseinkhani, Amirhossein Ghaseminejad-Raeini, Hossein Aazami, Sayed Mohammad Mohammadi, Saba Zeidi, Farideh Razi, Fatemeh Bandarian

**Affiliations:** 1 Endocrinology and Metabolism Research Center, Endocrinology and Metabolism Clinical Sciences Institute, Tehran University of Medical Sciences, Tehran, Iran; 2 School of Medicine, Tehran University of Medical Sciences, Tehran, Iran; 3 Metabolic Disorders Research Center, Endocrinology and Metabolism Molecular-Cellular Sciences Institute, Tehran University of Medical Sciences, Tehran, Iran; 4 Evidence Based Medicine Research Center, Endocrinology and Metabolism Clinical Sciences Institute, Tehran University of Medical Sciences, Tehran, Iran; 5 Cell Therapy and Regenerative Medicine Research Center, Endocrinology and Metabolism Molecular-Cellular Sciences Institute, Tehran University of Medical Sciences, Tehran, Iran; 6 Diabetes Research Center, Endocrinology and Metabolism Clinical Sciences Institute, Tehran University of Medical Sciences, Tehran, Iran; 7 Metabolomics and Genomics Research Center, Endocrinology and Metabolism Molecular-Cellular Sciences Institute, Tehran University of Medical Sciences, Tehran, Iran; Neyshabur University of Medical Sciences, ISLAMIC REPUBLIC OF IRAN

## Abstract

**Background:**

Previous research has suggested that the *ELMO1* gene may play a role in the development of diabetic kidney disease. Diabetic kidney disease (DKD) is a serious complication of diabetes and the leading cause of chronic kidney disease and end-stage renal disease (ESRD).

**Objective and rationale:**

This study aim was to systematically review and explore the association between *ELMO1* gene polymorphisms and diabetic kidney disease. A comprehensive systematic review provides a clear conclusion and high-level evidence for the association between ELMO1 gene and DKD for future application in personalized medicine.

**Methods:**

A comprehensive search of electronic databases, per PRISMA instructions, was conducted in Scopus, EMBASE, Web of Science, and PubMed databases from 1980 to January 2023. Pooled odds ratios (ORs) and 95% confidence intervals (CIs) were calculated using appropriate models. Subgroup and sensitivity analyses were performed to explore potential sources of heterogeneity and assess the robustness of the findings.

**Results:**

A total of 5794 diabetes patients with DKD, 4886 diabetes patients without DKD, and 2023 healthy controls were included in the 17 studies that made up this systematic review. In the investigation of DM (Diabetes Mellitus) with DKD vs. DM without DKD, the susceptibility for DKD for the EMLO1 rs741301 polymorphism indicated a significant difference under the dominant, homozygote, and recessive genetic models. The susceptibility for DKD for the EMLO1 rs1345365, rs10255208, and rs7782979 polymorphisms demonstrated a significant difference under the allele genetic models in the analysis of DM with DKD vs. DM without DKD groups. There was a considerable increase in DKD risk in the Middle East when the population was stratified by the region.

**Conclusion:**

The findings of the meta-analysis show that there are a significant connection between the *EMLO1* rs741301 polymorphism and DKD susceptibility in overall analyses; as well as rs1345365, rs10255208, and rs7782979 polymorphisms; especially in the Middle East region.

## Introduction

Over the past few decades, type 2 diabetes mellitus (T2DM), which is characterized by chronic blood glucose elevation brought on by peripheral insulin resistance and harmful effects on both micro- and macro-circulation [1], has emerged as a silent pandemic, causing a notable loss in the government’s economy and endangering human health [2]. There were 366 million T2DM cases worldwide in 2011, and by 2030, that number is expected to increase to 552 million [3]. However, T2DM can also cause high rates of complications, morbidity, and mortality [4]. Diabetic kidney disease (DKD) is a significant condition that affects 40% of type 2 diabetics. End-stage renal failure (ESRD) is brought on by the major microvascular consequence of diabetes mellitus (DM) known as DKD, which has significant mortality and disability rates [5]. Although the precise pathophysiology of DKD is still unknown, it is clear that environmental variables, in conjunction with genetic factors, play a critical role in the onset and progression of DKD [6].

Numerous genetic single nucleotide polymorphisms (SNPs), which are implicated in the pathophysiology of DKD, have been found to be related with DKD susceptibility in recent genome-wide association studies (GWAS) [7]. By interacting with the cytokinesis protein during cell motility and apoptotic cell phagocytosis, *ELMO1*, a soluble cytoplasmic protein of 720 amino acids, has been identified as a crucial mediator in the pathophysiology of cytoskeletal rearrangements [8]. It has been shown that excessive glucose enhances the expression of *ELMO1*, which inhibits cell adhesion and encourages the development of transcription growth factor (TGF), collagen type 1, fibronectin, and integrin-linked kinase to mediate the pathogenesis of DKD [9, 10]. The *ELMO1* gene, which is located on chromosome 7p14, was identified as a new candidate gene for DKD in the first GWAS conducted on Japanese individuals [11]. A recent meta-analysis on the genetic map of diabetic nephropathy reported *ELMO1* rs741301 to significantly increase the risk of diabetic nephropathy (DN) in diabetics with normoalbuminuria [12].

However, due to the sample size, false positive results, and various ethnicities within other studies, the results on *ELMO1* gene polymorphism were disputed and inconsistent. In order to more accurately analyze *ELMO1* polymorphism’s connection with DKD, we conducted this meta-analysis to evaluate the pooled effect of the polymorphism and addressed significant heterogeneity using established methods.

## Materials and methods

This systematic review was conducted according to PRISMA instructions (Preferred Reporting Items for Systematic Reviews and Meta-Analyses) [13]. The study protocol was registered and approved by PROSPERO 2022 with number CRD42022307667. Also, the study protocol was approved by the Ethics Committee at the Endocrinology and Metabolism Research Institute, Tehran University of Medical Sciences, Tehran, Iran, with number IR.TUMS.EMRI.REC.1401.020.

### Literature search strategy

A comprehensive search was performed in databases including Scopus, EMBASE, Web of Science, and PubMed from 1980 to January 2023 using the search strategy. The search terms were "gene", "genotype", "genetic", "polymorphism", "SNP", "variant", ""engulfment and cell motility 1", "*ELMO1*", "nephropathy", "diabet* nephro", "diabetes mellitus", or their equivalents and their combinations by application of operators "OR" and "AND". Search results were sent to the endnote software, and duplicate documents were removed. After that, in the next step, the remaining records were evaluated by title and abstract. After removing unrelated records, the full texts of the remaining documents were evaluated, and their references were searched manually. In cases of unavailable or incomplete data, the record was excluded.

### Inclusion and exclusion criteria

Inclusion criteria was: investigation of the association between *ELMO1* gene polymorphisms and DKD, including patients with type 1 or type 2 diabetes, reporting the genotype or allele frequencies of *ELMO1* gene polymorphisms in cases and controls, providing sufficient data to calculate odds ratios (ORs) and their corresponding 95% confidence intervals (CIs) and English language. Exclusion criteria was: insufficient data for genotyping distribution, unavailable data, experimental and animal studies, clinical trials, case reports, letters to editors, theses, and review articles. Screening and evaluation of studies were performed by two independent reviewers, and any discrepancy was resolved by discussion or by the third expert researcher.

### Data extraction and quality assessment

Data was extracted from eligible papers. Extracted data included the name of the author, publication year, study design, country, participants’ characteristics (sample size, age, sex), studied gene (variant, SNPs), allele frequency, and Genotyping method “S1 Table in S1 File”. The quality of each study was evaluated using the Newcastle-Ottawa Scale (NOS) [14]. Three factors of quality are included in the NOS checklist: (1) the population that was chosen; (2) the comparability of the groups; and (3) the evaluation of the exposure or outcome of interest for case-control or cohort studies. Each study received a grade ranging from 0 to 9. High-quality studies were those whose scores were greater than or equal to 7 [14]. Due to the low heterogeneity of genes and SNPs studied and analysed, meta-analysis was possible and performed.

### Statistical analysis

For this meta-analysis, STATA version 17.0 software (Stata Corporation, College Station, Texas, USA) was used. Using a Chi-square-based Q test and I2 statistics, heterogeneity among the included studies was evaluated [15]. Results with p < 0.10 or I2 > 50% were deemed to show significant heterogeneity, and the random-effects model was applied [16, 17]. Otherwise, the fixed-effect model (the Mantel-Haenszel method) was applied for analysis (p ≥ 0.1) [18, 19].

Sensitivity analysis was carried out by successively excluding one study at a time in order to gauge the effect of each study on the current meta-analysis. To assess the relationships between EMLO1 polymorphisms and susceptibility to DKD under five genetic models, the combined odds ratio (OR) and its associated 95% confidence interval (CI) were computed: recessive model (Minor homozygote vs. Heterozygote + Major homozygote); dominant model (Minor homozygote + Heterozygote vs. Major homozygote); codominant model (Heterozygote vs. Major homozygote); homozygote model (Minor homozygote vs. Major homozygote); allele model (Minor allele number vs. Major allele number). Publication bias was evaluated by Egger’s regression intercept test (p < 0.05 was considered significant) [20, 21], as well as visually by looking at the symmetry of funnel plots. The trim and fill method was employed to assess the influence on the outcome for any potential publication bias [22, 23]. Subgroup analyses based on region was used to pinpoint the potential causes of heterogeneity. Galbraith plot was also used to explore sources of heterogeneity when subgroup analyses could not find the heterogeneity source [24].

## Results and discussion

### Study characteristics & demography

**Fig 1:**
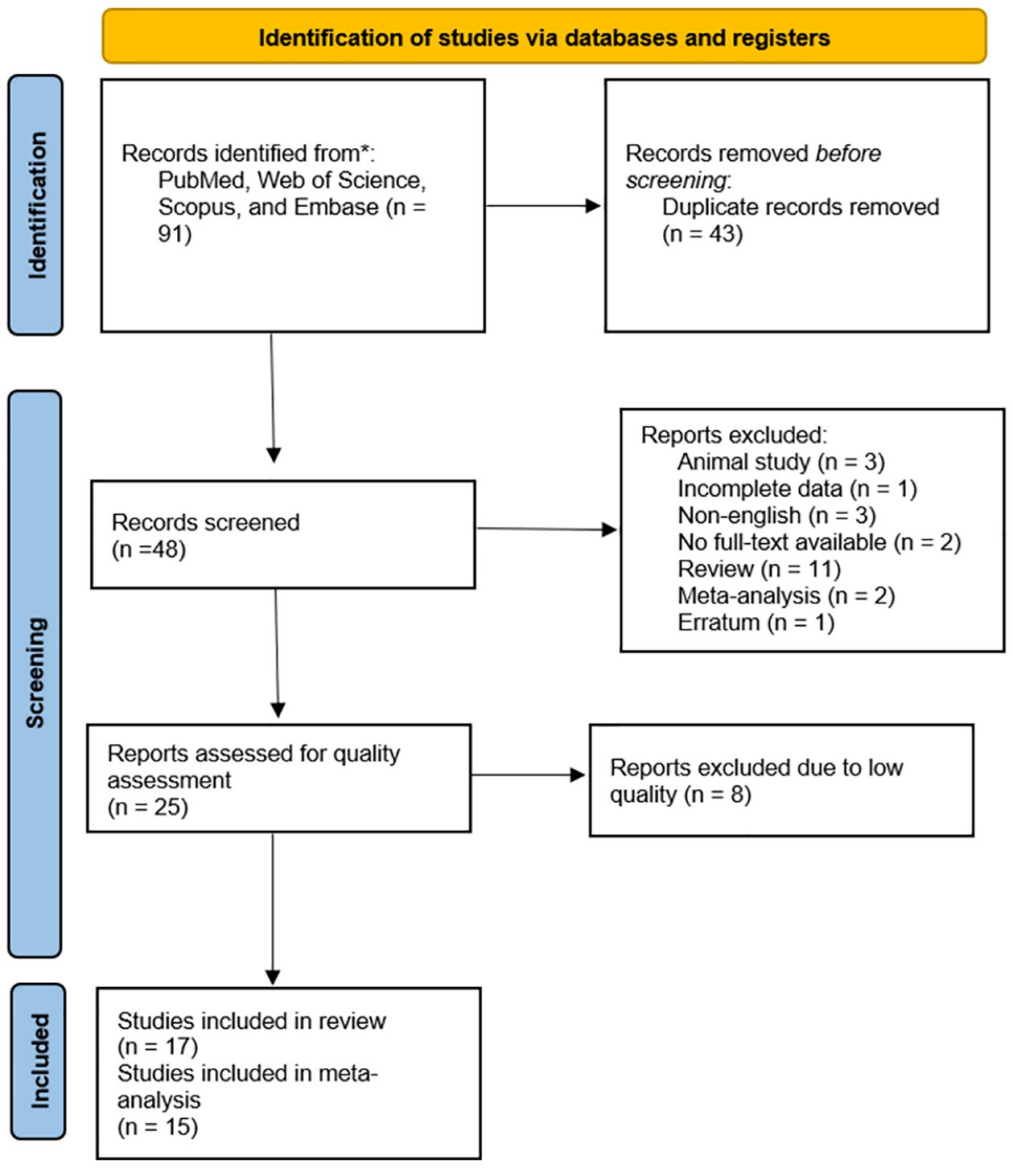
PRISMA flowchart of study selection process.

This systematic review finally included 17 studies, of which 15 studies were eligible and included in meta-analysis and the other two studies were excluded due to lack of data (Fig 1). Twelve studies followed a case-control design [4, 11, 25–34] while the other 5 were genome-wide association studies [7, 35–38], with a total of 5794 DM with DKD patients, 4886 DM without DKD patients, and 2023 healthy controls to assess the impact of *ELMO1* polymorphism on DKD susceptibility. Most studies included patients with T2DM, except for two studies with T1DM patients [7, 36]. India [5, 8, 10, 15, 26, 31, 33, 35] and China [2, 4, 5, 9, 14, 30, 33, 38] were countries with the most studies.

Studies were sub-grouped based on their geographical region; Middle East [25, 28, 29, 32, 37], South Asia [26, 31, 33, 35], East Asia [4, 11, 30, 33, 38], Europe [34], and USA [7, 27, 36]. Although not mentioned in a few studies, our analysis involved 5711 males and 5581 females with the mean age and BMI of 60.4±7.7 years and 27.4±3.2 kg/m2. MassArray was the most Genotyping method (in eight studies) used in the included studies followed by RT-PCR (three studies) and both RFLP and Tetra-Arms (each in two studies). The most common EMO1 variant investigated in these studies was rs741301 (in 13 studies), rs1345365 (in six studies) and rs10951509 (in three studies) “Table 1”.

**Table 1:** The main characteristics of included studies.

Study	Groups	Country	study design	type of diabetes	Sample size	male/female	Age (mean±SD)	SNPs	Genotyping methods	NOS
**Bayoumy2020 [25]**	DKD	Egypt	case-control	T2DM	200	122/78	54±6.1	rs741301	RT-PCR and the allele discrimination technique	9
	DM				200	130/122	52.6±6.2			
	Control				100	68/32	50.2±4.8			
**Shimazaki(1)2005 [11]**	DKD	Japan	case-control	T2DM—DKD	94	63/31	57.9±12.5	rs741301	RT-PCR.—ISH.	9
	DM				94	37/57	62.7±9.9			
**Shimazaki(2)2005 [11]**	DKD	Japan	case-control	T2DM—DKD	466	305/161	59.6±13.5			
	DM				266	125/141	62.9±12			
**Craig2009 [36]**	DKD	USA	genome-wide association study	T1DM	462	236/226	43.6±6	rs6462776, rs6462777	PicoGreen method—iPLEX assay in conjunction with the MassARRAY platform	8
	DM				470	232/238	42.8±6.5			
**Leak2009 [27]**	DKD	USA	case-control	T2DM	1135	439/696	61.6±10	rs9969311, rs2717972, rs1345365, rs2058730, rs10951509	Single-SNP genotypic association	9
	Control				1160	526/634	50.8±11			
**Pezzolesi2009 [7]**	DKD	USA	genome-wide association study	T1DM—DKD	820	423/397	43.11±6.9	rs11769038, rs1882080, rs10255208, rs7782979, rs7799004	Illumina Genotyping Services—MassARRAY genotyping system	8
	DM				885	363/522	38.3±8.7			
**Hanson2010 [26]**	DKD	India	case-control	T2DM—DKD	141	53/88	51±11.5	rs1541727, rs4723596, rs11983698, rs7782590, rs1981740, rs6462733,	AD-PCR—iPLEX assay	7
	DM				416	146/270	42.2±11.9			
**Wu2013 [30]**	DKD	China	case-control	T2DM—DKD	123	67/56	63.28±7.81	rs1345365, rs10951509, rs1981740, rs2058730, rs11769038, rs741301	SEQUENOM MassARRAY system	9
	Control				77	37/40	58.72±13.13			
**Yadav2014 [31]**	DKD	India	case-control	T2DM—DKD	202	140/62	56.7±8.8	rs741301	(PCR),(RFLP),Taqman allele discrimination assay	7
	DM				215	143/72	55.7±10			
	Control				197	122/75	53.9±11.3			
**Bodhini2016 [35]**	DKD	India	genome-wide association	T2DM—DKD	583	376/207	62±9	rs741301	MassARRAY system	9
	DM				601	370/231	64±8			
**Mehrabzadeh2016 [28]**	DKD	Iran	case-control	T2DM—DKD	100	50/50	61.5±8.5	rs741301, rs1345365	tetra-ARMS-PCR	9
	DM				100	50/50	57.36±8.1			
	Control				100	50/50	50.5±10.7			
**Hou2019 [38]**	DKD	China	genome-wide association study	T2DM—DKD	660	378/282	65.8±13.8	rs741301, rs10255208, rs1345365, rs7782979	(PCR), (RFLP),	9
	DM				665	389/276	66.3±14.3			
**Mohammed2019 [32]**	DKD	Iraq	case-control	T2DM—DKD	36	NA	NA	rs741301	Tetra ARMS-PCR	7
	DM				36	NA	NA			
	Control				37	NA	NA			
**Yahya(1)2019 [33]**	DKD	Maleysia	case-control	T2DM—DKD	131	NA	59.0±8.23	rs1799987, rs3917887, rs4073, rs741301	Sequenom Mass ARRAY iPLEX	7
	DM				227	NA				
**Yahya(2)2019 [33]**	DKD	China	case-control	T2DM—DKD	108	NA	63.28±11.56			
	DM				95	NA				
**Yahya(3)2019 [33]**	DKD	India	case-control	T2DM—DKD	86	NA	61.33±10.1			
	DM				136	NA				
**Kwiendacz2020 [34]**	DKD	Poland	case-control	T2DM—DKD	117	202M/170F	63.7±8	rs741301	(PCR)	9
	DM				155					
**Yang2020 [4]**	DKD	China	case-control	T2DM—DKD	208	129/79	58.9±10.5	rs10951509, rs1345365, rs741301	PCR-MassARRAY method using an iPLEX Gold Reagent Kit	8
	DM				200	110/90	60.6±9.4			
	Control				206	NA	NA			
**Omar2021 [29]**	DKD	Egypt	case control	T2DM—DKD	100	64/36	48.78±5	rs741301	real-time PCR sys- tem (Rotor-Gene, Applied Biosystems, Foster City, USA)	8
	DM				102	76/26	47.88±4.56			
	Control				102	66/36	47.14±6.38			
**El Nahid2022 [37]**	DKD	Egypt	genome-wide association study	T2DM—DKD	22	NA	NA	rs741301, rs1345365	real-time PCR	6
	DM				23	NA	NA			
	Control				44	NA	NA			

Abbreviations: DKD, diabetic kidney disease; DM, diabetes mellitus; T1/2D, type 1/2 diabetes; NA, not available; SNP, Single Nucleotide Polymorphisms

### Meta-analysis and subgroup analyses

#### Association of DKD and rs741301

**Fig 2:**
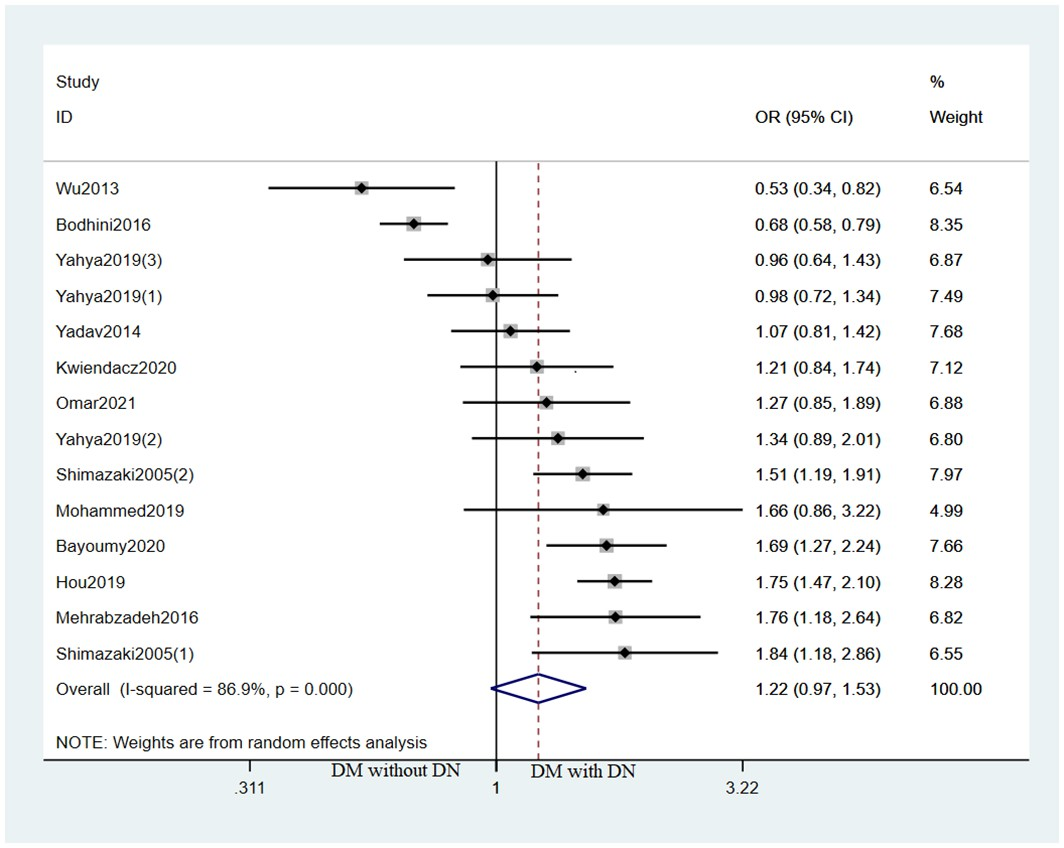
Forest plot of the association between EMLO1 rs741301 polymorphism and DKD risk under the allele model in DKD vs. DM patients.

**Fig 3:**
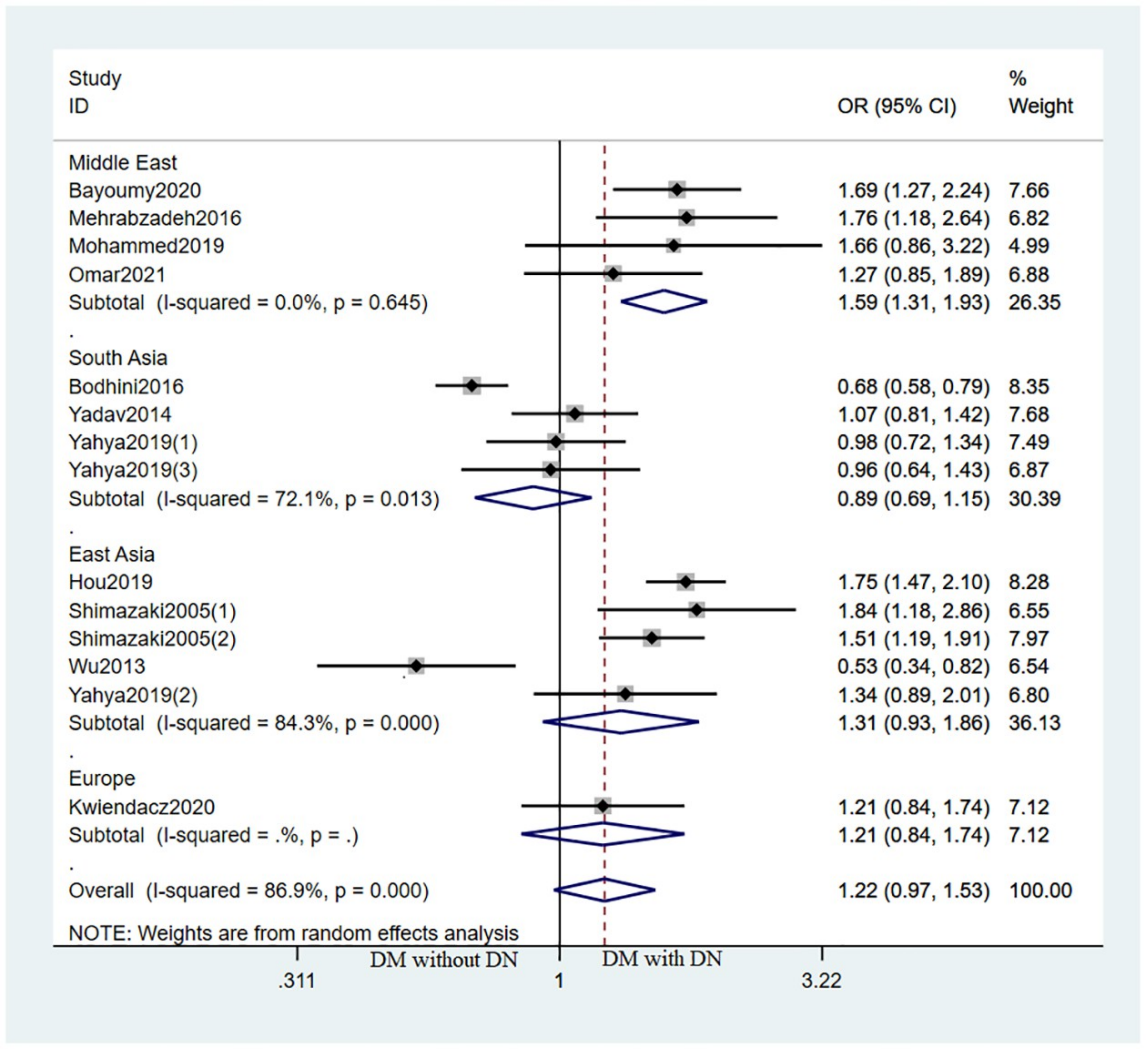
Forest plot of the association between EMLO1 rs741301 polymorphism and DKD risk by region stratification under the allele model in DKD vs. DM patients.

For EMLO1 rs741301 polymorphism, the susceptibility for DKD showed significant difference under the following genetic models in DM with DKD vs. DM without DKD analysis: dominant model (OR [95% CI] = 0.81[0.64,1.01], I^2^ = 74.7%), homozygote model (OR [95% CI] = 1.66[1.09,2.52], I^2^ = 80.2%), recessive model (OR [95% CI] = 1.52[1.07,2.14], I^2^ = 74.7%); while not showing a significant difference in allele model (OR [95% CI] = 1.22[0.97,1.53], I^2^ = 86.9%) and codominant model (OR[95% CI] = 1.15[0.94,1.39], I^2^ = 60.3%) (Fig 2). For studies assessing the relationship between EMLO1 gene polymorphism and DKD risk in different regions, significant increase in DKD risk was identified in Middle East under the allele model (OR [95% CI] = 1.59 [1.31,1.93]), dominant model (AA: OR [95% CI] = 0.60[0.45,0.80]), homozygote model (OR [95% CI] = 2.49[1.57,3.95]) and recessive model (OR [95% CI] = 2.06[1.44,2.95]; no significant association was found among other regions (Fig 3).

In DM with DKD cases vs. healthy controls significant difference under the following genetic models was observed: allele model (OR[95% CI] = 1.73[1.45,2.05]), dominant model (OR[95% CI] = 0.49[0.39,0.61]), codominant model (OR[95% CI] = 1.45[1.12,1.87]), homozygote model (OR[95% CI] = 2.55[1.16,5.61], I^2^ = 77.7%); No conclusive correlation between recessive genetic model was discovered. Increase in DKD risk was detected in Middle East under the allele model (OR [95% CI] = 1.87[1.51,2.31]), dominant model (OR[95% CI] = 0.49[0.36,0.66]), codominant model (OR[95% CI] = 1.62[1.17,2.24]), homozygote model (OR[95% CI] = 3.74[2.13,5.66]), recessive model (OR[95% CI] = 2.37[1.19,4.71], I^2^ = 85.7%).

Under the following genetic models, there were substantial differences between DM patients and healthy controls: allele model (OR [95% CI] = 1.23[0.89,1.69], I^2^ = 67.2%), dominant model (OR [95% CI] = 0.57[0.47,0.68]), and codominant model (OR [95% CI] = 1.29[1.03,1.60]); Other genetic models did not show any conclusive difference. The following models revealed an increase in DKD risk in the Middle East: the allele model (OR [95% CI] = 1.18[0.76,1.82], I^2^ = 73.7%), dominant model (OR [95% CI] = 0.64[0.49,0.83], I^2^ = 41.5%); Other genetic models did not reveal any conclusive associations with other regions (S1-S28 Figs in S1 File). Unlike other studies, Elnahid et al. [37] and Yang et al. [4] reported rs741301 polymorphism under C and T rather than G and A; Hence not being qualified to enter meta-analysis. Regardless, S29 Fig in S1 File shows that even with the incorporation of these studies into the analysis, no significant difference between cases and controls was observed in allele model of DM with DKD vs. DM without DKD patients (OR[95%CI] = 1.18[0.95,1.45]).

#### Association of DKD and rs1345365

Under the following allele genetic model in the study of DM with DKD vs. DM without DKD groups, the susceptibility for DKD for the EMLO1 rs1345365 polymorphism indicated a significant difference (OR[95% CI] = 1.18[1.03,1.36]). A rise in the risk of DKD was noted in East Asia under the allele model (OR[95% CI] = 1.18[1.02,1.37], I^2^ = 60.7%).

Significant differences were seen between DM with DKD patients and healthy controls under the allele genetic model (OR[95% CI] = 0.84[0.74,0.94]). Significant differences were found between DM cases and healthy controls in the allele genetic model (OR [95% CI] = 0.83[0.74,0.93]). Hanson et al. [26] also further corroborated our result by reporting that rs1345365 polymorphism is strongly associated with DKD (OR [95%CI] = 2.42[1.35,4.32] per copy of A allele; P = 0.001) (S30-S43 Figs in S1 File).

#### Association of DKD and rs10255208

There was a noticeable difference in the susceptibility to DKD for the EMLO1 rs10255208 polymorphism under the allele genetic model in DM with DKD vs. DM without DKD analysis (OR [95% CI] = 1.34[1.02,1.74], I^2^ = 82.2%) (S44 Fig in S1 File).

#### Association of DKD and rs7782979

For EMLO1 rs7782979 polymorphism, the susceptibility for DKD showed significant difference under the allele genetic model in DM with DKD vs. DM without DKD analysis (OR [95% CI] = 1.19 [1.06,1.32]) (S45 Fig in S1 File).

#### Heterogeneity analysis

**Fig 4:**
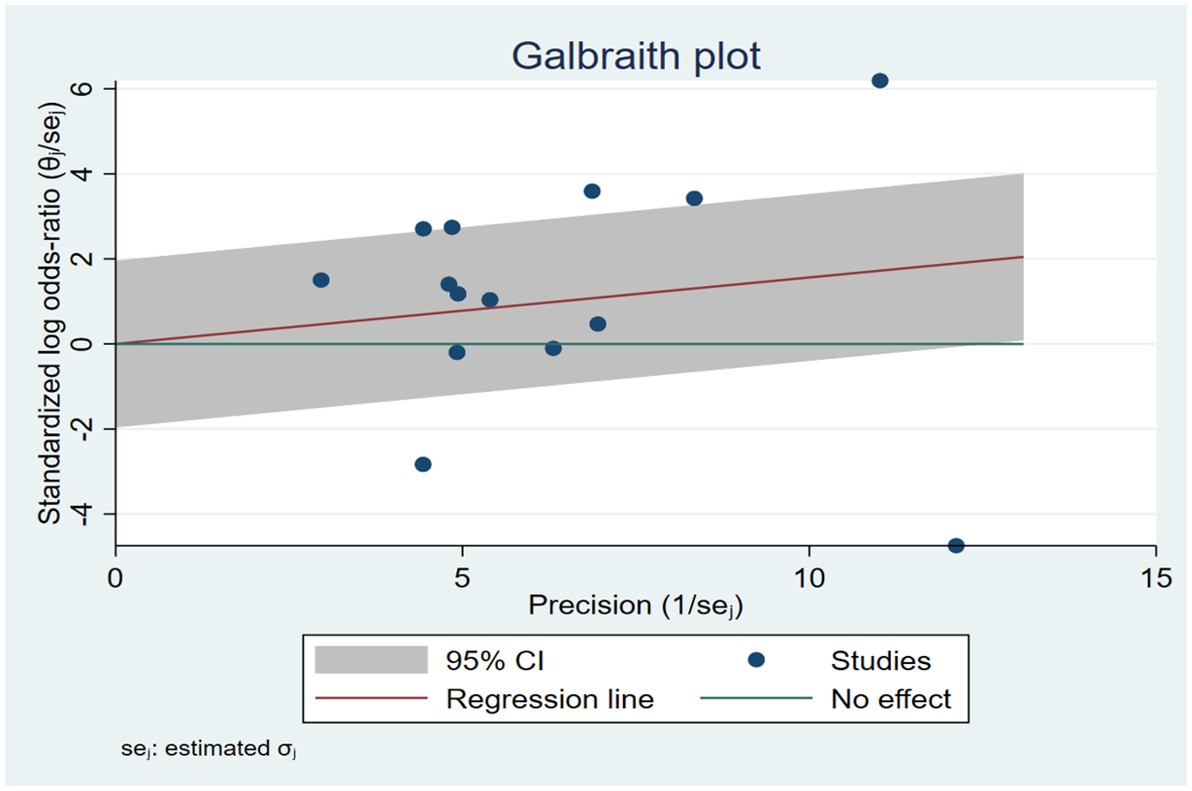
Galbraith plot used to address heterogeneity of EMLO1 rs741301 polymorphism and DKD risk under the allele model in DKD vs. DM patients.

Subgroup analysis was conducted for each haplotype to justify heterogeneity. When failing to do so, analyses of Galbraith plot were used to investigate the sources of heterogeneity (Fig 4). In rs741301 under allele model in DM vs. healthy control, when Omar 2021 [29] was omitted, heterogeneity significantly decreased (I^2^ = 0%; P = 0.84). Hou 2019 [38], Bodhini 2016 [35], and Wu 2013 [30] were found to be contributors of heterogeneity for rs741301 codominant model in DKD vs. DM group, since heterogeneity reduced after exclusion (I^2^ = 0%; P = 0.83) (S46-S65 Figs in S1 File).

#### Sensitivity analysis

**Fig 5:**
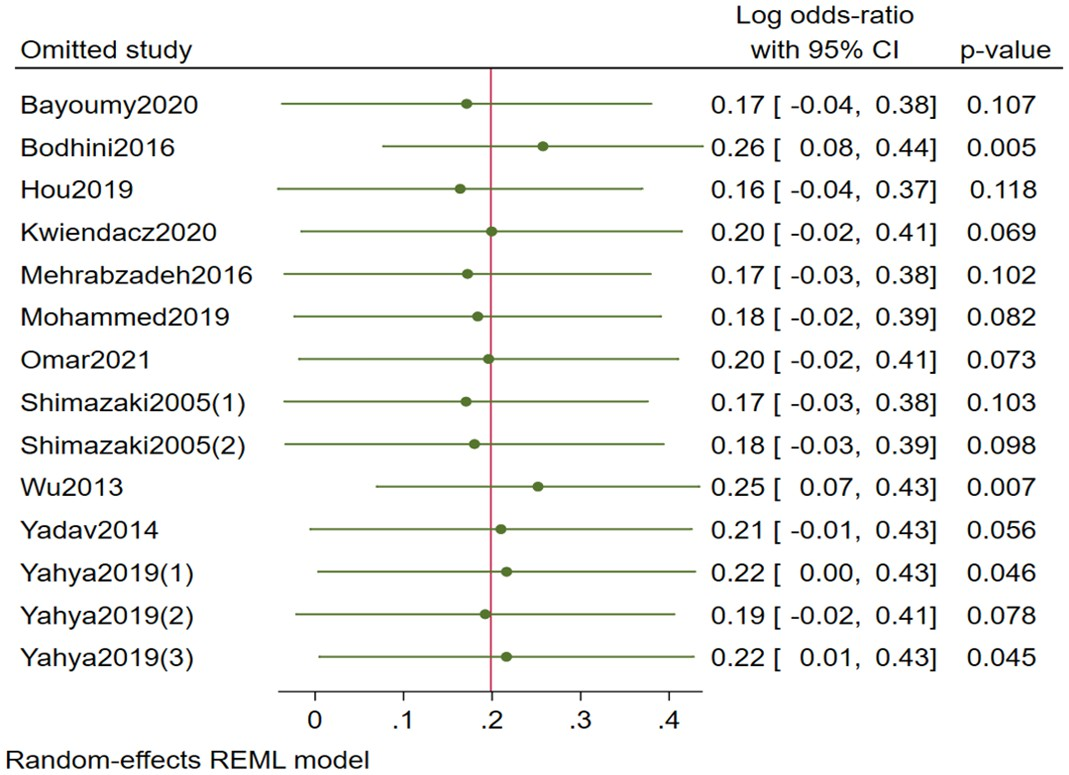
Sensitivity analysis of EMLO1 rs741301 polymorphism and DKD risk under the allele model in DKD vs. DM patients.

Sensitivity analyses were performed to evaluate each study’s impact on the total pooled OR rs741301 polymorphism of DKD vs. DM under allele model with the omission of Bodhini 2016 [35] (OR [95% CI] = 1.29[1.09,1.54], I^2^ = 71.7%) and codominant model with the omission of Wu 2013 [30] (OR [95% CI] = 1.20[1.01,1.44], I^2^ = 51%), and dominant model with the omission of Bodhini 2016 [35] (OR [95% CI] = 0.71[0.60,0.83]) were found to be associated with DKD (Fig 5). Also, after omitting Yadav [31], DM vs. healthy under homozygote model (OR [95% CI] = 2.11[1.03,4.32], I^2^ = 62%) and DKD vs. healthy under recessive model (OR [95% CI] = 2.37[1.19, 4.72], I^2^ = 58%) showed obvious evidence of a strong association. After excluding Omar 2021 [29], rs741301 polymorphism of DKD vs. healthy under codominant model (OR [95% CI] = 1.43[0.97,2.11]) and homozygote model (OR [95% CI] = 1.02[0.95,3.88], I^2^ = 67%) as well as DM vs. healthy (OR [95% CI] = 1.19[0.79,1.78]) showed no significant correlation with DKD. Under codominant model (OR [95% CI] = 1.25[1.04,1.51]) and homozygote model (OR[95% CI] = 1.43[1.01,2.04]) of rs1345365 polymorphism, with the exclusion of Wu 2013 [30], significant association with DKD was detected. The omission of any studies under other genetic models had no discernible impact, demonstrating the statistical reliability of this meta-analysis (S66-S75 Figs in S1 File).

#### Risk of bias and publication bias

**Fig 6:**
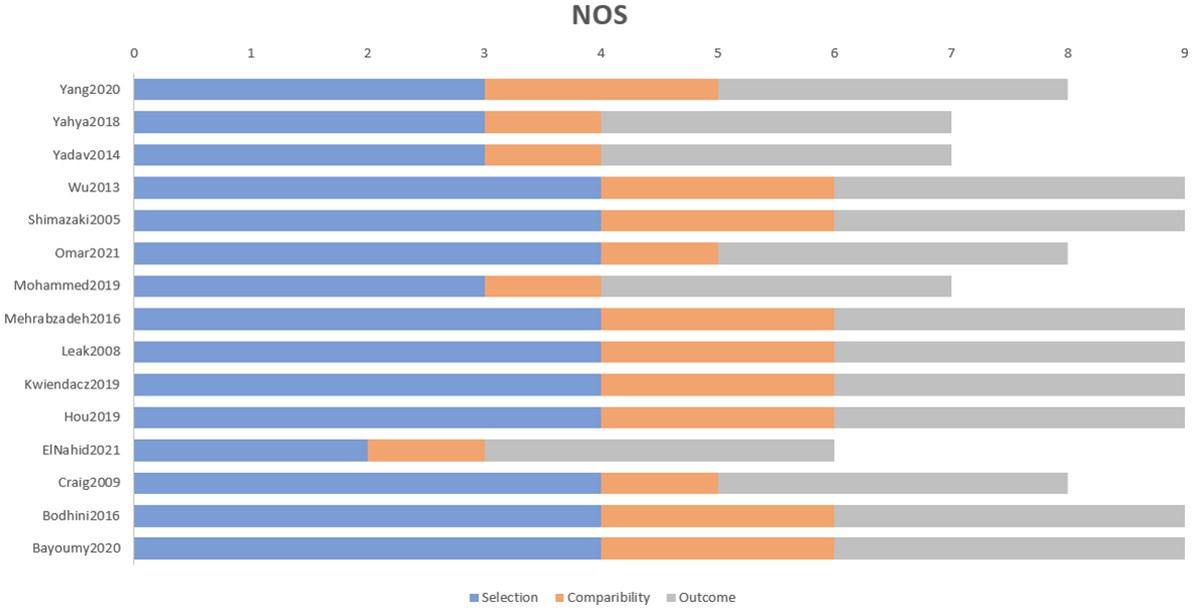
NOS stacked bar diagram of the included studies in meta-analysis.

**Fig 7:**
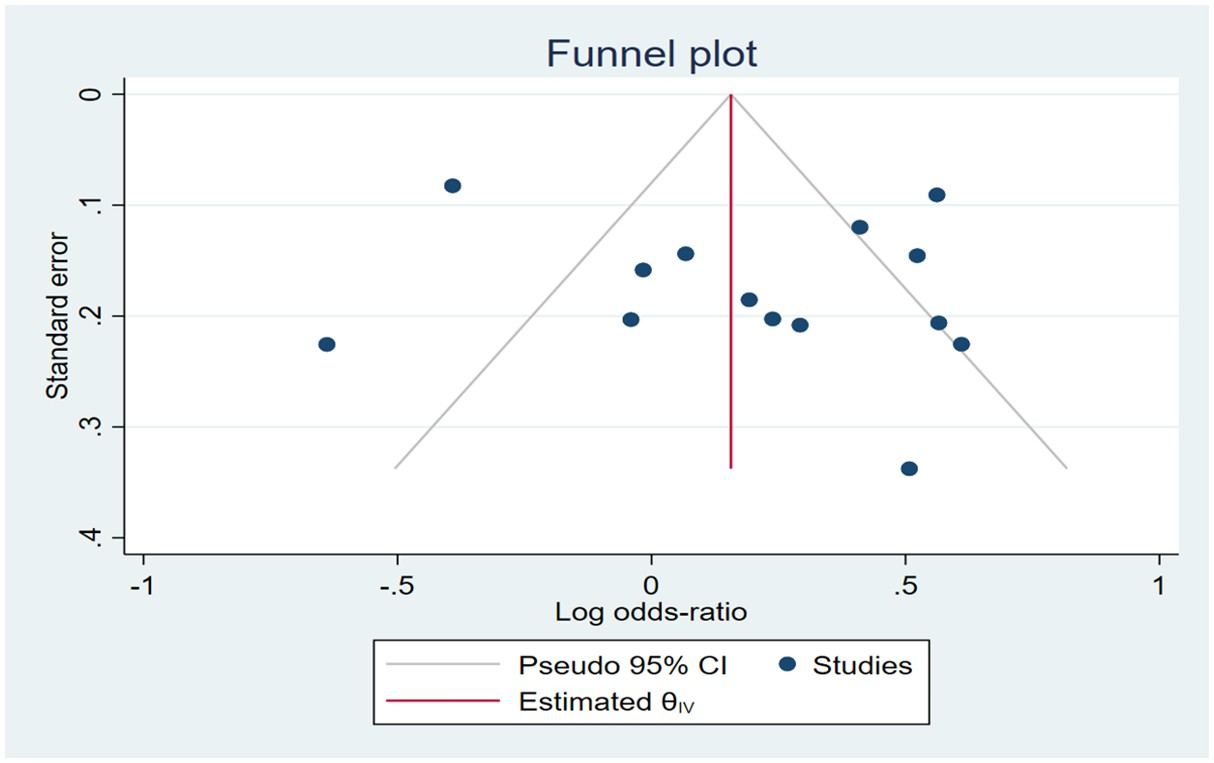
Begg’s funnel plot of publication bias for the association between EMLO1 rs741301 polymorphisms and DKD risk under the allele models in DKD vs. DM patients.

Higher risk of bias is associated with NOS of 6 or less, as one study in our review received 6 [37] on account of lacking quality in the selection section. Lower risk of bias is achieved once studies reach the score 7 or better, which most studies did with the mean NOS of 8.23±1.01 (Fig 6). As for publication bias, All the shapes of the funnel plots were found to be symmetrical, indicating that there was a lack of publication bias for the association of EMLO1 variants polymorphism in all the genetic models (Fig 7 and S76-S96 Figs in S1 File).

### Systematic review of other haplotypes giving rise to DKD

A few of the other haplotypes whose data was not enough to undergo meta-analysis and had significant impact on susceptibility to DKD are systematically reviewed as follows: rs6462776 (OR[95%CI] = 1.24[1.03–1.49]; P = 0.025), rs6462777 (OR[95%CI] = 1.29[1.07–1.55]; P = 0.0076) [36]; rs10255208-GG genotype (OR[95%CI] = 1.41[1.06–1.92]; P<0.001) [38]; rs9969311 (OR[95%CI] = 1.32[1.11–1.57]; P = 0.002) [27]; rs11769038 (OR[95%CI] = 1.24[1.09–1.42]; P = 0.0017), rs1882080 (OR[95%CI] = 1.23[1.08–1.41]; P = 0.0032), and rs7799004 (OR[95%CI] = 1.23[1.04–1.46]; P = 0.02) [7]; Intron 16+105608 (C/T) (OR[95%CI] = 1.38[1.11–1.70]; P = 0.003) [11]; rs7782590 (OR[95%CI] = 2.13 [1.12–4.07]; P = 0.0133), rs1981740 (OR[95%CI] = 1.86[1.03–3.38]; P = 0.0319), rs6462733 (OR[95%CI] = 2.21 [1.09–4.50]; P = 0.0185) [26].

As for rs10951509, our meta-analysis revealed no significant association, mainly because of the paucity and heterogeneity of studies; Hanson et al. (OR[95% CI] = 2.42[1.31–4.48] per copy of the A allele; P = 0.002) [26], Leak et al. (OR[95% CI] = 0.81[0.72–0.92]; P = 0.004) [27], and Yang et al. (OR[95% CI] = 1.47[1.08, 2.00]; P = 0.004) [4] reported a positive association between the rs10951509 and DKD, unlike Wu et al. (OR[95% CI] = -0.57 [-1.05– -0.08]; P = 0.02) [30].

In the current systematic review, the association of *ELMO1* gene polymorphisms and diabetic kidney disease was assessed (Table 2); as GWAS in Japanese type 2 diabetic patients [11], replication studies in GoKinD collection study [7] and American Indian study [26], African American cohorts [27], Chinese population [30] and Indian population [31] validated the crucial function of *ELMO1* as a susceptible gene in DKD. Our meta-analysis was conducted on 15 studies and showed significant increase in DKD risk with variants rs741301, rs1345365 (allele model), rs10255208 (allele model) and rs7782979 (allele model) of *ELMO1* gene in DKD patients vs. DM. However, rs741301 haplotype under allele model, which is a model of great importance, did not show an increase in DKD. In DKD vs. healthy patients, allele, dominant, codominant, and homozygote models showed a statistically significant rise in the risk of DKD. Subgroup analysis was defined as the stratification of data based on the region; eliciting a significant association especially between DKD and rs741301 in Middle East population under especially allele model of DKD vs. DM. In DKD vs. healthy patients, the Middle East population subgroup made the otherwise incongruent recessive model into a statistically significant one. Middle East stratification under homozygote model showed that polymorphism of rs741301 can in fact increase the risk of DKD in DM vs. healthy patients.

**Table 2:** The relationship between EMLO1 polymorphisms models and diabetic kidney disease risk.

Subgroup	Genetic model	Genotype/Allele	Model type	OR[95%CI]	P-meta	I2	P-het
**rs741301 DM with DKD vs. DM without DKD**							
**Overall**	Recessive model (n = 14)	rHOMO vs. Hetero+dHOMO	R	1.52[1.07,2.14]	0.02	74.7%	<0.0001
	Dominant model (n = 14)	dHOMO vs. Hetero+rHOMO	R	0.81[0.64,1.01]	0.05	74.7%	<0.0001
	Codominant model (n = 14)	Hetero vs. dHOMO	R	1.15[0.94,1.39]	0.14	60.3%	<0.0001
	Homozygote model (n = 14)	rHOMO vs. dHOMO	R	1.66[1.09,2.52]	0.02	80.2%	<0.0001
	Allele model (n = 14)	MinAN vs. MajAN	R	1.22[0.97,1.53]	0.05	86.9%	<0.0001
**Middle East**	Recessive model (n = 4)	rHOMO vs. Hetero+dHOMO	R	2.06[1.44,2.95]	<0.05	0.3%	0.39
	Dominant model (n = 4)	dHOMO vs. Hetero+rHOMO	R	0.60[0.45,0.80]	<0.05	0	0.51
	Codominant model (n = 4)	Hetero vs. dHOMO	R	1.41[1.04,1.92]	<0.05	0	0.52
	Homozygote model (n = 4)	rHOMO vs. dHOMO	R	2.49[1.57,3.95]	<0.05	20.7%	0.29
	Allele model (n = 4)	MinAN vs. MajAN	R	1.59 [1.31,1.93]	<0.05	0%	0.65
**East Asia**	Recessive model (n = 5)	rHOMO vs. Hetero+dHOMO	R	2.08[1.09,3.97]	>0.05	72.7%	0.01
	Dominant model (n = 5)	dHOMO vs. Hetero+rHOMO	R	0.79[0.52,1.21]	>0.05	81.2%	<0.0001
	Codominant model (n = 5)	Hetero vs. dHOMO	R	1.13[0.77,1.68]	>0.05	75.8%	<0.0001
	Homozygote model (n = 5)	rHOMO vs. dHOMO	R	2.19[1.01,4.73]	<0.05	79.4%	<0.0001
	Allele model	MinAN vs. MajAN	R	1.31[0.93,1.86]	>0.05	84.3%	<0.0001
**Europe**	Recessive model (n = 1)	rHOMO vs. Hetero+dHOMO	R	1.38[0.66,2.88]	>0.05	NA	NA
	Dominant model (n = 1)	dHOMO vs. Hetero+rHOMO	R	0.82[0.51,1.32]	>0.05	NA	NA
	Codominant model (n = 1)	Hetero vs. dHOMO	R	1.16[0.69,1.94]	>0.05	NA	NA
	Homozygote model (n = 1)	rHOMO vs. dHOMO	R	1.47[0.68,3.20]	>0.05	NA	NA
	Allele model	MinAN vs. MajAN	R	1.21[0.84,1.74]	>0.05	NA	NA
**South Asia**	Recessive model (n = 4)	rHOMO vs. Hetero+dHOMO		0.86[0.67,1.12]		9.8%	
	Dominant model (n = 4)	dHOMO vs. Hetero+rHOMO	R	1.13[0.95,1.35]	>0.05	0.0%	0.42
	Codominant model (n = 4)	Hetero vs. dHOMO	R	0.91[0.75,1.09]	>0.05	0	0.47
	Homozygote model (n = 4)	rHOMO vs. dHOMO	R	0.82[0.62,1.08]	>0.05	9.7%	0.34
	Allele model	MinAN vs. MajAN	R	0.89[0.69,1.15]	>0.05	72.1%	0.01
**rs741301 DM with DKD vs. healthy**							
**Overall**	Recessive model (n = 5)	rHOMO vs. Hetero+dHOMO	R	1.67[0.69,4.06]	0.23	85.7%	<0.0001
	Dominant model (n = 5)	dHOMO vs. Hetero+rHOMO	F	0.49[0.39,0.61]	<0.0001	5.5%	0.38
	Codominant model (n = 5)	Hetero vs. dHOMO	F	1.45[1.12,1.87]	<0.0001	30.9%	0.22
	Homozygote model (n = 5)	rHOMO vs. dHOMO	R	2.55[1.16,5.61]	0.01	77.7%	<0.0001
	Allele model (n = 5)	MinAN vs. MajAN	F	1.73[1.45,2.05]	<0.0001	38.6%	0.16
**Middle East**	Recessive model (n = 4)	rHOMO vs. Hetero+dHOMO	R	2.37[1.19,4.71]	<0.05	85.7%	0.07
	Dominant model (n = 4)	dHOMO vs. Hetero+rHOMO	F	0.49[0.36,0.66]	<0.05	29.1%	0.24
	Codominant model (n = 4)	Hetero vs. dHOMO	F	1.62[1.17,2.24]),	<0.05	30.9%	0.19
	Homozygote model (n = 4)	rHOMO vs. dHOMO	R	3.74[2.13,5.66]	<0.05	7.2%	0.36
	Allele model (n = 4)	MinAN vs. MajAN	F	1.87[1.51,2.31]	<0.05	40.4%	0.17
**South Asia**	Recessive model (n = 1)	rHOMO vs. Hetero+dHOMO	R	0.61[0.40,0.94]	<0.05	NA	NA
	Dominant model (n = 1)	dHOMO vs. Hetero+rHOMO	F	0.49[0.35,0.69]	<0.05	NA	NA
	Codominant model (n = 1)	Hetero vs. dHOMO	F	1.21[0.80,1.84]	>0.05	0	NA
	Homozygote model (n = 1)	rHOMO vs. dHOMO	R	0.96[0.60,1.52]	>0.05	NA	NA
	Allele model (n = 1)	MinAN vs. MajAN	F	1.48[1.10,2.00]	<0.05	0	NA
**rs741301 DM vs. healthy**							
**Overall**	Recessive model (n = 5)	rHOMO vs. Hetero+dHOMO	R	1.28[0.57,2.86]	0.55	85.9%	<0.0001
	Dominant model (n = 5)	dHOMO vs. Hetero+rHOMO	F	0.57[0.47,0.68]	<0.0001	40.1%	0.15
	Codominant model (n = 5)	Hetero vs. dHOMO	F	1.29[1.03,1.60]	0.02	0	0.43
	Homozygote model (n = 5)	rHOMO vs. dHOMO	R	1.68[0.86,3.29]	0.13	76.4%	<0.0001
	Allele model (n = 5)	MinAN vs. MajAN	R	1.23[0.89,1.69]	<0.0001	67.2%	0.04
**Middle East**	Recessive model (n = 4)	rHOMO vs. Hetero+dHOMO	R	1.66[0.76,3.63]	>0.05	71.8%	0.01
	Dominant model (n = 4)	dHOMO vs. Hetero+rHOMO	F	0.64[0.49,0.83]	<0.05	41.5%	0.16
	Codominant model (n = 4)	Hetero vs. dHOMO	F	1.34[1.02,1.77]	<0.05	15.5%	0.31
	Homozygote model (n = 4)	rHOMO vs. dHOMO	R	2.11[1.03,4.30]	<0.05	61.3%	0.05
	Allele model (n = 4)	MinAN vs. MajAN	R	1.18[0.76,1.82]	<0.05	73.7%	0.02
**South Asia**	Recessive model (n = 1)	rHOMO vs. Hetero+dHOMO	R	0.57[0.40,0.80]	<0.05	NA	NA
	Dominant model (n = 1)	dHOMO vs. Hetero+rHOMO	F	0.50[0.39,0.66]	<0.05	0	NA
	Codominant model (n = 1)	Hetero vs. dHOMO	F	1.20[0.84,1.71]	>0.05	0	NA
	Homozygote model (n = 1)	rHOMO vs. dHOMO	R	0.89[0.62,1.27]	>0.05	NA	NA
	Allele model (n = 1)	MinAN vs. MajAN	R	1.39[1.03,1.86]	<0.05	NA	NA
**rs1345365 DM with DKD vs. DM without DKD**							
**Overall**	Recessive model (n = 5)	rHOMO vs. Hetero+dHOMO	F	1.28[0.92,1.78]	0.14	0	0.7
	Dominant model (n = 5)	dHOMO vs. Hetero+rHOMO	F	0.97[0.83,1.12]	0.66	0	0.99
	Codominant model (n = 5)	Hetero vs. dHOMO	F	1.18[0.98,1.41]	0.08	22.2%	0.27
	Homozygote model (n = 5)	rHOMO vs. dHOMO	F	1.37[0.98,1.92]	0.07	0	0.58
	Allele model (n = 5)	MinAN vs. MajAN	F	1.18 [1.03,1.36]	0.02	30.6%	0.22
**Middle East**	Recessive model (n = 2)	rHOMO vs. Hetero+dHOMO	F	1.15[0.43,3.07]	>0.05	35%	0.21
	Dominant model (n = 2)	dHOMO vs. Hetero+rHOMO	F	0.97[0.61,1.55]	>0.05	0	0.76
	Codominant model (n = 2)	Hetero vs. dHOMO	F	1.29[0.76,2.19]	>0.05	0	0.86
	Homozygote model (n = 2)	rHOMO vs. dHOMO	F	1.27[0.46,3.49]	>0.05	30.30%	0.23
	Allele model (n = 2)	MinAN vs. MajAN	F	1.18[1.02,1.37]	>0.05	60.7%	0.41
**East Asia**	Recessive model (n = 3)	rHOMO vs. Hetero+dHOMO	F	1.30[0.91,1.84]	>0.05	0	0.73
	Dominant model (n = 3)	dHOMO vs. Hetero+rHOMO	F	0.97[0.82,1.13]	>0.05	0	0.91
	Codominant model (n = 3)	Hetero vs. dHOMO	F	1.16[0.96,1.41]	>0.05	59.9%	0.08
	Homozygote model (n = 3)	rHOMO vs. dHOMO	F	1.38[0.97,1.98]	>0.05	0	0.49
	Allele model (n = 3)	MinAN vs. MajAN	F	1.18 [1.02,1.37]	<0.05	60.7%	0.08
**rs1345365 DM with DKD vs. healthy**							
**Overall**	Recessive model	rHOMO vs. Hetero+dHOMO	F	0.80[0.43,1.6]	0.50	0	0.48
	Dominant model	dHOMO vs. Hetero+rHOMO	F	1.05[0.72,1.54]	0.83	0	0.89
	Codominant model	Hetero vs. dHOMO	F	0.97[0.69,1.48]	0.71	0	0.7
	Homozygote model	rHOMO vs. dHOMO	F	0.79[0.42,1.48]	0.45	0	0.55
	Allele model (n = 4)	MinAN vs. MajAN	F	0.84[0.74,0.94]).	<0.0001	0%	0.95
**Middle East**	Allele model (n = 2)	MinAN vs. MajAN	F	0.86[0.59,1.27]	>0.05	0	0.8
**USA**	Allele model (n = 1)	MinAN vs. MajAN	F	0.83[0.74,0.94]	<0.05	0	NA
**rs1345365 DM vs. healthy**							
**Overall**	Recessive model	rHOMO vs. Hetero+dHOMO	F	0.82[0.45,1.35]	0.39	0	0.92
	Dominant model	dHOMO vs. Hetero+rHOMO	F	1.07[0.77,1.36]	0.78	0	0.97
	Codominant model	Hetero vs. dHOMO	F	0.85[0.68,1.19]	0.32	0	0.72
	Homozygote model	rHOMO vs. dHOMO	F	0.74[0.44,1.27]	0.29	0	0.84
	Allele model (n = 3)	MinAN vs. MajAN	F	0.83[0.74,0.93]	<0.0001	0	0.94
**Middle East**	Allele model (n = 2)	MinAN vs. MajAN	F	0.80[0.58,1.11]	>0.05	0	0.8
**USA**	Allele model (n = 1)	MinAN vs. MajAN	F	0.83[0.74,0.94]	<0.05	0	NA
**rs10255208 DM with DKD vs. DM without DKD**							
**Overall**	Allele model (n = 2)	MinAN vs. MajAN	R	1.34[1.02,1.74]	0.03	82.2%	0.02
**rs7782979 DM with DKD vs. DM without DKD**							
**Overall**	Allele model (n = 2)	MinAN vs. MajAN	F	1.19 [1.06,1.32]	<0.0001	0	0.8

Abbreviations: OR, odds ratio; CI, confidence interval; R, random effect model; F, fixed effect model; P-met, P of meta-analysis; P-het, P of heterogeneity; NA, not available; MinAN, minor allele number; MajAN, major allele number; rHOMO, recessive homozygote; dHOMO, dominant homozygote; Hetero, heterozygote; DKD, diabetic kidney disease; DM, diabetes mellitus

High heterogeneity was detected across our analyses; the interpretation of which was facilitated through subgroup analysis by region. For example, for EMLO1 rs741301 polymorphism under the allele model in DKD vs. DM patients, the test for subgroup differences suggests that there is a statistically significant subgroup effect (p = 0.00) and no heterogeneity was observed in Middle East (I^2^ = 0%). However due to the unknown pathogenesis of DKD, studies which caused this heterogeneity [34, 35] were unable to specifically justify the disagreements of their findings with other studies; thus resorting to general statements such as genetic and environmental factors, racial differences, ethnic confounders, and small sample sizes.

The *ELMO1* gene, which spans about 590 kb on chromosome 7p14.2–14.1 and has 22 exons and 21 introns, codes for a protein that belongs to the family of engulfment and cell motility proteins. Although the precise role of the *ELMO1* gene in the pathophysiology and development of DKD is unclear, it can be explained by a variety of methods. *ELMO1* gene expression up-regulation was found to cause more hyperglycemia [39, 40]. It has been hypothesized that *ELMO1* is overexpressed in the serum of DKD patients, giving rise to a process that may cause a downregulation of the metalloproteinase gene, cell adhesion, and upregulation of extracellular matrix gene, leading to the progression of DKD. It was proposed that RAC-1 integration with Dock180 may be activated by *ELMO1*, increasing the expression of the extracellular matrix gene [10]. Numerous studies demonstrated the impact of the *ELMO1* gene on the onset and progression of DKD, which may be explained by a variety of processes, including damage to the renal tissue caused by the gene’s effect on the production of reactive oxygen species [9]. Another method is how *ELMO1* stimulates the TGF-β1 gene, which causes fibrosis, and inhibits the matrix metalloproteinase gene, which in turn prevents fibrosis. It causes glomerular basement membrane thickening and exacerbates the glomerulosclerosis brought on by DM in mice [9, 11]. In order to start and maintain glomerular damage that results in glomerulosclerosis, it also interacts with cyclooxygenase-2 [41]. It is conceivable for random associations to happen haphazardly, or just by coincidence. The "flip-flop" phenomenon illustrates how distinct association patterns can emerge between populations as a result of varying linkage disequilibrium patterns that lead to the occurrence of functional variations on various haplotypes [42]. It is interesting to notice that linkage disequilibrium spans over significantly wider areas in Pima Indians [26] compared with African Americans [27]. Furthermore, while the A allele at rs1345365 is the minor allele in African Americans, it is the major allele in Pimas. One study reported that no significant gene-hypertension interaction combinations were observed, but there was a significant gene-alcohol drinking interaction combination. Those who regularly consume alcohol with rs741301-AG/GG genotype have a higher risk of developing DKD than those who abstain from alcohol with rs741301-AA genotype [38]. According to Shimazaki et al., the sequence containing SNP site of intron 18+1970 is compatible with the G-protein-coupled receptor 1 (GCR-1) binding site. GCR-1 was able to regulate the transcriptional activity of glycolytic enzyme genes in response to adjustments in extracellular glucose concentrations, changing susceptibility to DKD [11].

*ELMO1* polymorphism of rs741301 variant was linked to diabetic kidney disease among Asians with type 2 diabetic kidney disease, according to Mooyaart et al.’s meta-analysis of genetic associations in DKD (OR 1.58 [95% CI 1.28–1.94]); but not with a third study of patients with type 1 diabetes mellitus in Europe. Unlike our overall findings on *ELMO1* gene, Mooyaart et al. did not find a statistically significant association between *ELMO1* rs741301 polymorphism under G allele and DKD in total (p = 0.60) [43].

At last, to provide a comprehensive overview and perform pooled analysis of all data collected during the past years about *ELMO1* and DKD, a systematic review and meta-analysis was conducted. We ran additional analyses and models to address heterogeneity; apart from subgroup analysis, Galbraith plot was particularly helpful when subgroup analysis could not justify high heterogeneity. Sensitivity analysis, through omission of one study, helped us better understand the results as it shifted some of the analyses from showing no association to a statistically significant one, such as rs741301 polymorphism of DKD vs. DM under allele model. The longer the duration of diabetes, the higher the chance of developing DKD; due to significantly affecting kidney function and electrolyte balance. Long-term exposure to hyperglycemia damages the glomerulus, tubulointerstitium, and vasculature either directly or indirectly through hemodynamic alterations, which accounts for the association between DKD and the length of diabetes [28]. Hence, our study can contribute to early genetic screening in order that pre-emptive measures be taken to prevent diabetic kidney disease.

### Limitations

First, the etiology of DKD is complicated and multifactorial. In our investigation, the associations between *ELMO1* gene polymorphisms and other risk factors, such as environmental variables, racial disparities, ethnic confounders, etc., were not examined. Second, due to insufficient data, other *ELMO1* gene polymorphisms, including rs6462776, rs11769038, and rs7799004, were not examined in our investigation. Third, test findings may differ depending on the genotyping techniques employed in the studies. Finally, some of the papers used in this meta-analysis had small samples, which could have impacted the findings and produced inconsistent results.

### Future recommendations

Larger sample sizes and a more varied population should be used in future studies to examine the relationship between *ELMO1* gene polymorphisms and diabetic kidney disease. To better understand the underlying mechanisms, future research should examine the functional significance of *ELMO1* gene polymorphisms in diabetic kidney disease. Additional study is required to examine the possible clinical significance of polymorphisms in the *ELMO1*gene in terms of predicting the onset and course of diabetic kidney disease.

## Conclusion

Overall, with the exception of the allele genetic model in DKD vs. DM patients, show a substantial connection between the *EMLO1* rs741301 polymorphism and DKD susceptibility. However, the Middle East area showed a significantly higher rise in DKD risk for the allele genetic model when population was stratified by region. The susceptibility for DKD for the EMLO1 rs1345365, rs10255208, and rs7782979 polymorphisms pointed to a substantial rise in DKD under the genetic model in DM patients. All *ELMO1* polymorphisms were associated with DKD susceptibility, according to the overall analyses of DKD vs. healthy controls and DM vs. healthy controls.

## Supporting information

S1 Checklist PRISMA 2020 checklist. (DOCX)

S1 File (DOCX)
