# Supplementary material for: Association between ELMO1 gene polymorphisms and diabetic kidney disease: A systematic review and meta-analysis
Source: PLoS One. 2024 Jan 26;19(1):e0295607. doi: 10.1371/journal.pone.0295607 (PMC10817128; doi:10.1371/journal.pone.0295607)
Supplement: S1 File — (DOCX) [file pone.0295607.s002.docx]

| **Table S1.** Genotype distributions. | | | | | | | | | | | | | | | | |
| --- | --- | --- | --- | --- | --- | --- | --- | --- | --- | --- | --- | --- | --- | --- | --- | --- |
| Study | Region | DN with DM | | | | | DN without DM | | | | | Healthy | | | | |
|  |  | MinAN | MajAN | rHOMO | Hetero | dHOMO | MinAN | MajAN | rHOMO | Hetero | dHOMO | MinAN | MajAN | rHOMO | Hetero | dHOMO |
| rs741301 | | | | | | | | | | | | | | | | |
| Bayoumy2020 | Middle East | 186 | 214 | 48 | 90 | 62 | 136 | 264 | 22 | 92 | 86 | 68 | 132 | 10 | 48 | 42 |
| Bodhini2016 | South Asia | 422 | 744 | 82 | 258 | 243 | 594 | 708 | 108 | 278 | 215 | NA | NA | NA | NA | NA |
| Hou2019 | East Asia | 405 | 915 | 70 | 265 | 325 | 268 | 1062 | 33 | 202 | 430 | NA | NA | NA | NA | NA |
| Kwiendacz2020 | Europe | 80 | 154 | 16 | 48 | 53 | 93 | 217 | 16 | 61 | 78 | NA | NA | NA | NA | NA |
| Mehrabzadeh2016 | Middle East | 94 | 106 | 26 | 42 | 32 | 67 | 133 | 12 | 43 | 45 | 71 | 129 | 15 | 41 | 44 |
| Mohammed2019 | Middle East | 37 | 35 | 4 | 29 | 3 | 28 | 44 | 2 | 24 | 10 | 31 | 43 | 6 | 19 | 12 |
| Omar2021 | Middle East | 88 | 112 | 26 | 36 | 38 | 78 | 126 | 22 | 34 | 46 | 44 | 160 | 6 | 32 | 64 |
| Shimazaki2005(1) | East Asia | 72 | 102 | 16 | 40 | 31 | 51 | 133 | 3 | 45 | 44 | NA | NA | NA | NA | NA |
| Shimazaki2005(2) | East Asia | 362 | 556 | 73 | 216 | 170 | 146 | 338 | 13 | 120 | 109 | NA | NA | NA | NA | NA |
| Wu2013 | East Asia | 57 | 189 | 8 | 41 | 74 | 56 | 98 | 10 | 36 | 31 | NA | NA | NA | NA | NA |
| Yadav2014 | South Asia | 151 | 253 | 32 | 87 | 83 | 154 | 276 | 30 | 94 | 91 | 113 | 281 | 113 | 85 | 281 |
| Yahya2019(1) | South Asia | 104 | 158 | 23 | 58 | 50 | 182 | 272 | 37 | 108 | 82 | NA | NA | NA | NA | NA |
| Yahya2019(2) | East Asia | 85 | 131 | 17 | 51 | 40 | 62 | 128 | 9 | 44 | 42 | NA | NA | NA | NA | NA |
| Yahya2019(3) | South Asia | 61 | 111 | 8 | 45 | 33 | 99 | 173 | 20 | 59 | 57 | NA | NA | NA | NA | NA |
| Elnahid2020 | Middle East | 27 | 17 | 9 | 9 | 4 | 36 | 10 | 13 | 10 | 0 | 66 | 22 | 25 | 16 | 3 |
| Yang2019 | East Asia | 143 | 273 | 23 | 97 | 88 | 127 | 273 | 18 | 91 | 91 | NA | NA | NA | NA | NA |
| rs1345365 | | | | | | | | | | | | | | | | |
| Elnahid2020 | Middle East | 10 | 34 | 1 | 8 | 13 | 12 | 34 | 3 | 6 | 14 | 24 | 64 | 5 | 14 | 25 |
| Hou2019 | East Asia | 321 | 999 | 43 | 235 | 382 | 286 | 1044 | 36 | 214 | 415 | NA | NA | NA | NA | NA |
| Leak2009 | USA | 704 | 1566 | NA | NA | NA | NA | NA | NA | NA | NA | 812 | 1508 | NA | NA | NA |
| Mehrabzadeh2016 | Middle East | 57 | 143 | 8 | 41 | 51 | 47 | 153 | 5 | 37 | 58 | 62 | 138 | 9 | 44 | 47 |
| Wu2013 | East Asia | 53 | 193 | 8 | 37 | 78 | 41 | 113 | 5 | 31 | 41 | NA | NA | NA | NA | NA |
| Yang2019 | East Asia | 135 | 281 | 28 | 79 | 101 | 100 | 300 | 18 | 64 | 118 | NA | NA | NA | NA | NA |
| rs10951509 | | | | | | | | | | | | | | | | |
| Leak2009 | USA | 704 | 1566 | NA | NA | NA | NA | NA | NA | NA | NA | 835 | 1485 | NA | NA | NA |
| Wu2013 | East Asia | 42 | 204 | 7 | 28 | 88 | 41 | 113 | 5 | 31 | 41 | NA | NA | NA | NA | NA |
| Yang2019 | East Asia | 304 | 284 | 25 | 82 | 101 | 96 | 304 | 16 | 64 | 120 | NA | NA | NA | NA | NA |
| rs10255208 | | | | | | | | | | | | | | | | |
| Hou2019 | East Asia | 373 | 947 | NA | NA | NA | 271 | 1059 | NA | NA | NA | NA | NA | NA | NA | NA |
| Pezzolesi2009 | USA | 869 | 771 | NA | NA | NA | 867 | 903 | NA | NA | NA | NA | NA | NA | NA | NA |
| rs7782979 | | | | | | | | | | | | | | | | |
| Hou2019 | East Asia | 326 | 994 | NA | NA | NA | 284 | 1046 | NA | NA | NA | NA | NA | NA | NA | NA |
| Pezzolesi2009 | USA | 869 | 771 | NA | NA | NA | 867 | 903 | NA | NA | NA | NA | NA | NA | NA | NA |
| Abbreviations: NA, not available; MinAN, minor allele number; MajAN, major allele number; rHOMO, recessive homozygote; dHOMO, dominant homozygote; Hetero, heterozygote; DN, diabetic nephropathy; DM, diabetes mellitus | | | | | | | | | | | | | | | | |


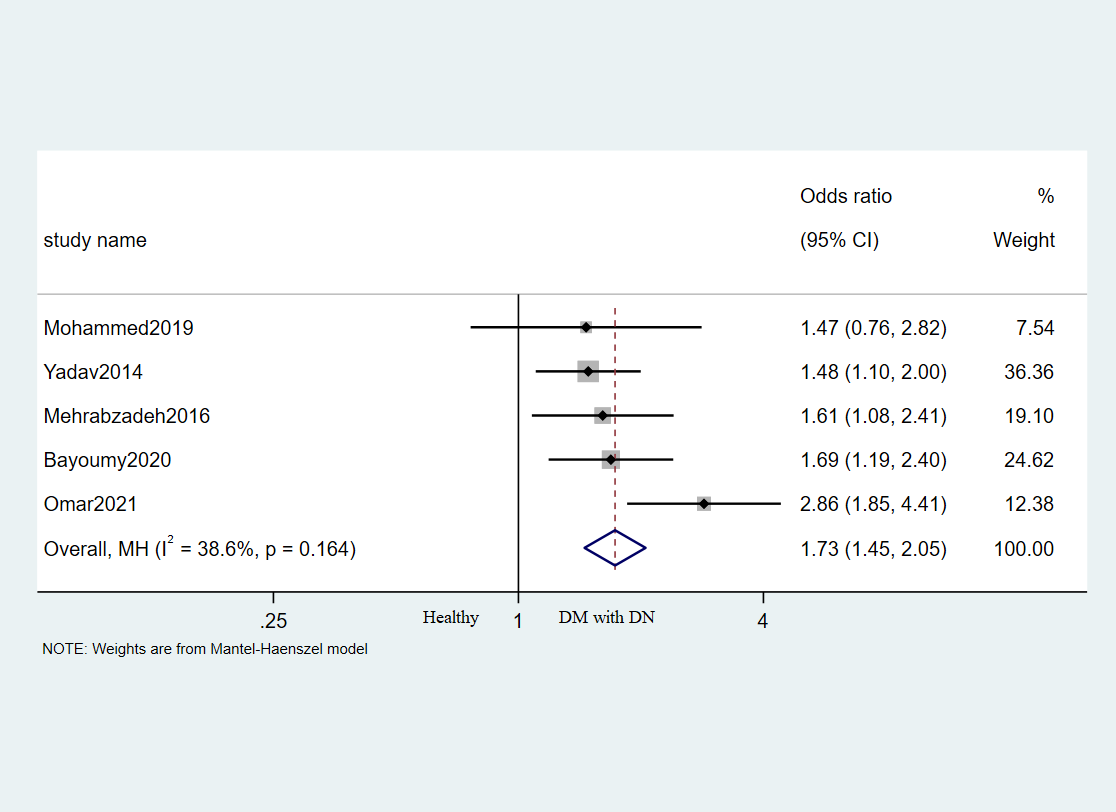


**Figure S1**. **Forest plot of the association between EMLO1 rs741301 polymorphism and DN risk under the allele model in DN vs. healthy patients**


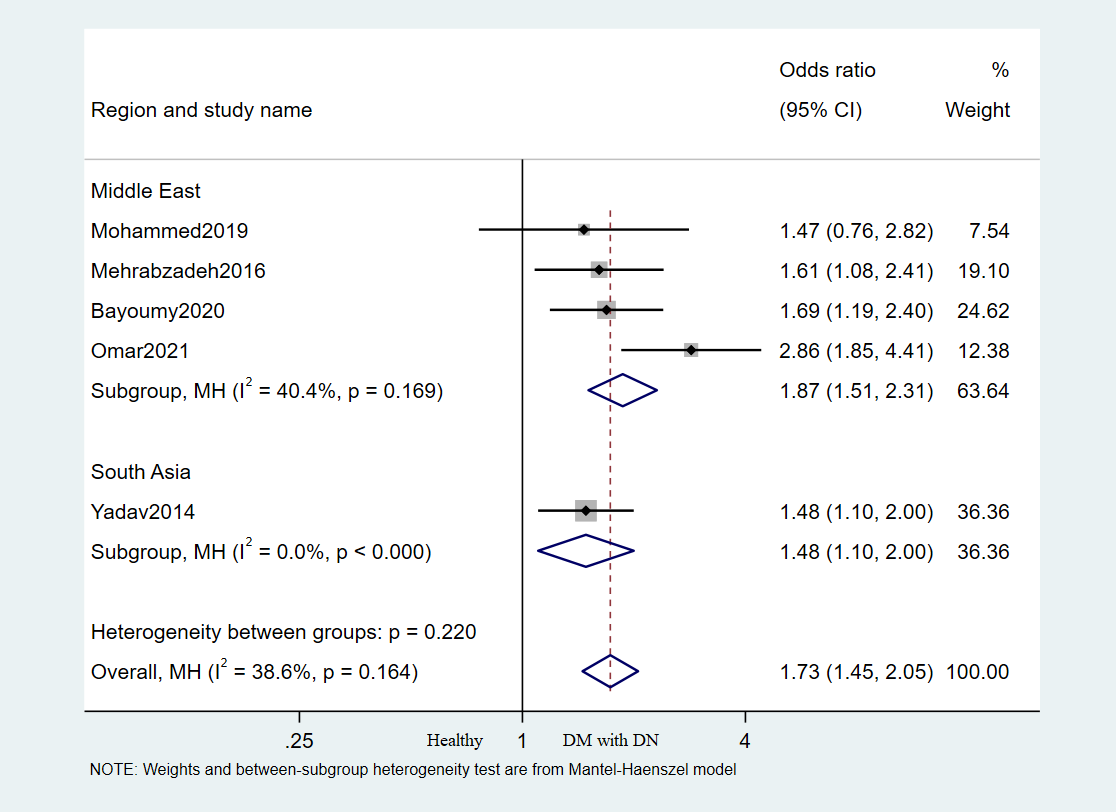


**Figure S2.** **Forest plot of the association between EMLO1 rs741301 polymorphism and DN risk by region stratification under the allele model in DN vs. healthy patients**


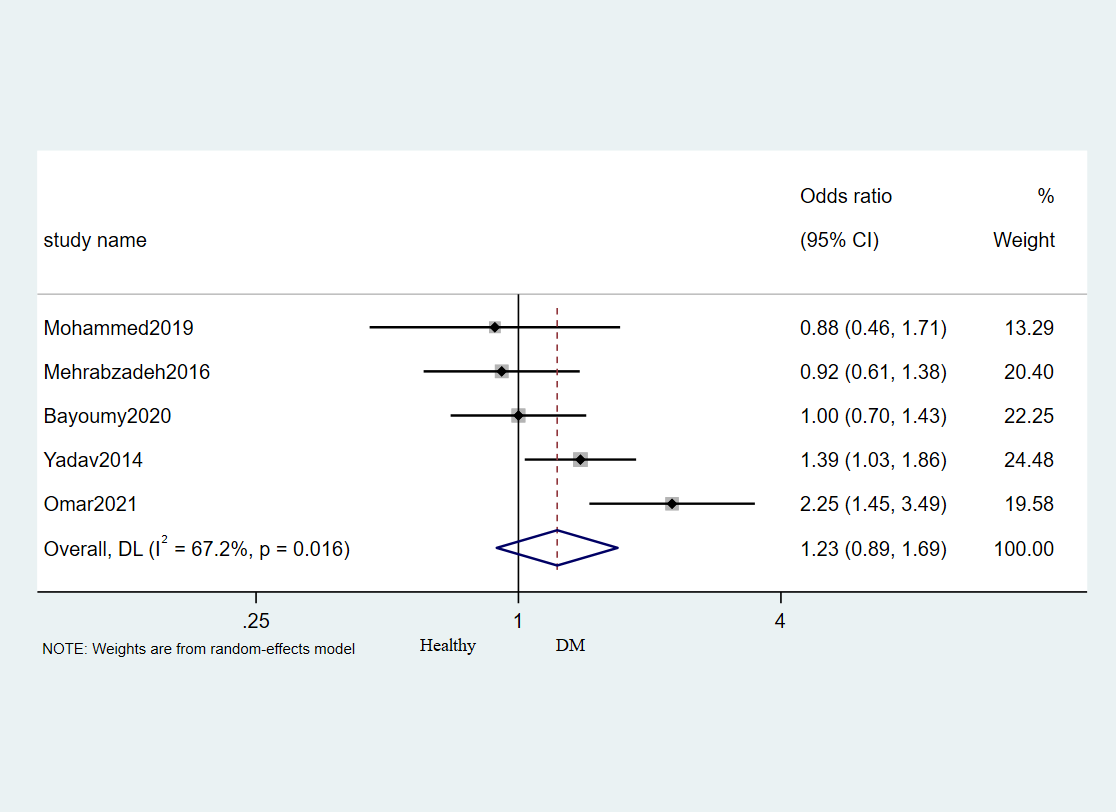


**Figure S3.** **Forest plot of the association between EMLO1 rs741301 polymorphism and DN risk under the allele model in DM vs. healthy patients**


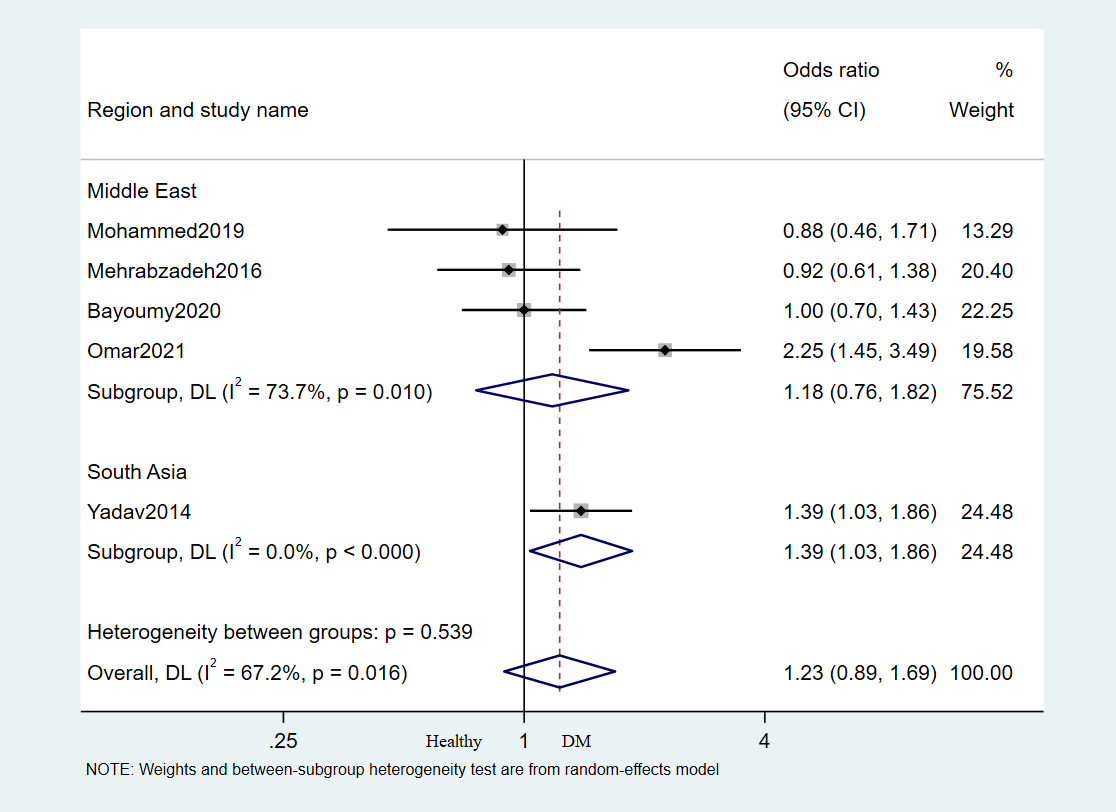


**Figure S4.** **Forest plot of the association between EMLO1 rs741301 polymorphism and DN risk by region stratification under the allele model in DM vs. healthy patients**


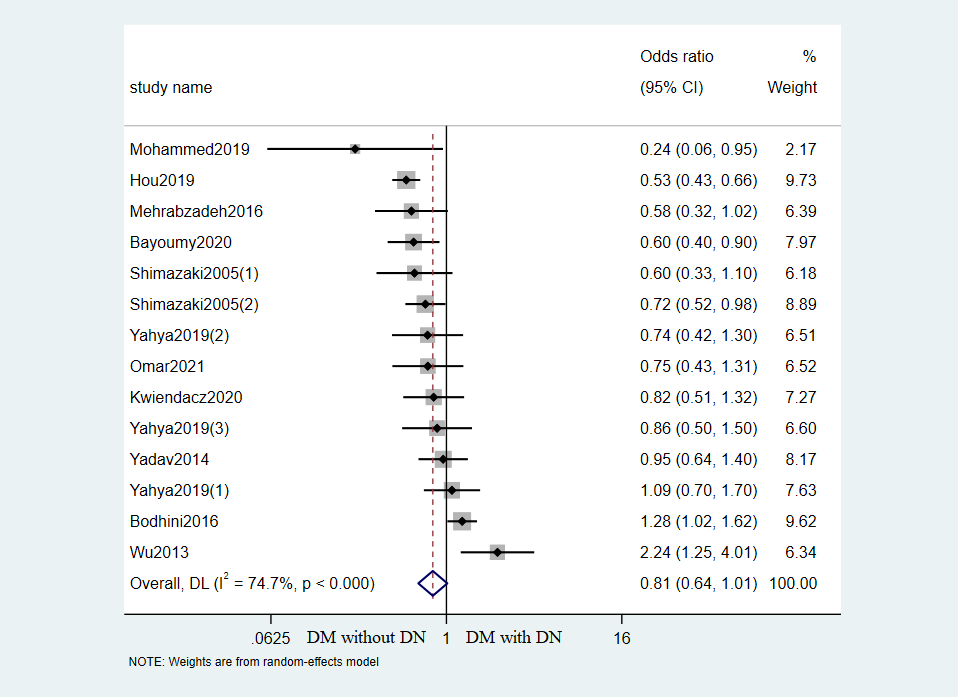


**Figure S5.** **Forest plot of the association between EMLO1 rs741301 polymorphism and DN risk under the dominant model in DN vs. DM patients**


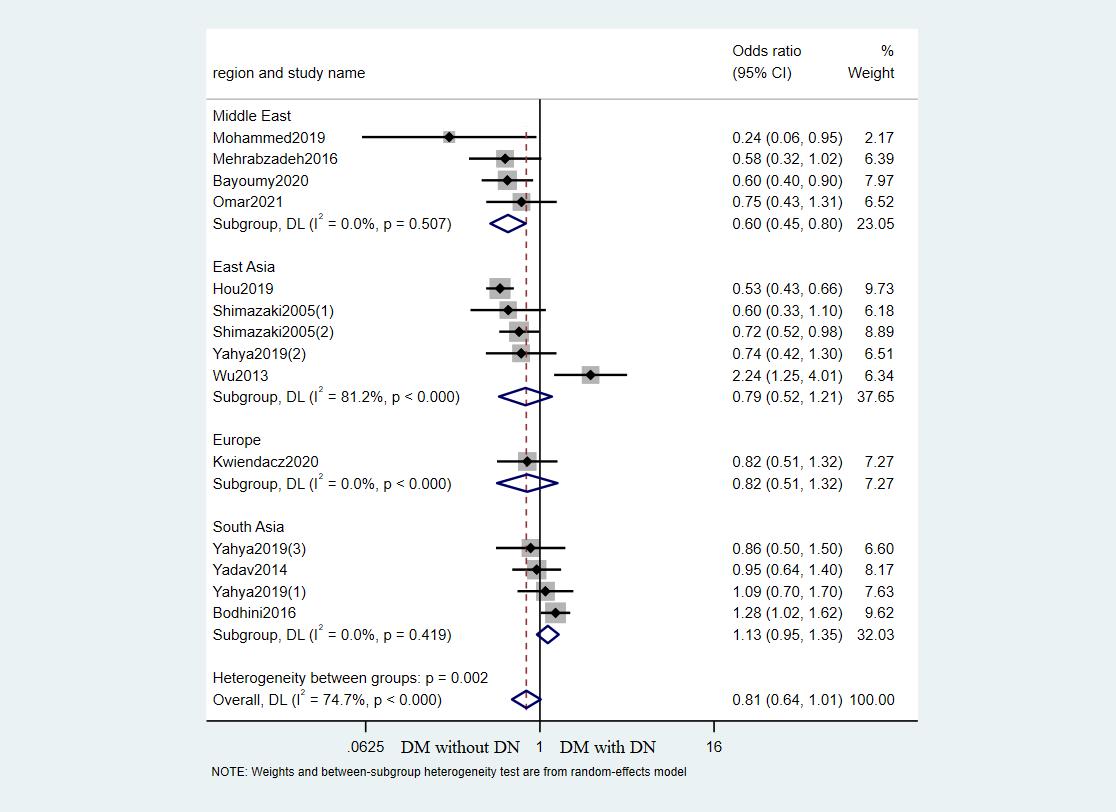


**Figure S6.** **Forest plot of the association between EMLO1 rs741301 polymorphism and DN risk by region stratification under the dominant model in DN vs. DM patients**


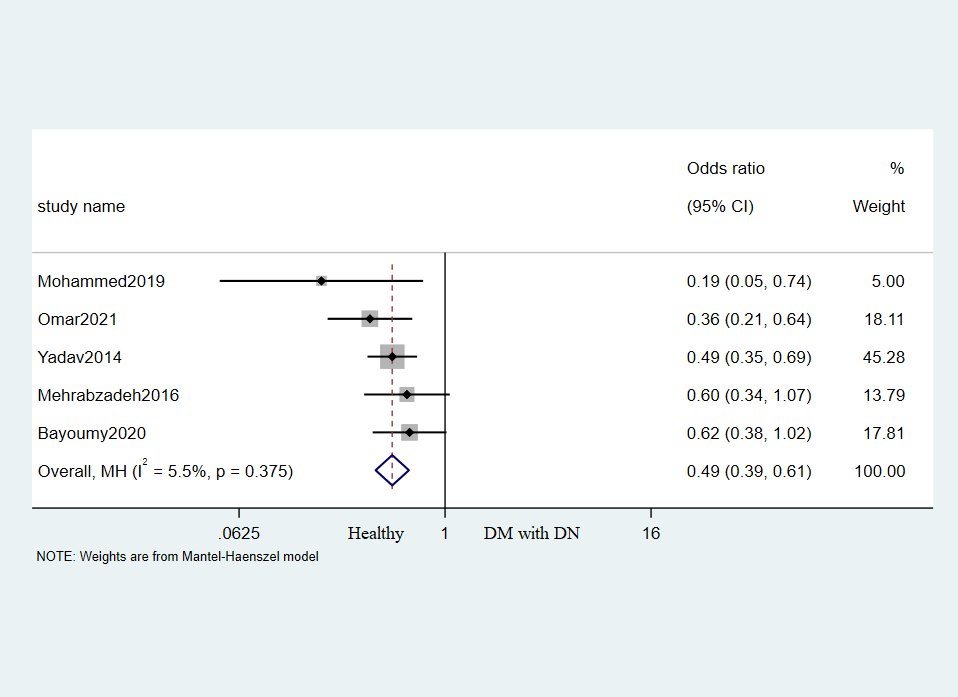


**Figure S7.** **Forest plot of the association between EMLO1 rs741301 polymorphism and DN risk under the dominant model in DN vs. healthy patients**


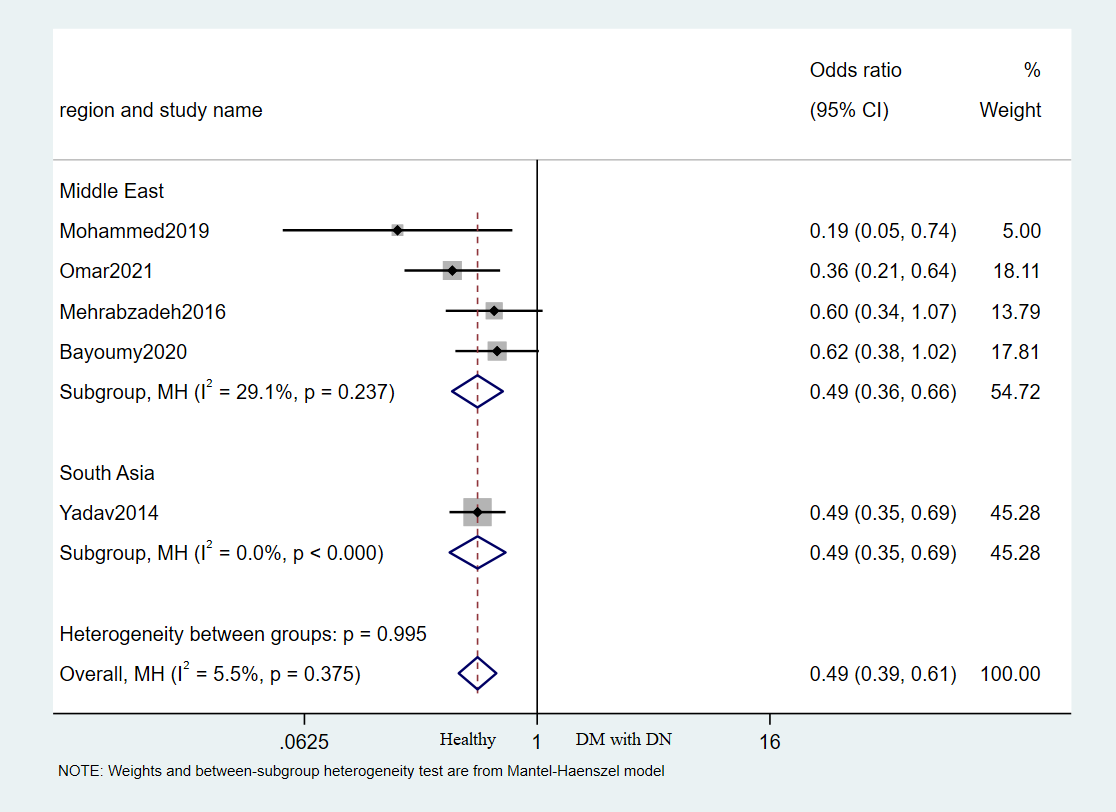


**Figure S8.** **Forest plot of the association between EMLO1 rs741301 polymorphism and DN risk by region stratification under the dominant model in DN vs. healthy patients**


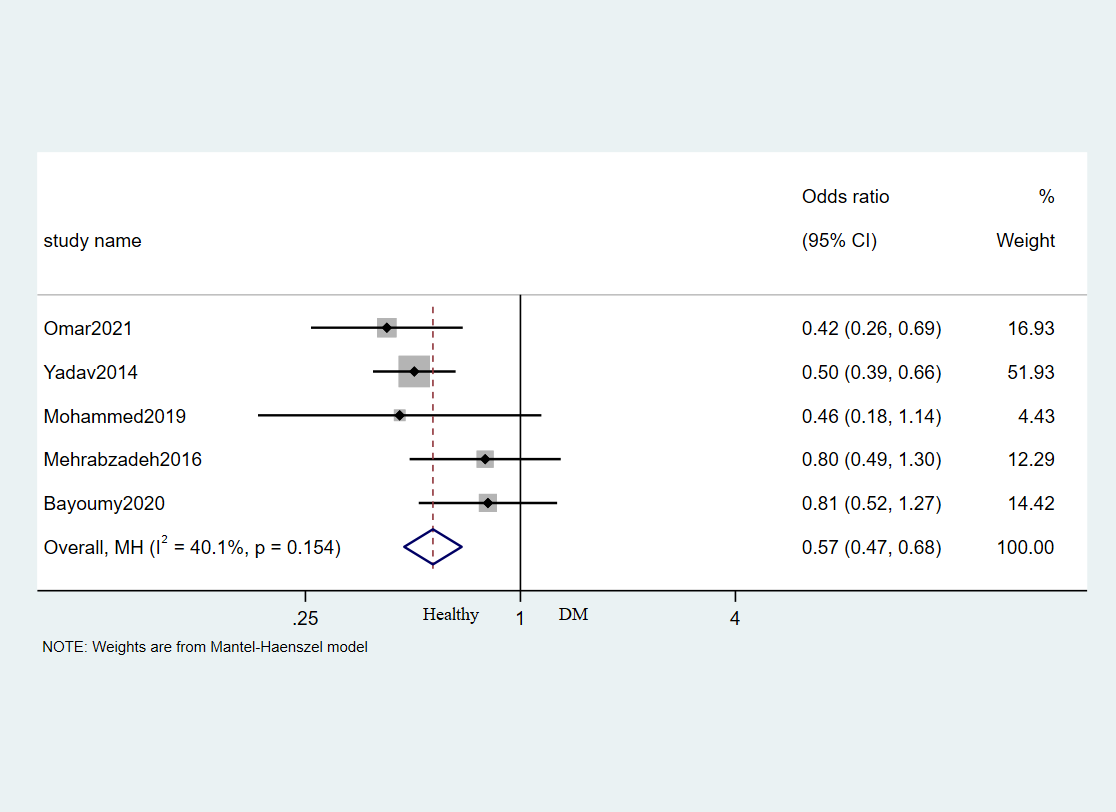


**Figure S9. Forest plot of the association between EMLO1 rs741301 polymorphism and DN risk under the dominant model in DM vs. healthy patients**


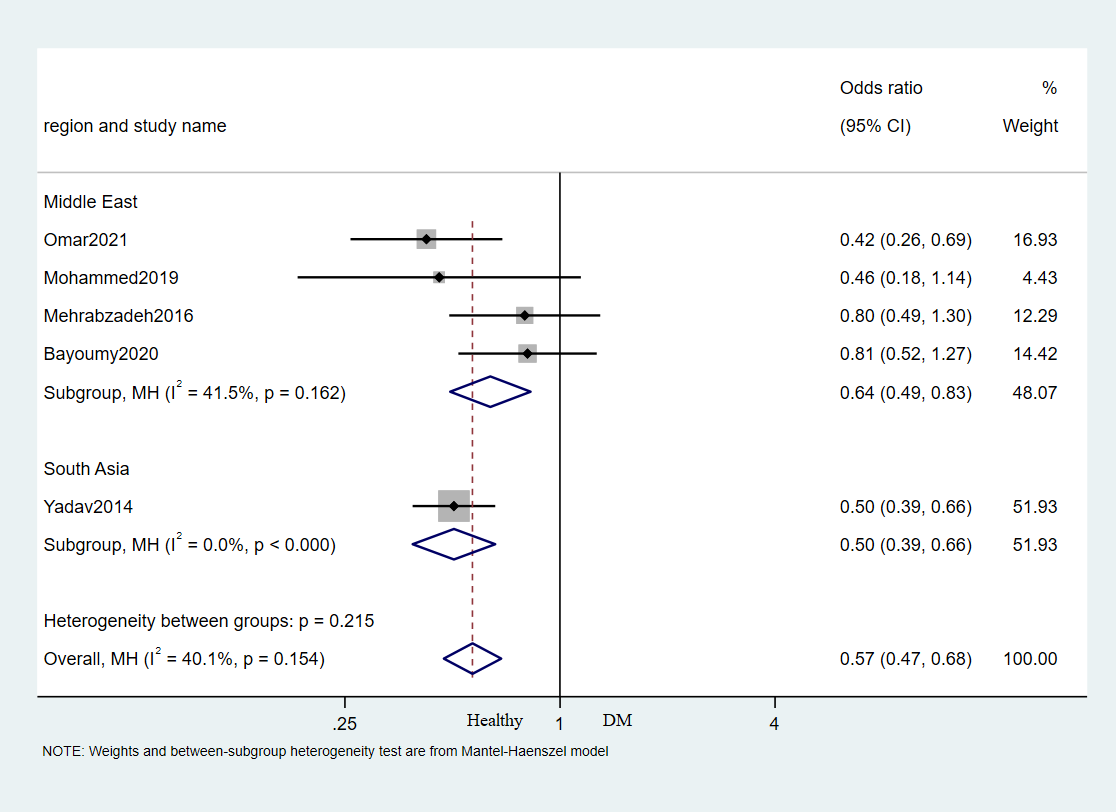


**Figure S10.** **Forest plot of the association between EMLO1 rs741301 polymorphism and DN risk by region stratification under the dominant model in DM vs. healthy patients**


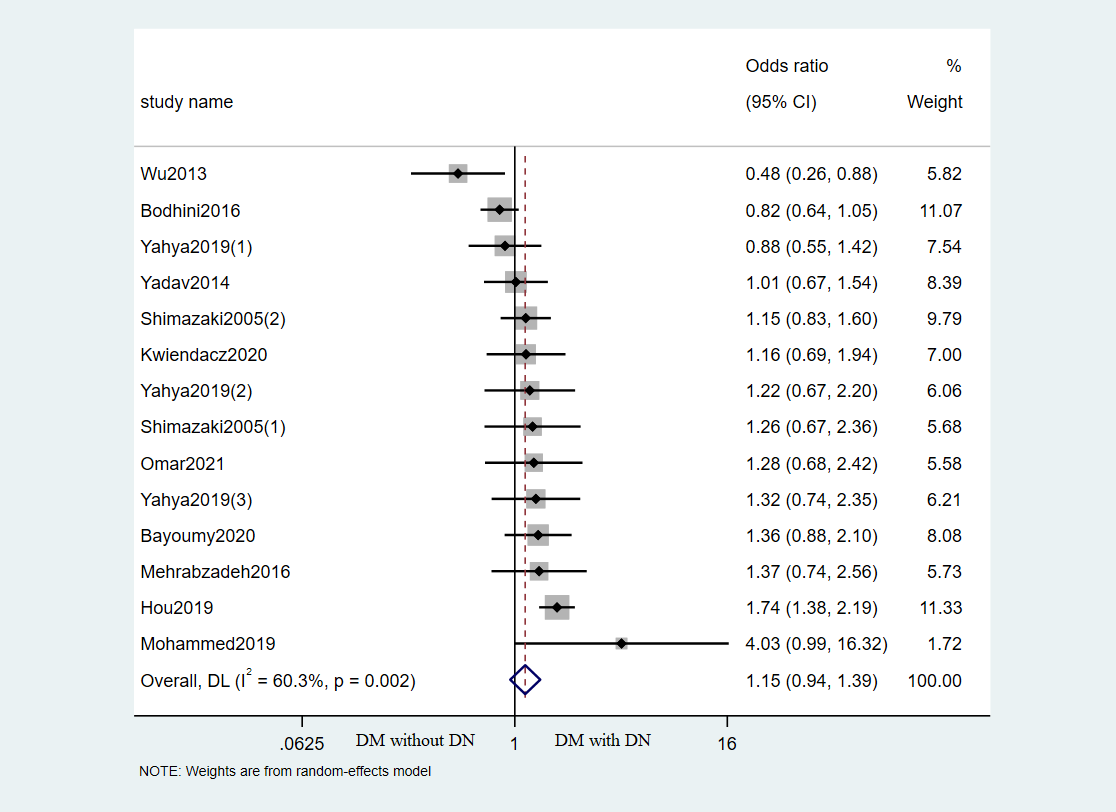


**Figure S11. Forest plot of the association between EMLO1 rs741301 polymorphism and DN risk under the codominant model in DN vs. DM patients**


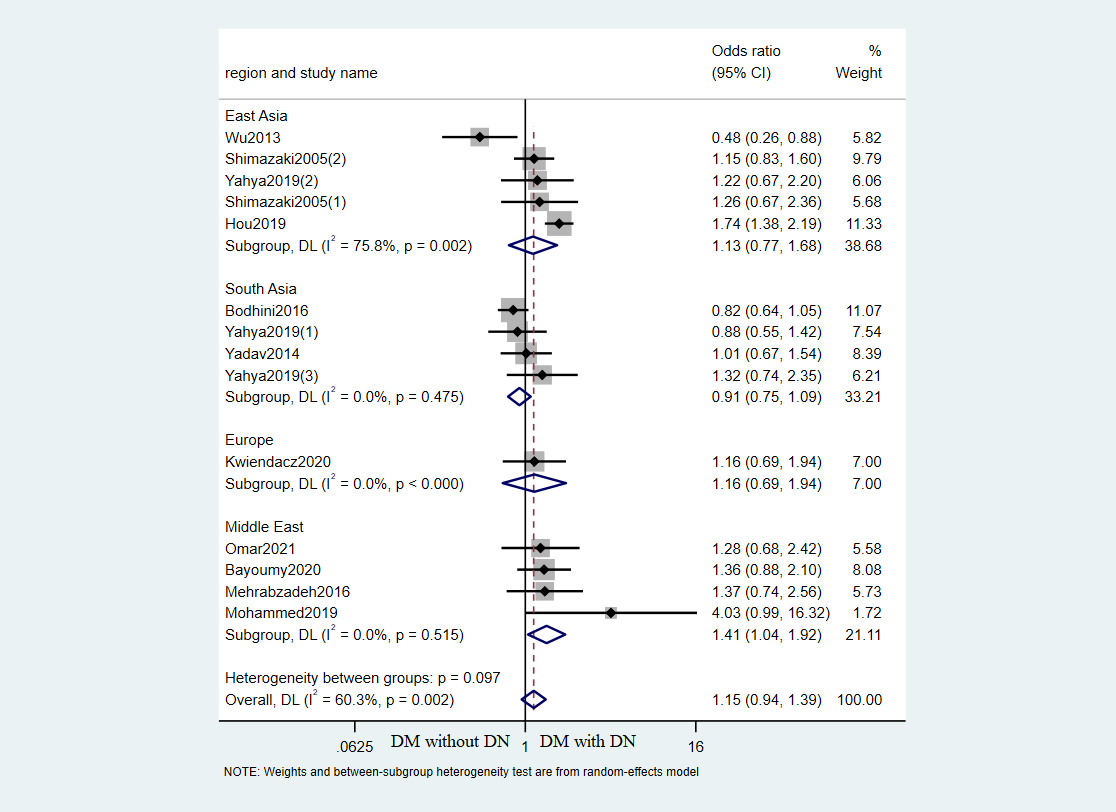


**Figure S12. Forest plot of the association between EMLO1 rs741301 polymorphism and DN risk by region stratification under the codominant model in DN vs. DM patients**


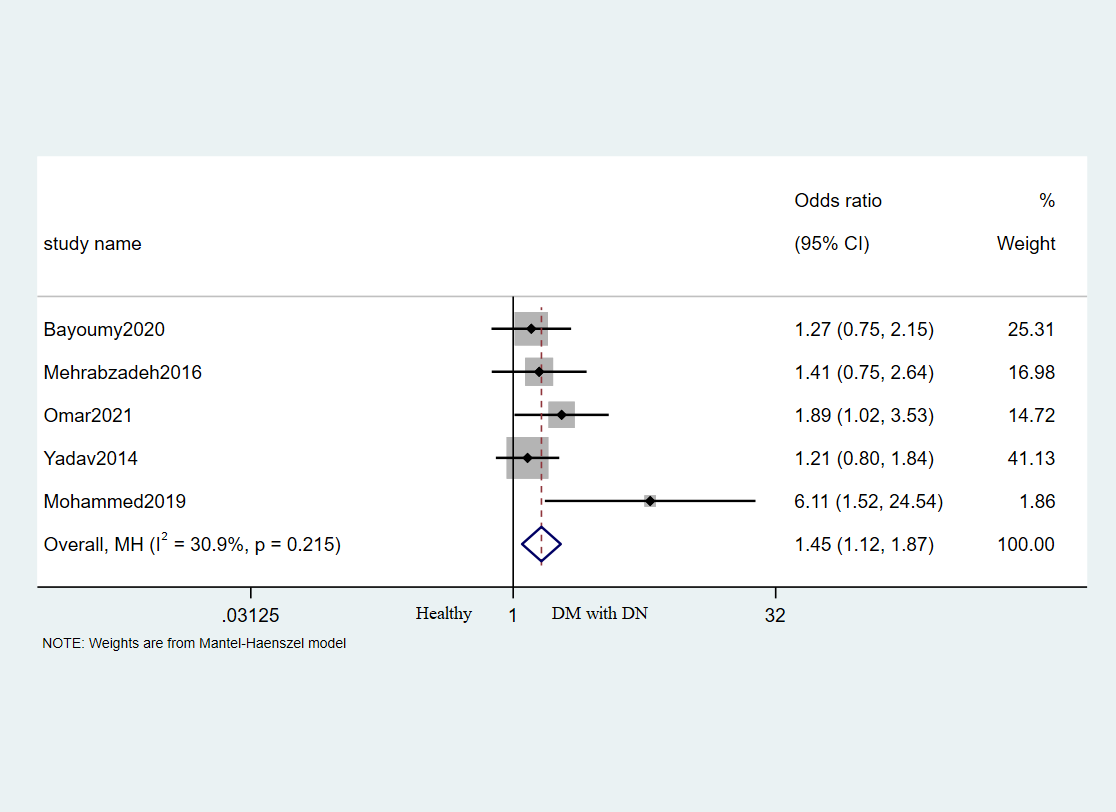


**Figure S13.** **Forest plot of the association between EMLO1 rs741301 polymorphism and DN risk under the codominant model in DN vs. healthy patients**


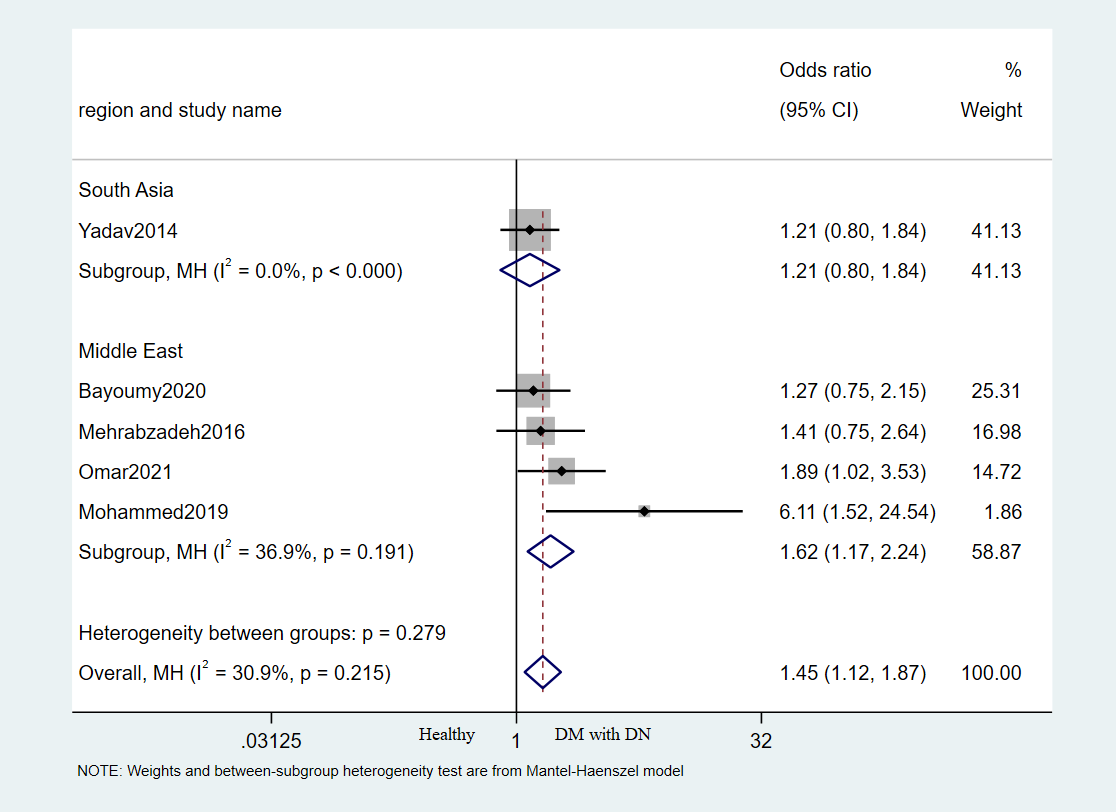


**Figure S14.** **Forest plot of the association between EMLO1 rs741301 polymorphism and DN risk by region stratification under the codominant model in DN vs. healthy patients**


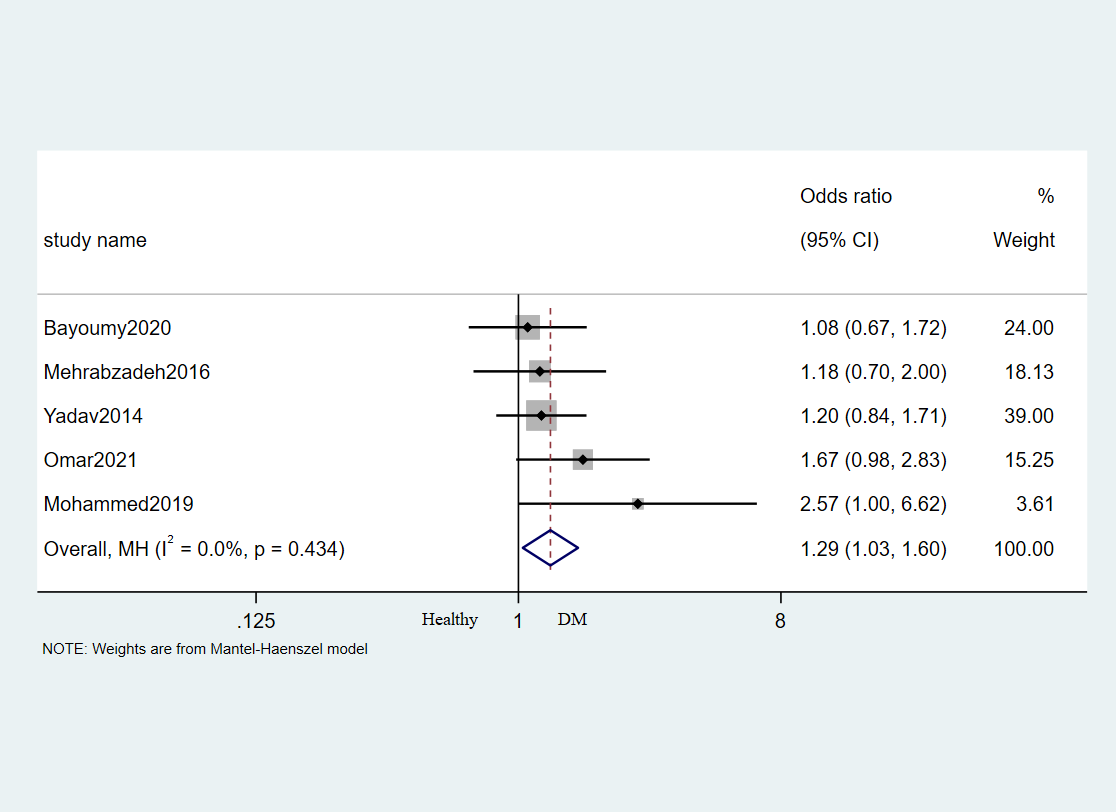


**Figure S15. Forest plot of the association between EMLO1 rs741301 polymorphism and DN risk under the codominant model in DM vs. healthy patients**


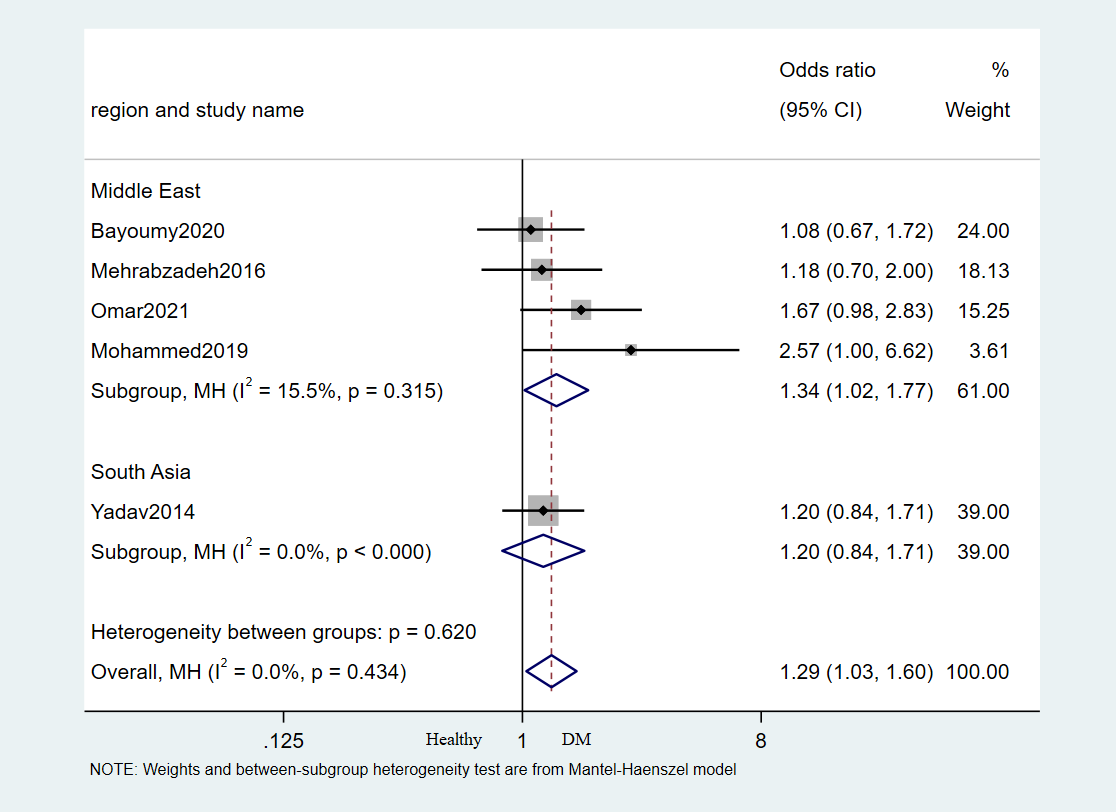


**Figure S16.** **Forest plot of the association between EMLO1 rs741301 polymorphism and DN risk by region stratification under the codominant model in DM vs. healthy patients**


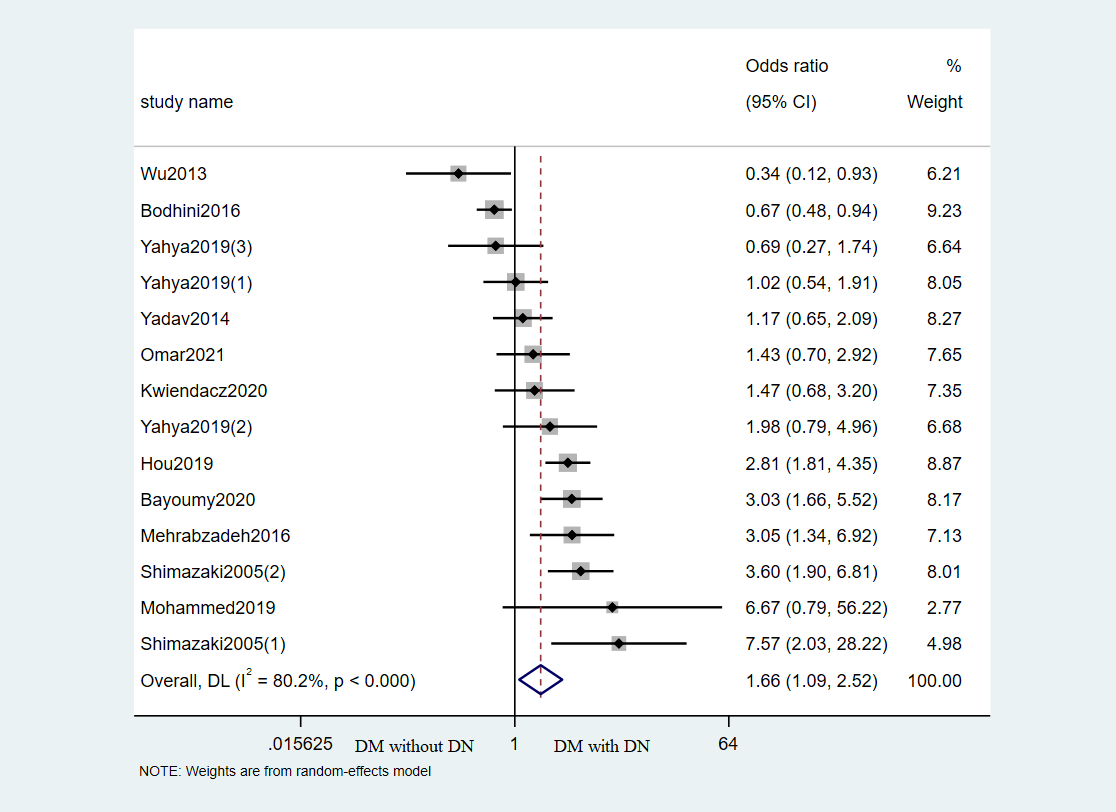


**Figure S17. Forest plot of the association between EMLO1 rs741301 polymorphism and DN risk under the homozygote model in DN vs. DM patients**


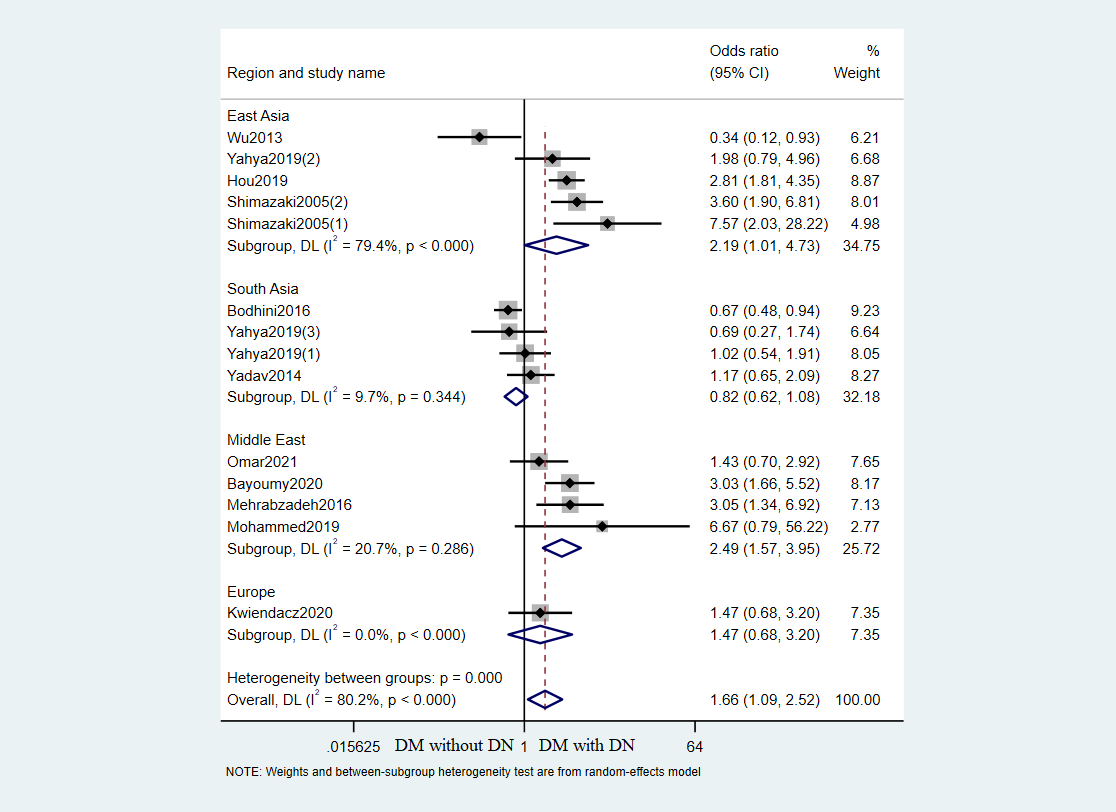


**Figure S18.** **Forest plot of the association between EMLO1 rs741301 polymorphism and DN risk by region stratification under the homozygote model in DN vs. DM patients**


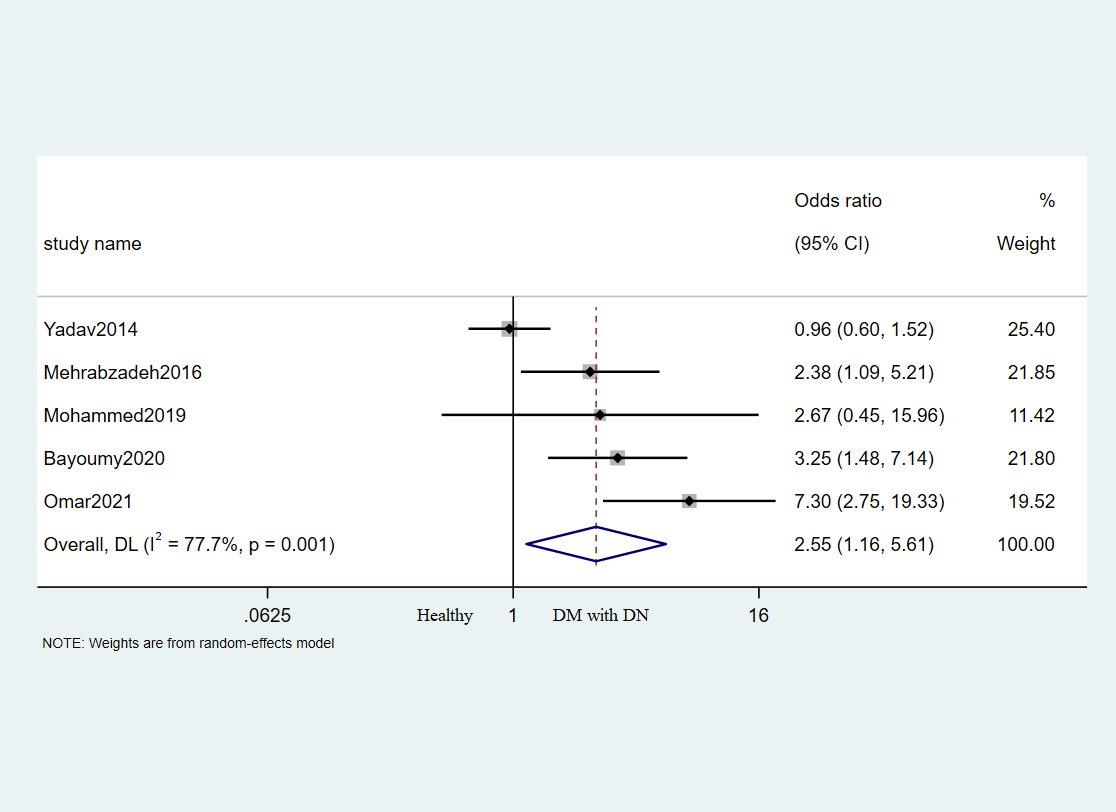


**Figure S19. Forest plot of the association between EMLO1 rs741301 polymorphism and DN risk under the homozygote model in DN vs. healthy patients**


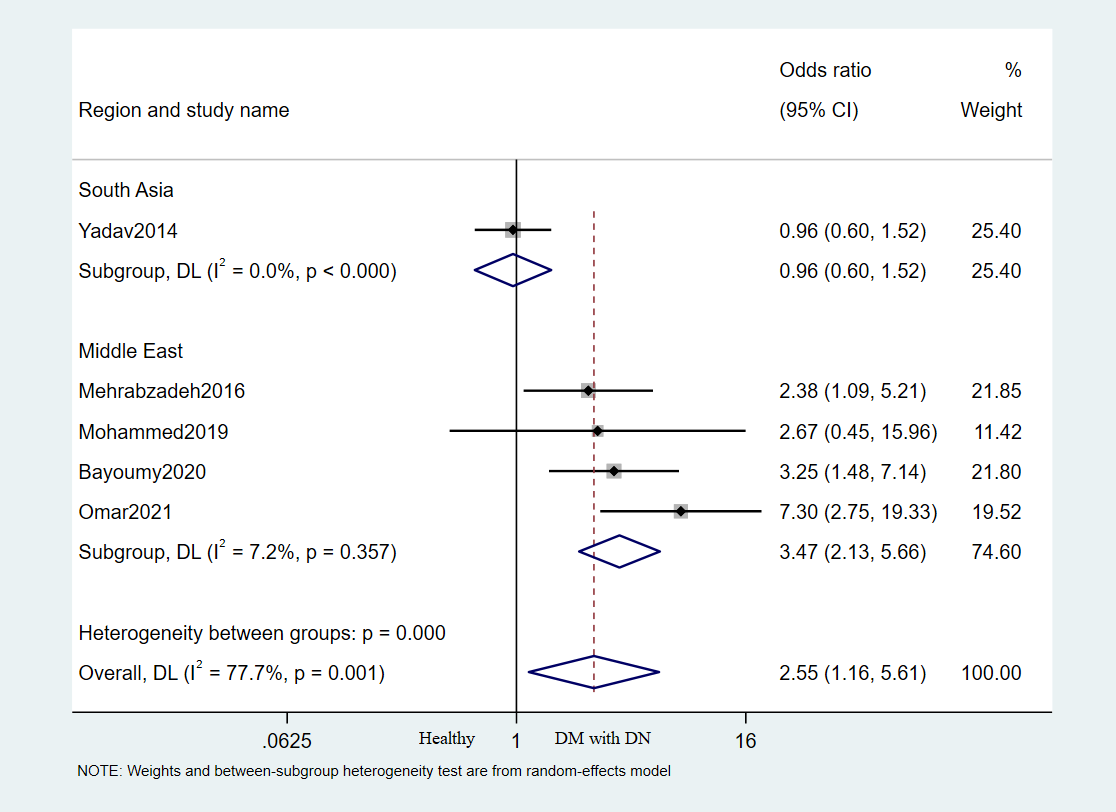


**Figure S20. Forest plot of the association between EMLO1 rs741301 polymorphism and DN risk by region stratification under the homozygote model in DN vs. healthy patients**


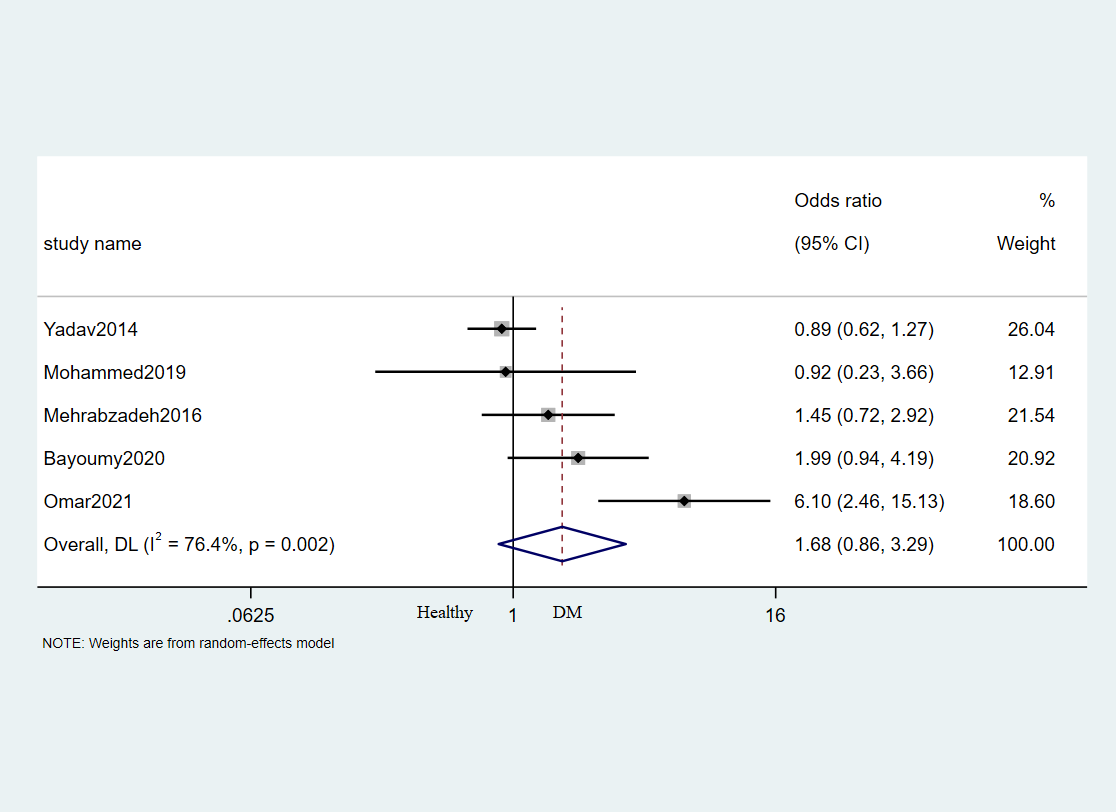


**Figure S21.** **Forest plot of the association between EMLO1 rs741301 polymorphism and DN risk under the homozygote model in DM vs. healthy patients**


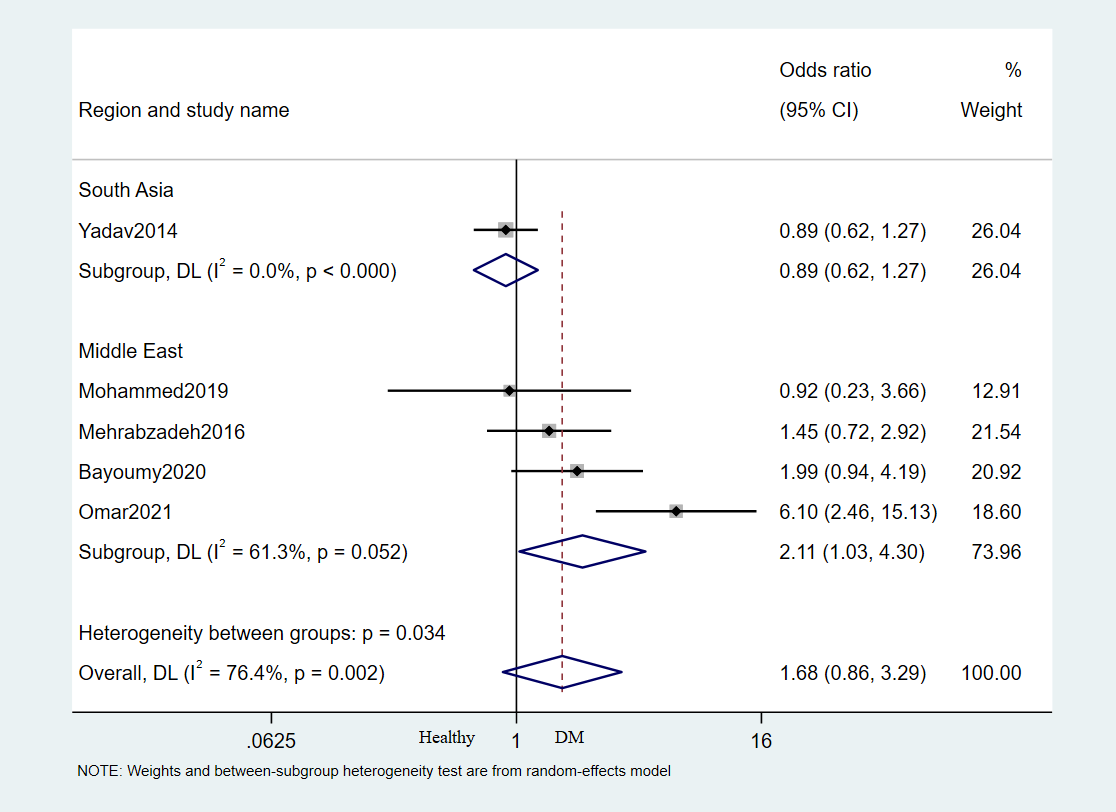


**Figure S22.** **Forest plot of the association between EMLO1 rs741301 polymorphism and DN risk by region stratification under the homozygote model in DM vs. healthy patients**


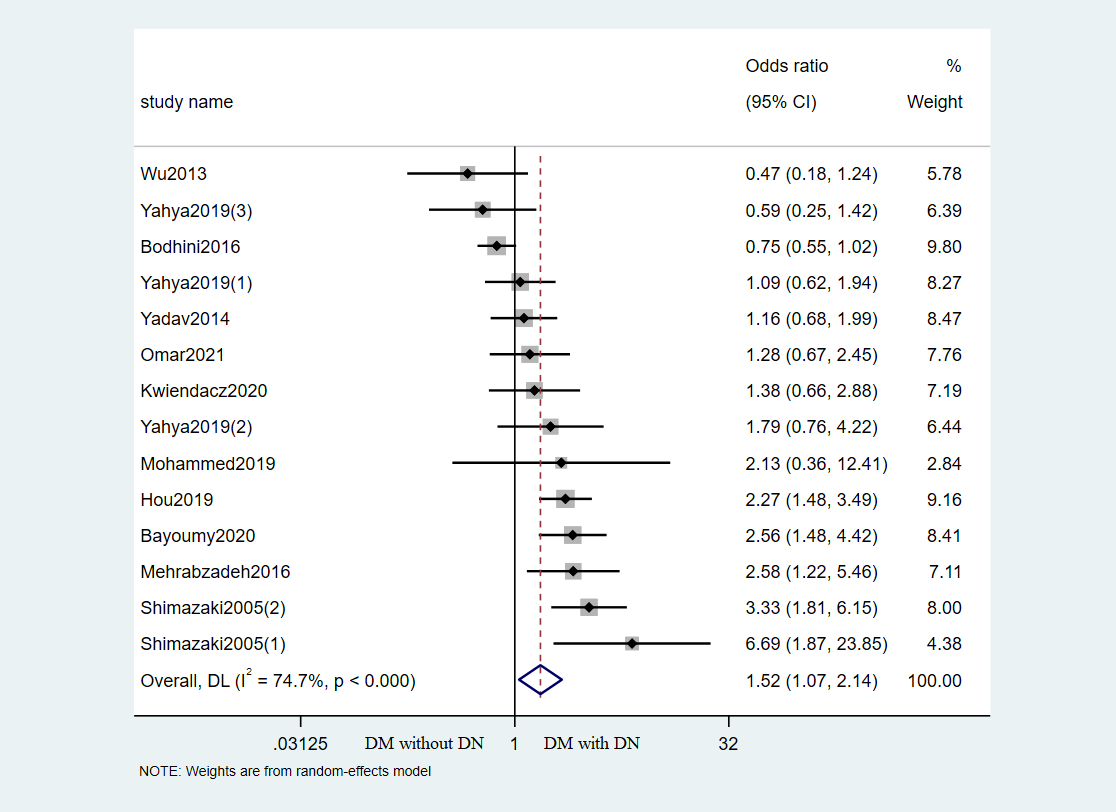


**Figure S23. Forest plot of the association between EMLO1 rs741301 polymorphism and DN risk under the recessive model in DN vs. DM patients**


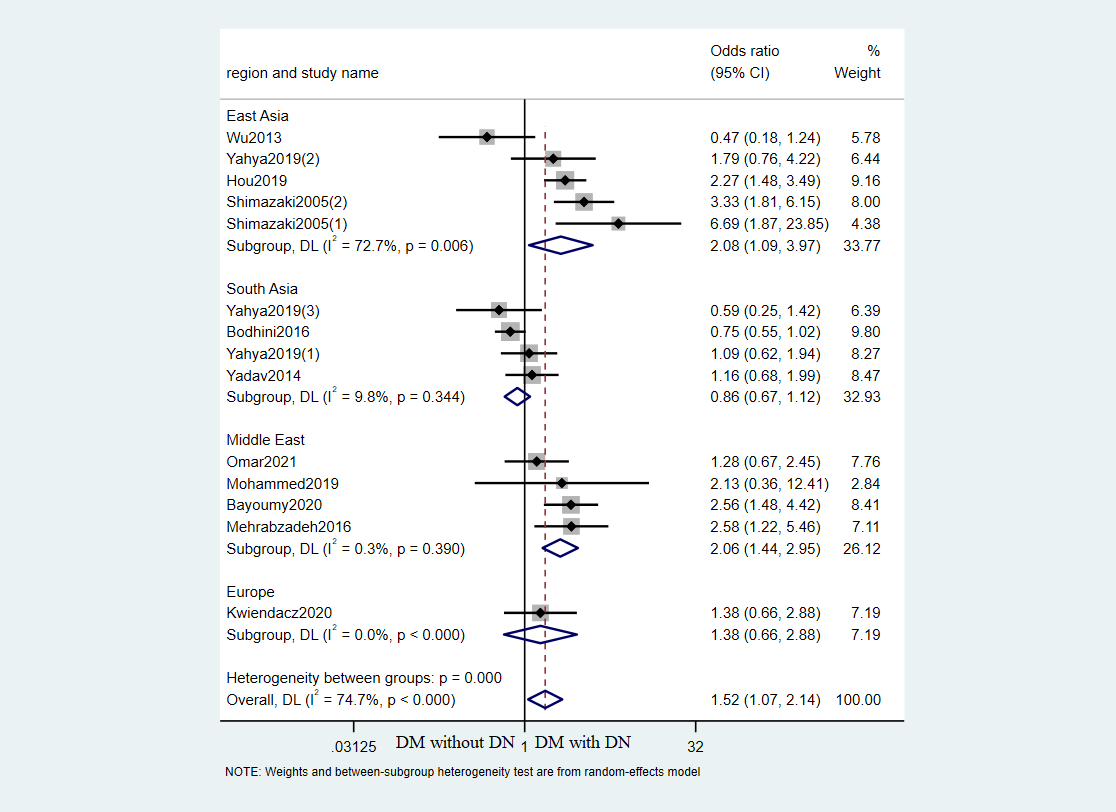


**Figure S24.** **Forest plot of the association between EMLO1 rs741301 polymorphism and DN risk by region stratification under the recessive model in DN vs. DM patients**


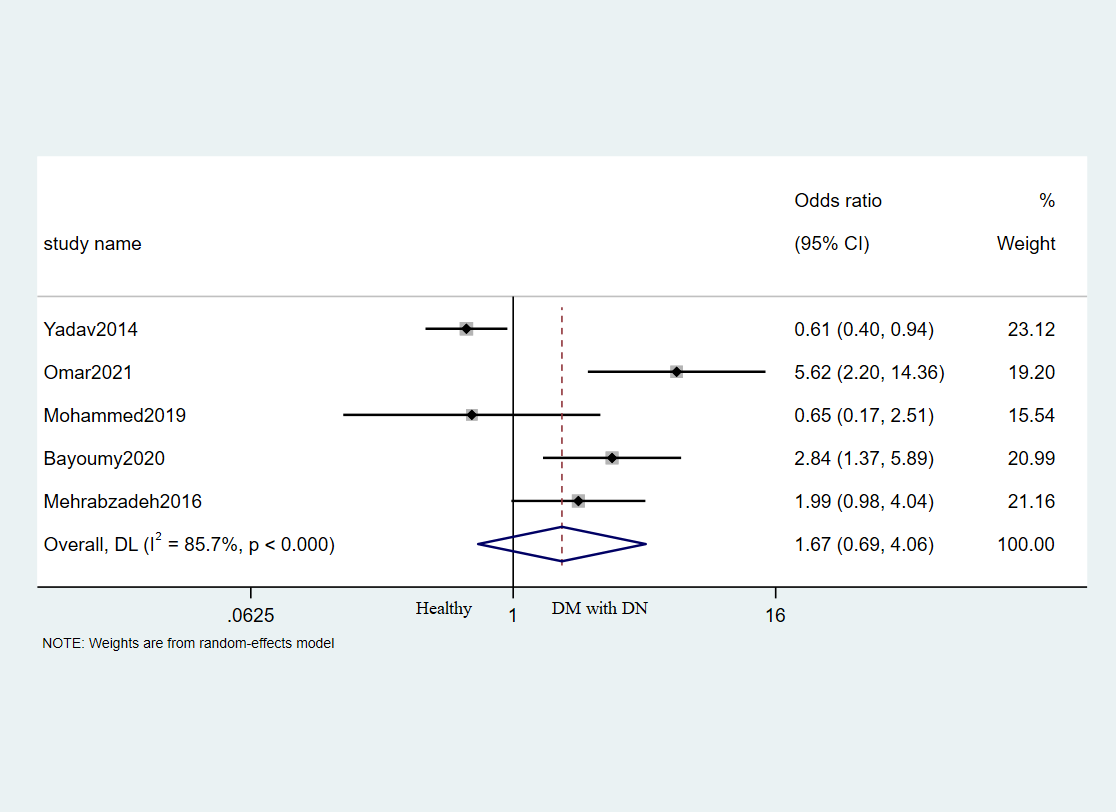


**Figure S25.** **Forest plot of the association between EMLO1 rs741301 polymorphism and DN risk under the recessive model in DN vs. healthy patients**


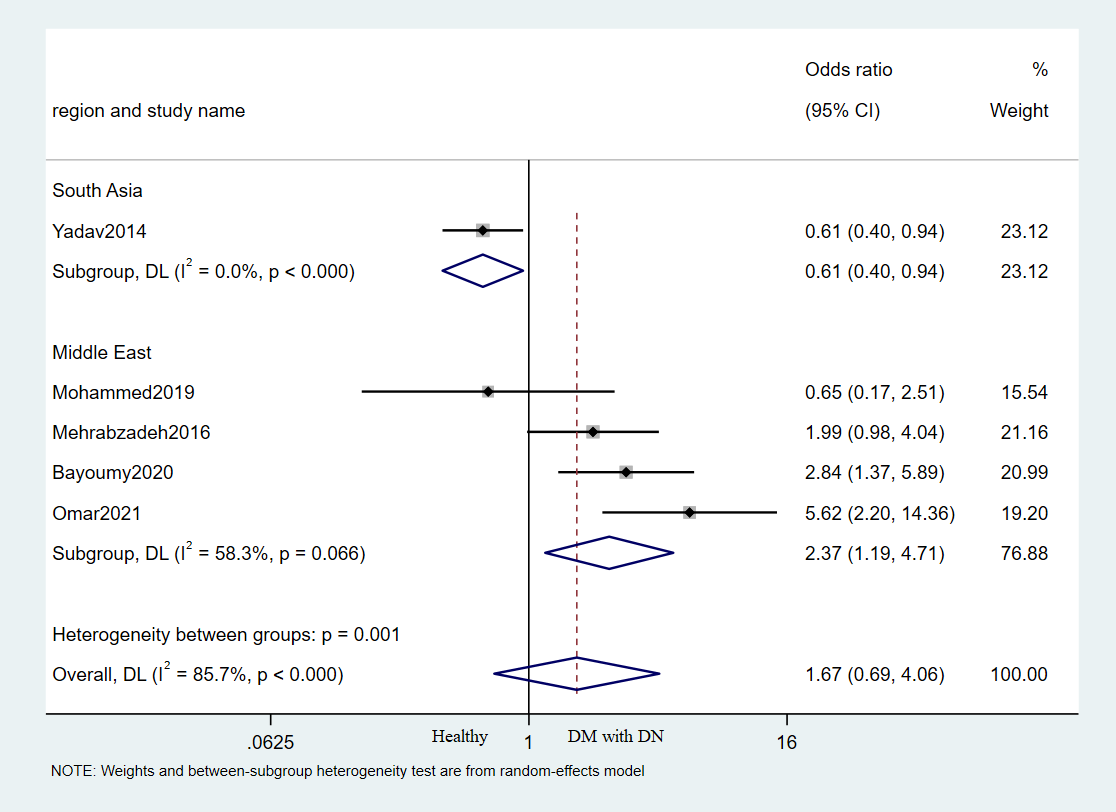


**Figure S26. Forest plot of the association between EMLO1 rs741301 polymorphism and DN risk by region stratification under the recessive model in DN vs. healthy patients**


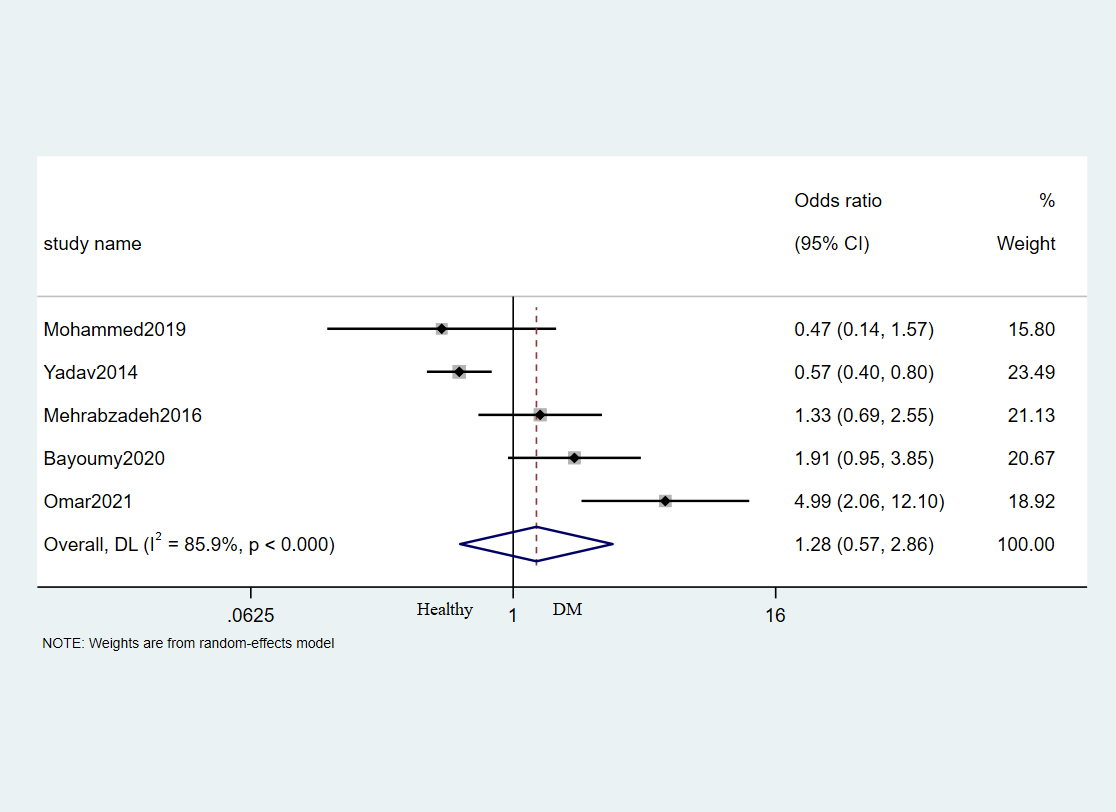


**Figure S27. Forest plot of the association between EMLO1 rs741301 polymorphism and DN risk under the recessive model in DM vs. healthy patients**


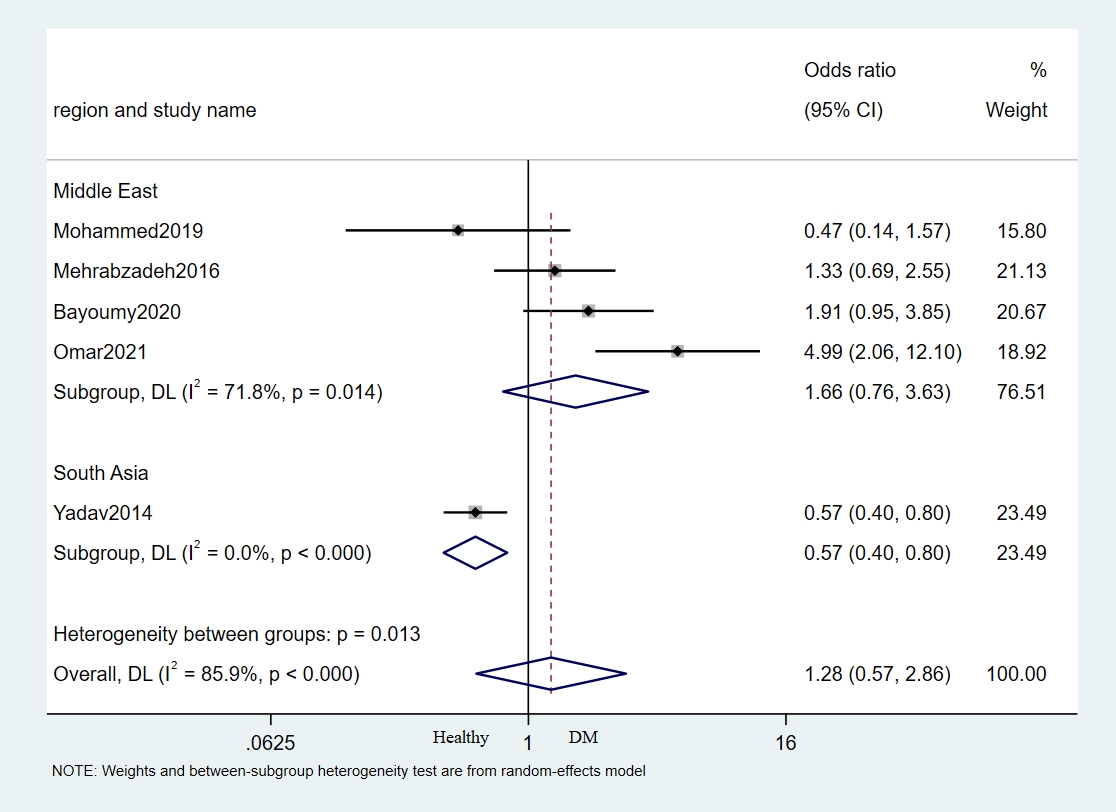


**Figure S28. Forest plot of the association between EMLO1 rs741301 polymorphism and DN risk by region stratification under the recessive model in DM vs. healthy patients**


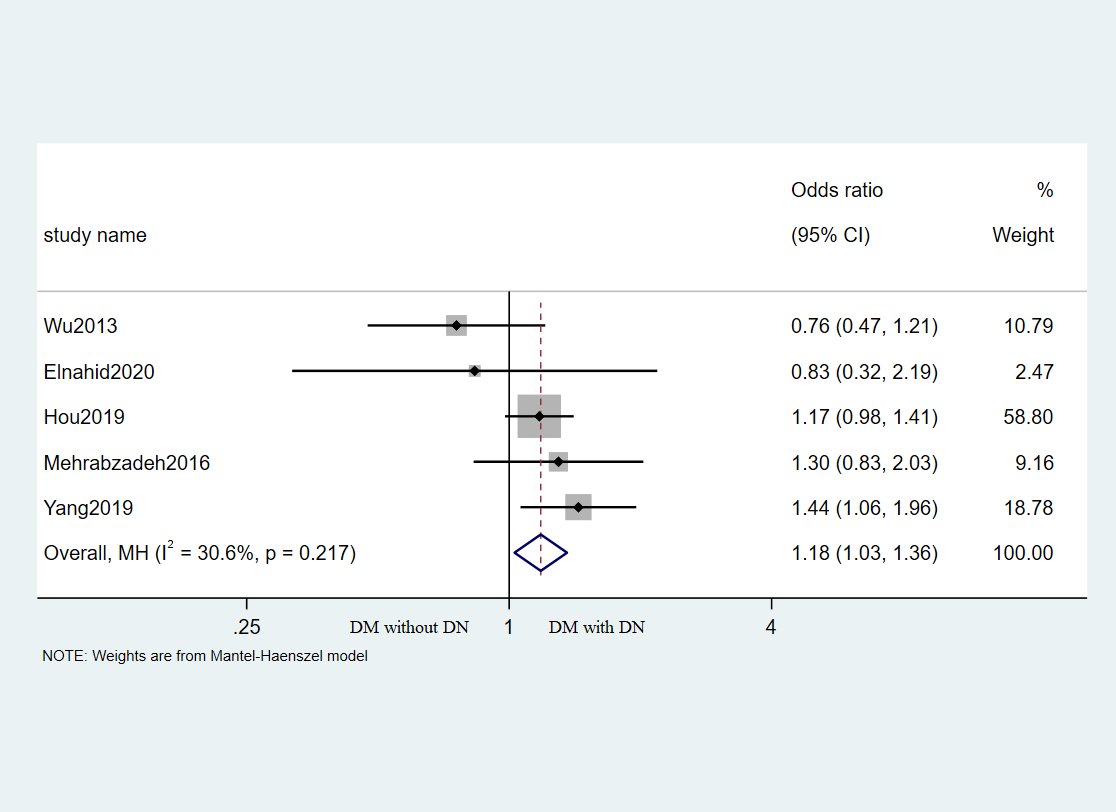


**Figure S29. Forest plot of the association between EMLO1 rs1345365 polymorphism and DN risk under the allele model in DN vs. DM patients**


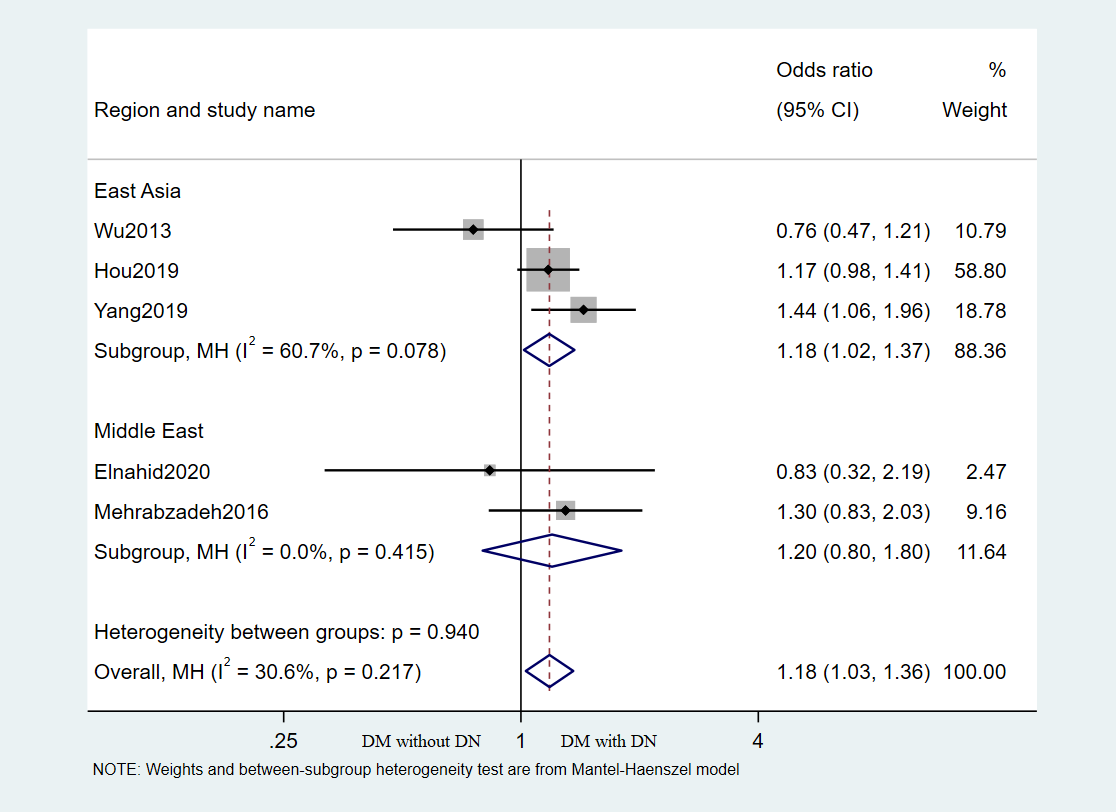


**Figure S30. Forest plot of the association between EMLO1 rs1345365 polymorphism and DN risk by region stratification under the allele model in DN vs. DM patients**


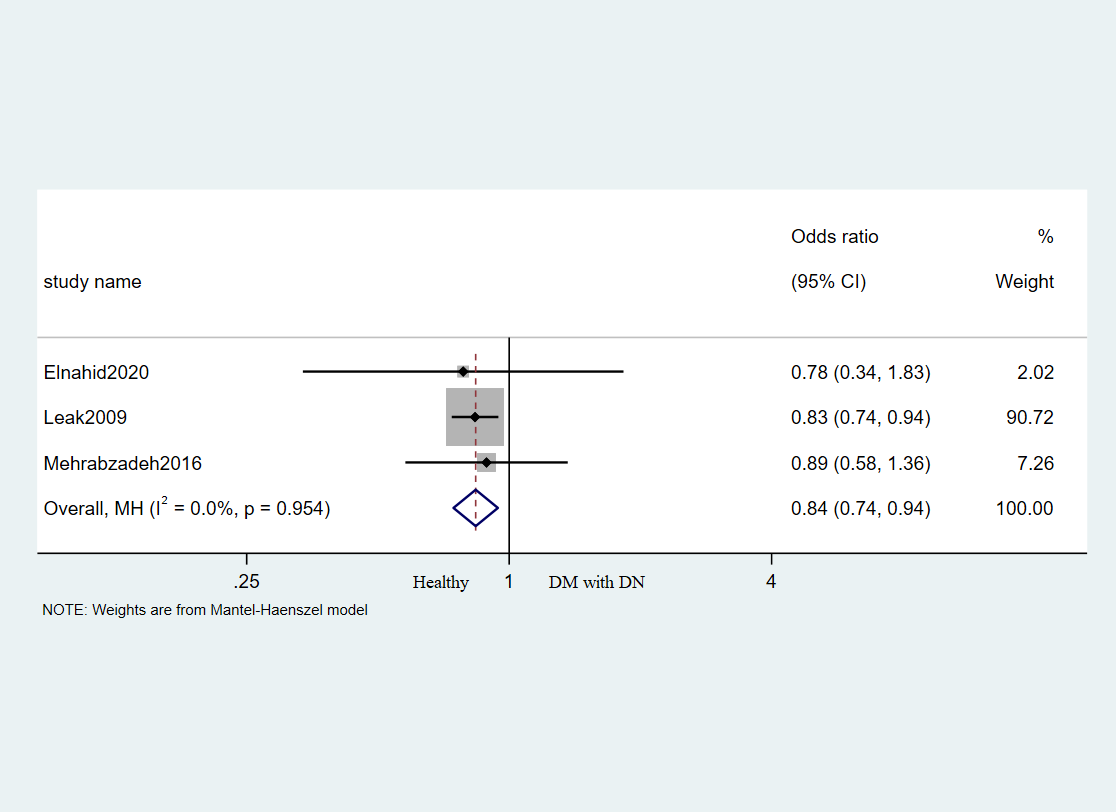


**Figure S31. Forest plot of the association between EMLO1 rs1345365 polymorphism and DN risk under the allele model in DN vs. healthy patients**


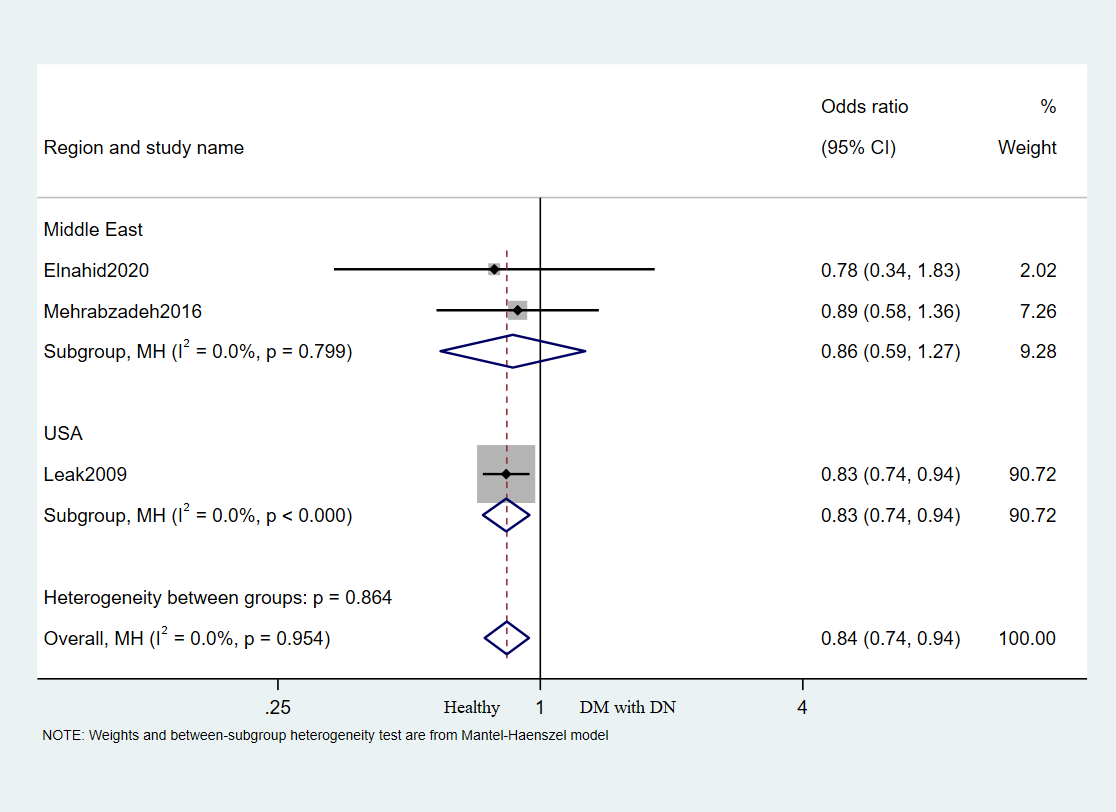


**Figure S32. Forest plot of the association between EMLO1 rs1345365 polymorphism and DN risk by region stratification under the allele model in DN vs. healthy patients**


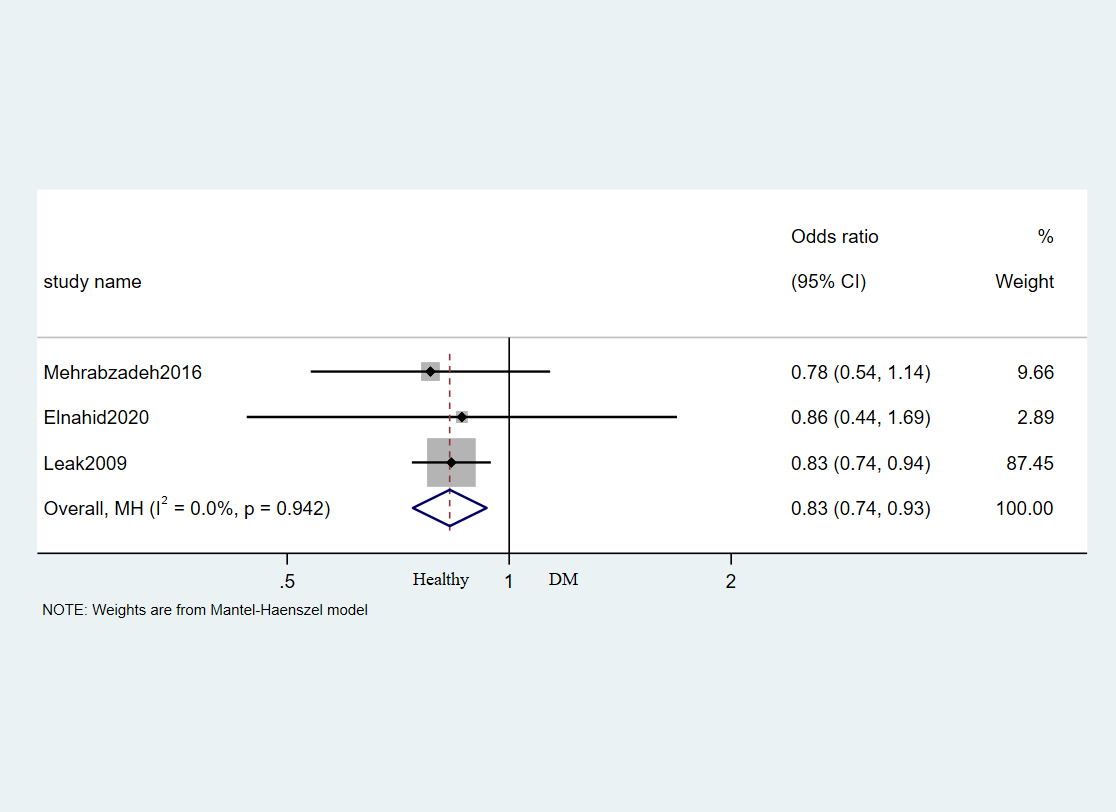


**Figure S33. Forest plot of the association between EMLO1 rs1345365 polymorphism and DN risk under the allele model in DM vs. healthy patients**


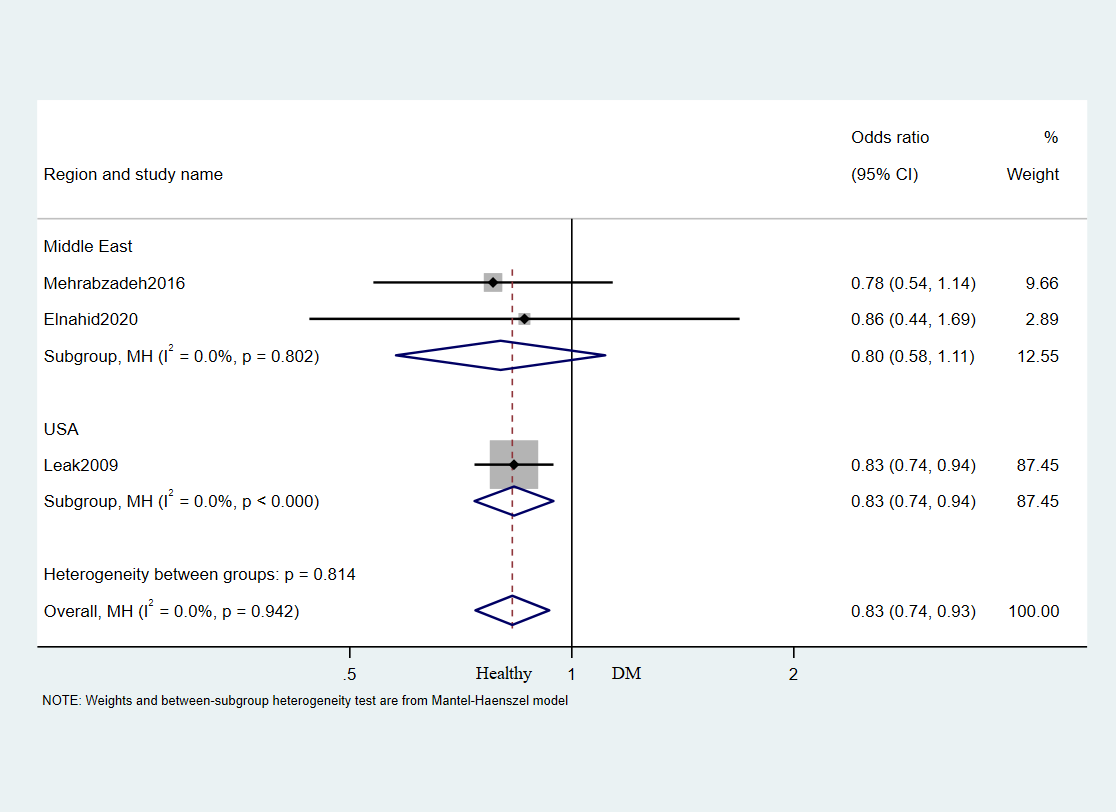


**Figure S34. Forest plot of the association between EMLO1 rs1345365 polymorphism and DN risk by region stratification under the allele model in DM vs. healthy patients**


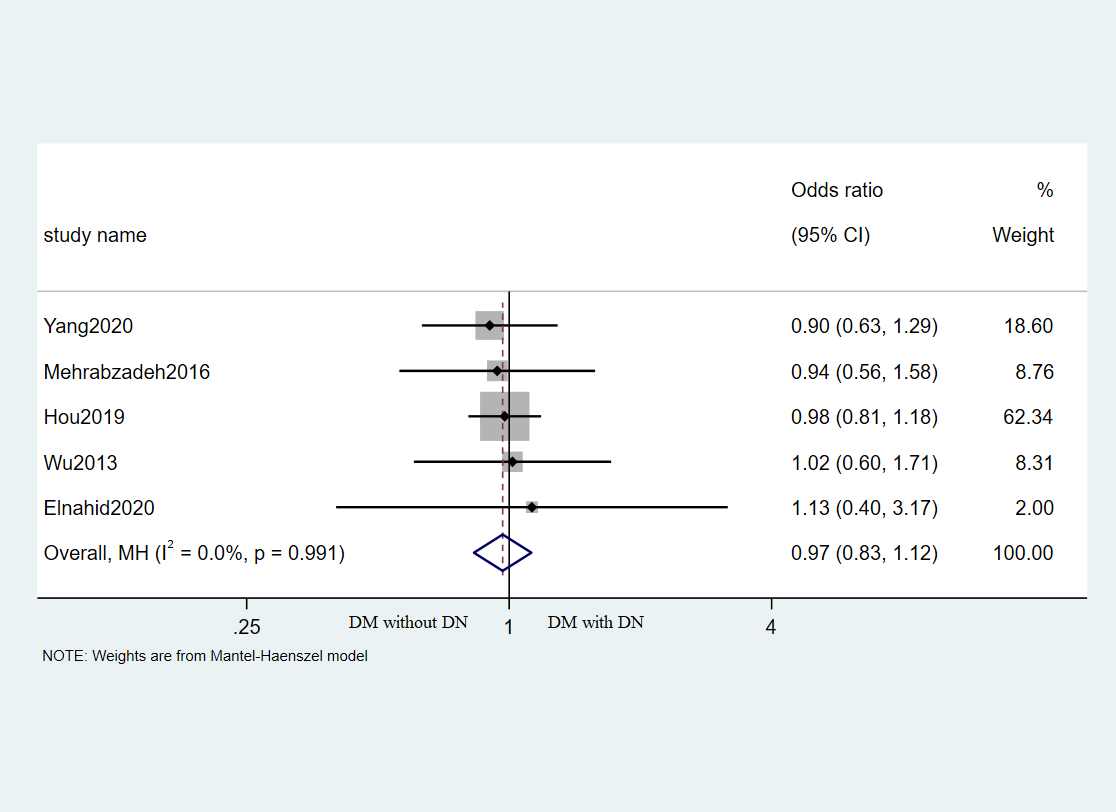


**Figure S35. Forest plot of the association between EMLO1 rs1345365 polymorphism and DN risk under the dominant model in DN vs. DM patients**


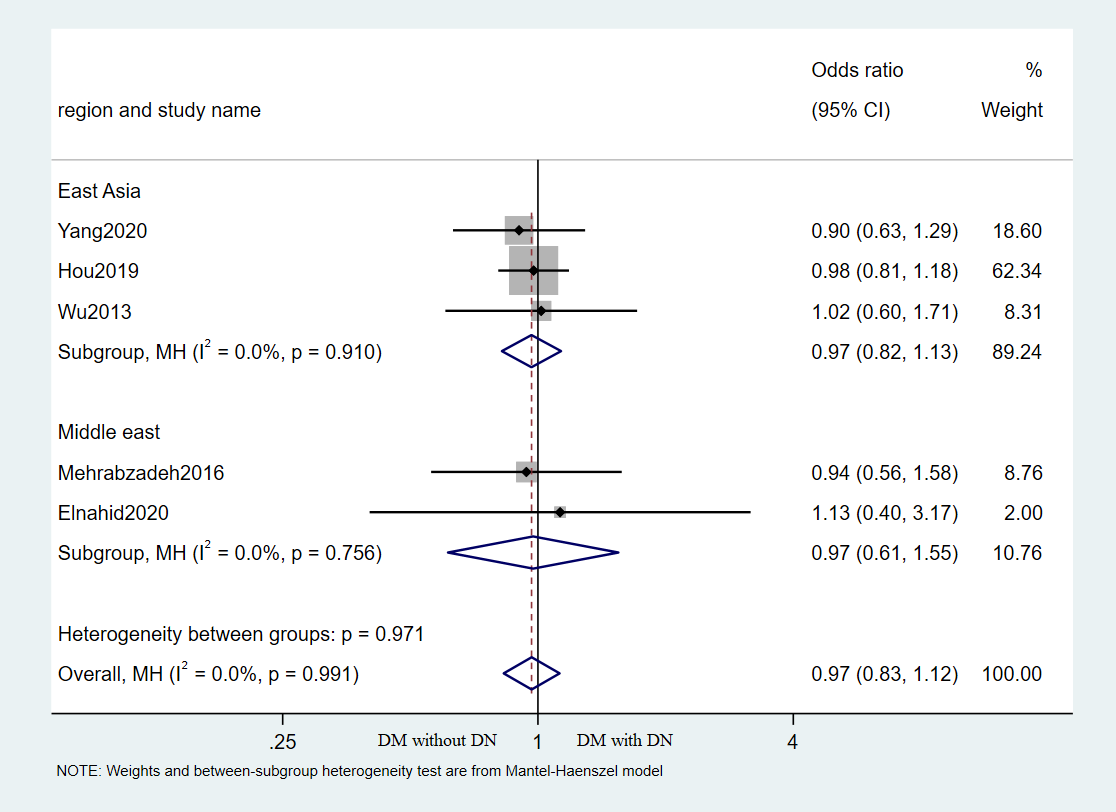


**Figure S36.** **Forest plot of the association between EMLO1 rs1345365 polymorphism and DN risk by region stratification under the dominant model in DN vs. DM patients**


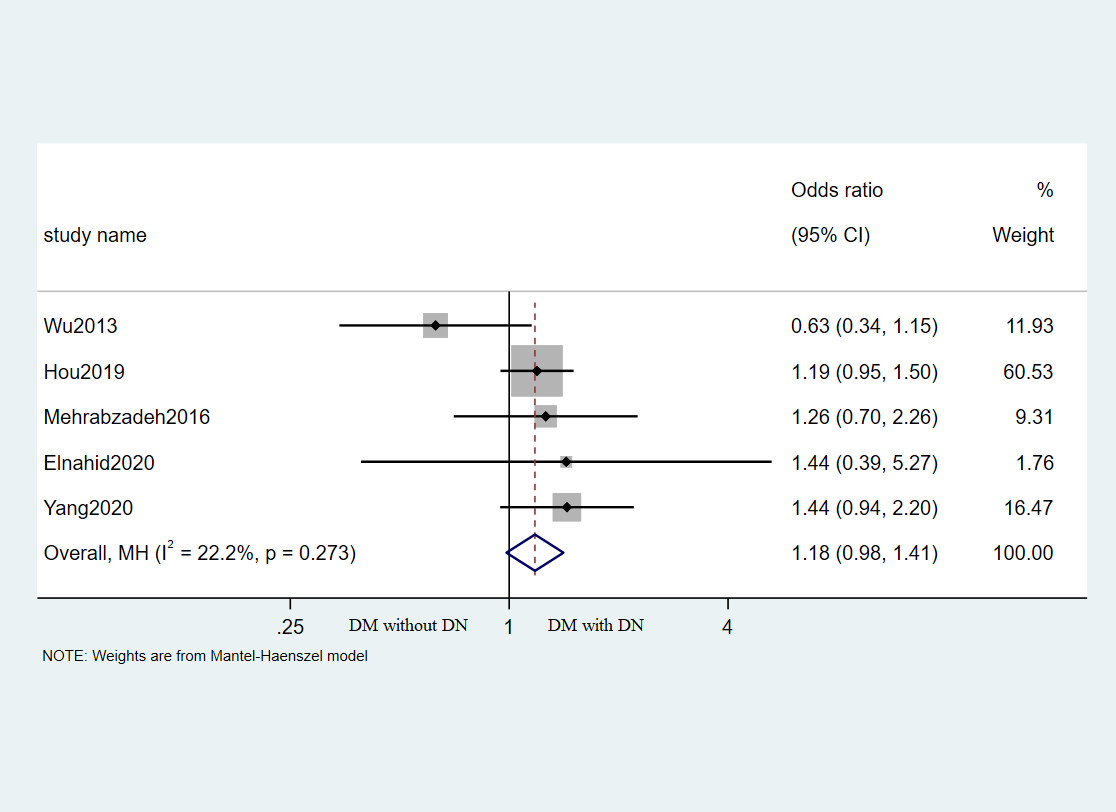


**Figure 37. Forest plot of the association between EMLO1 rs1345365 polymorphism and DN risk under the codominant model in DN vs. DM patients**


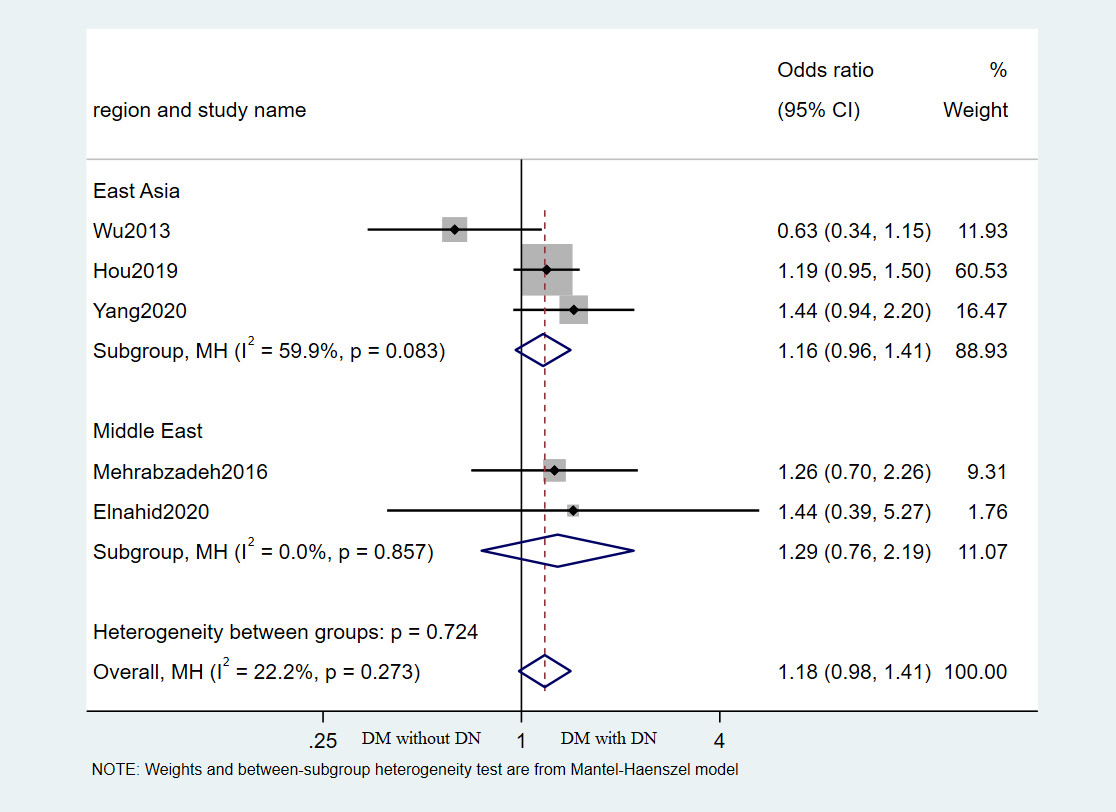


**Figure S38.** **Forest plot of the association between EMLO1 rs1345365 polymorphism and DN risk by region stratification under the codominant model in DN vs. DM patients**


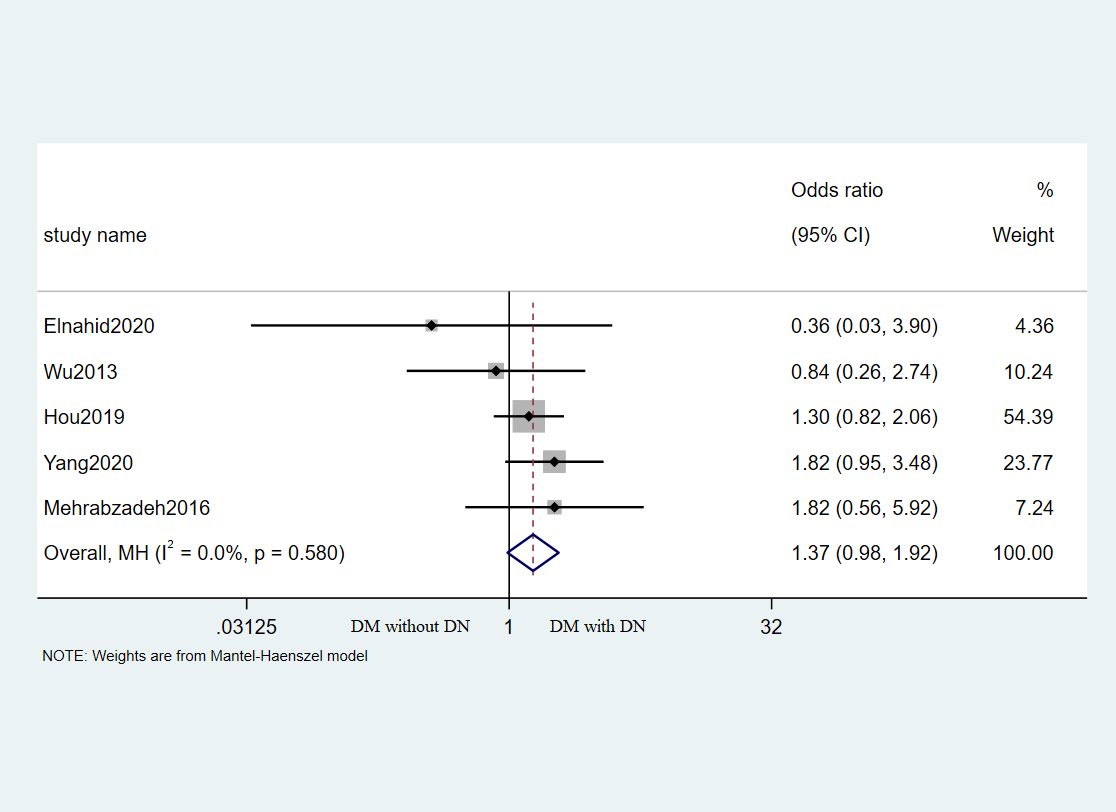


**Figure S39. Forest plot of the association between EMLO1 rs1345365 polymorphism and DN risk under the homozygote model in DN vs. DM patients**


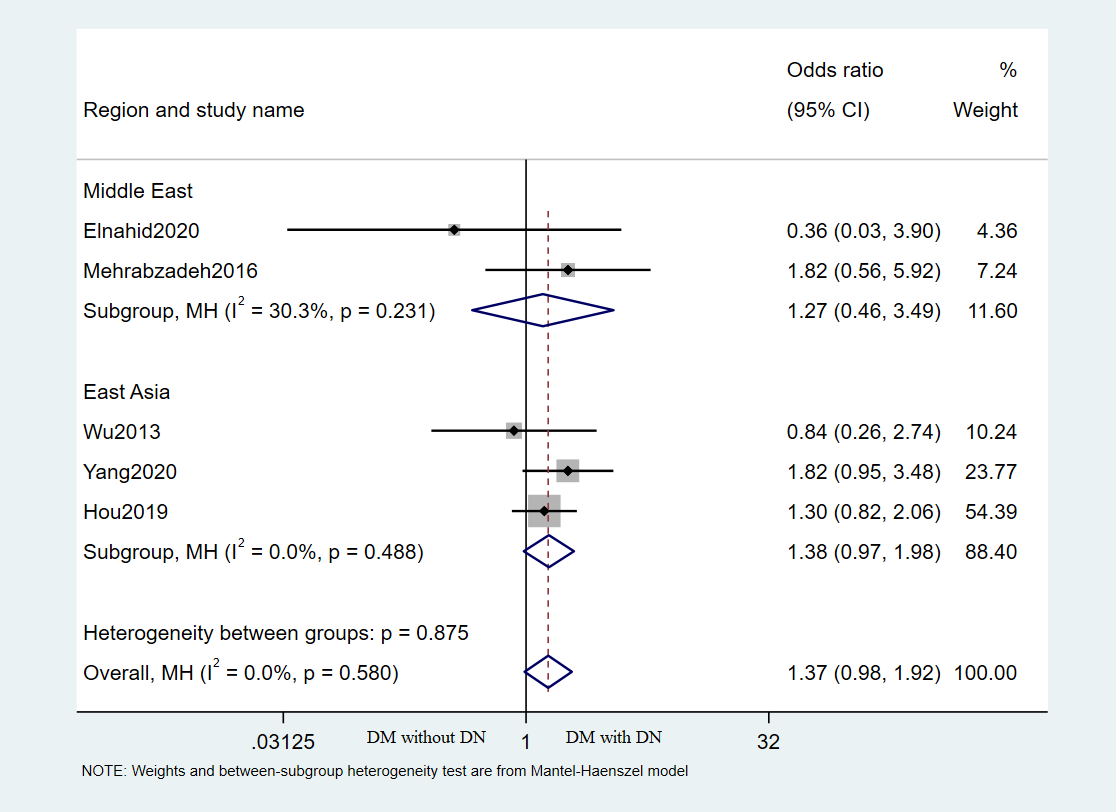


**Figure S40.** **Forest plot of the association between EMLO1 rs1345365 polymorphism and DN risk by region stratification under the homozygote model in DN vs. DM patients**


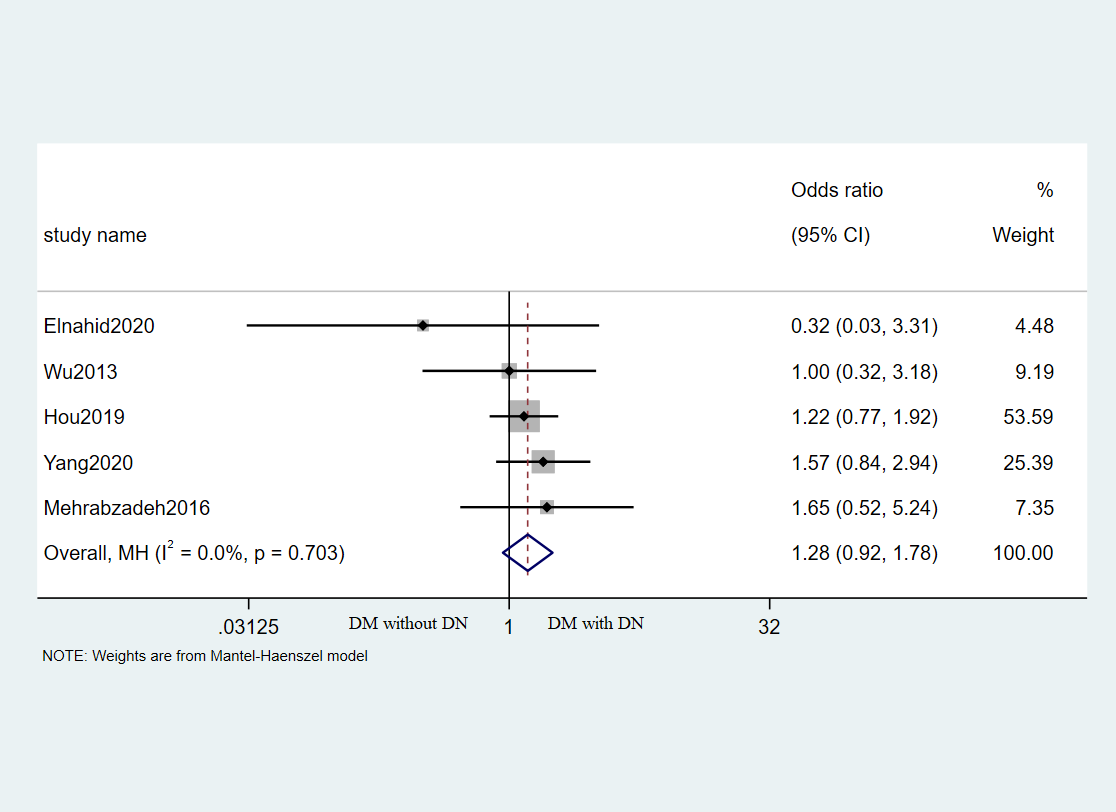


**Figure S41. Forest plot of the association between EMLO1 rs1345365 polymorphism and DN risk under the recessive model in DN vs. DM patients**


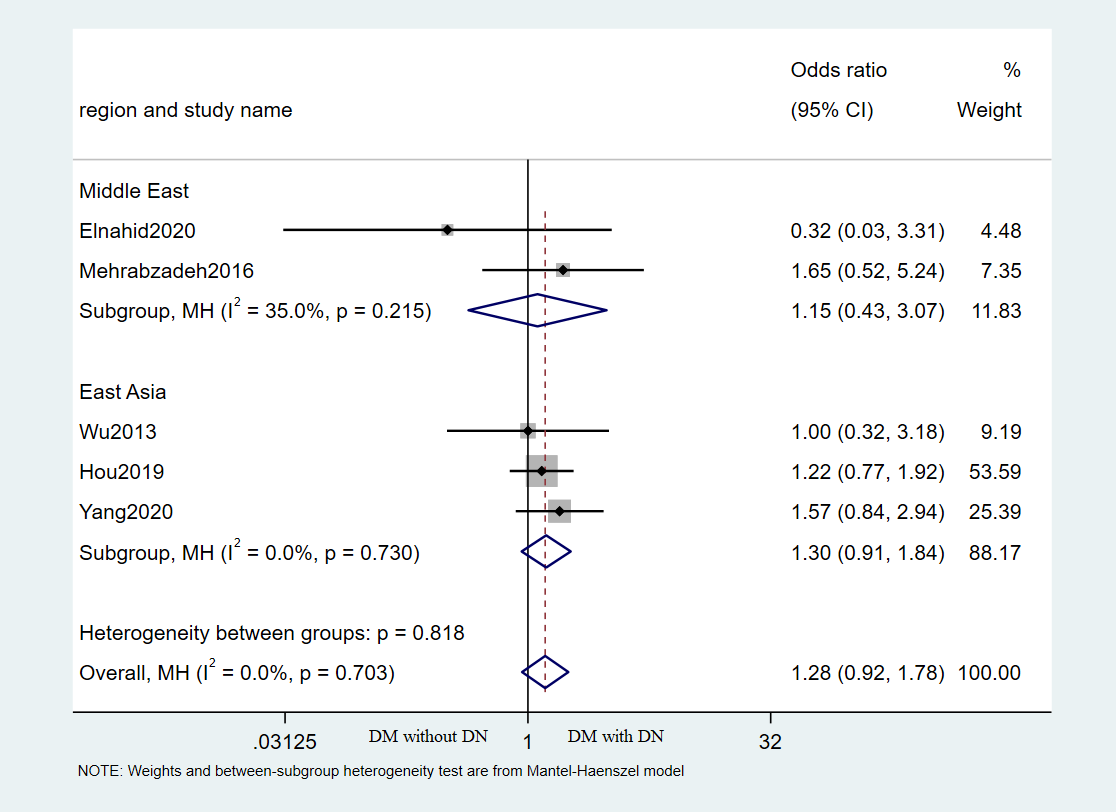


**Figure S42. Forest plot of the association between EMLO1 rs1345365 polymorphism and DN risk by region stratification under the recessive model in DN vs. DM patients**


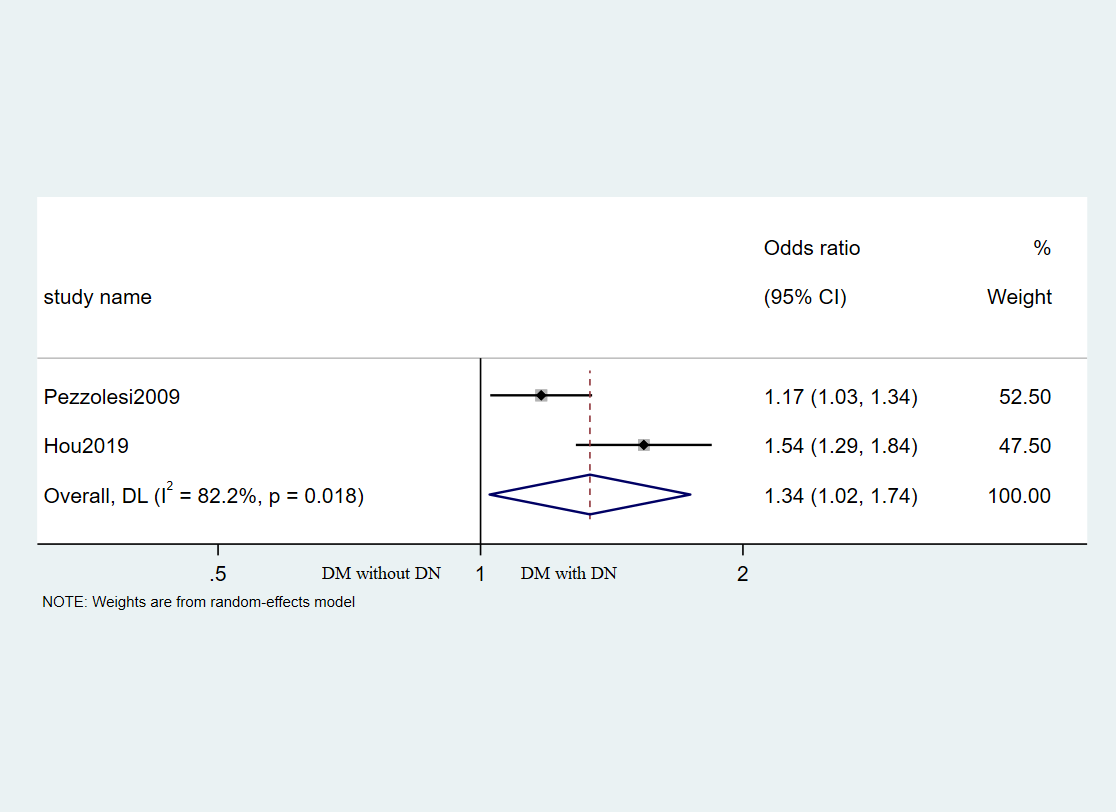


**Figure S43.** **Forest plot of the association between EMLO1 rs10255208 polymorphism and DN risk under the allele model in DN vs. DM patients**


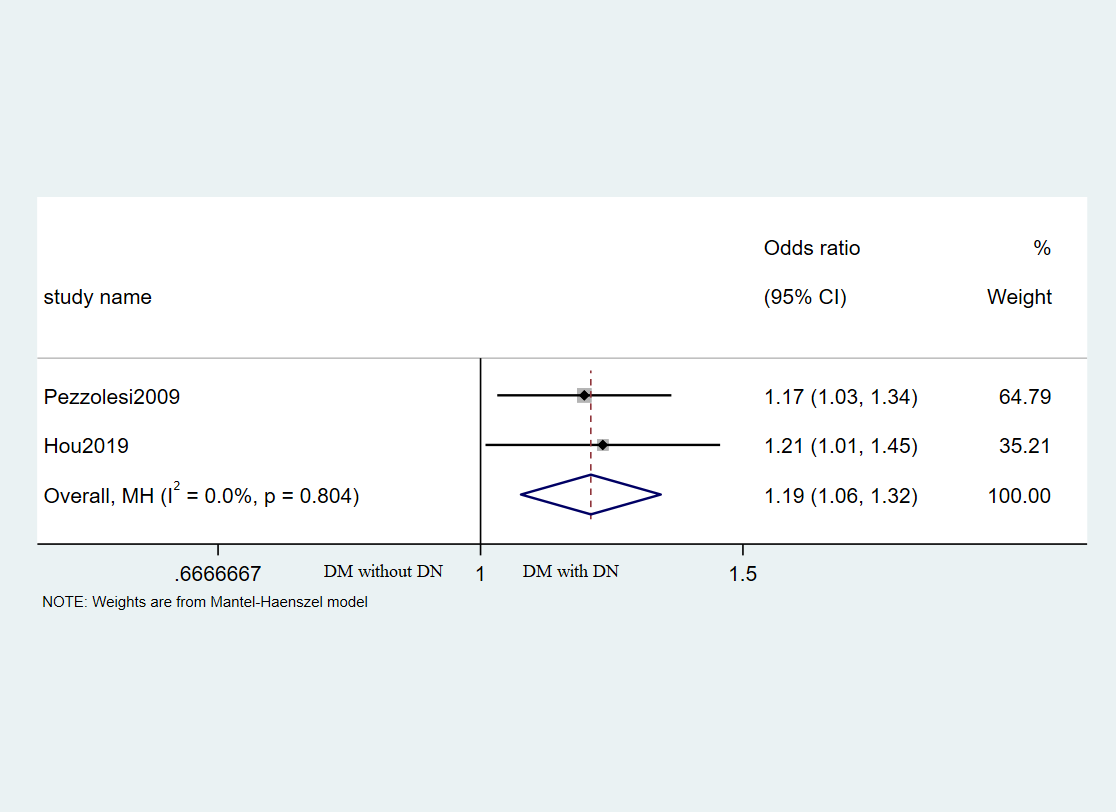


**Figure S44. Forest plot of the association between EMLO1 rs7782979 polymorphism and DN risk under the allele model in DN vs. DM patients**


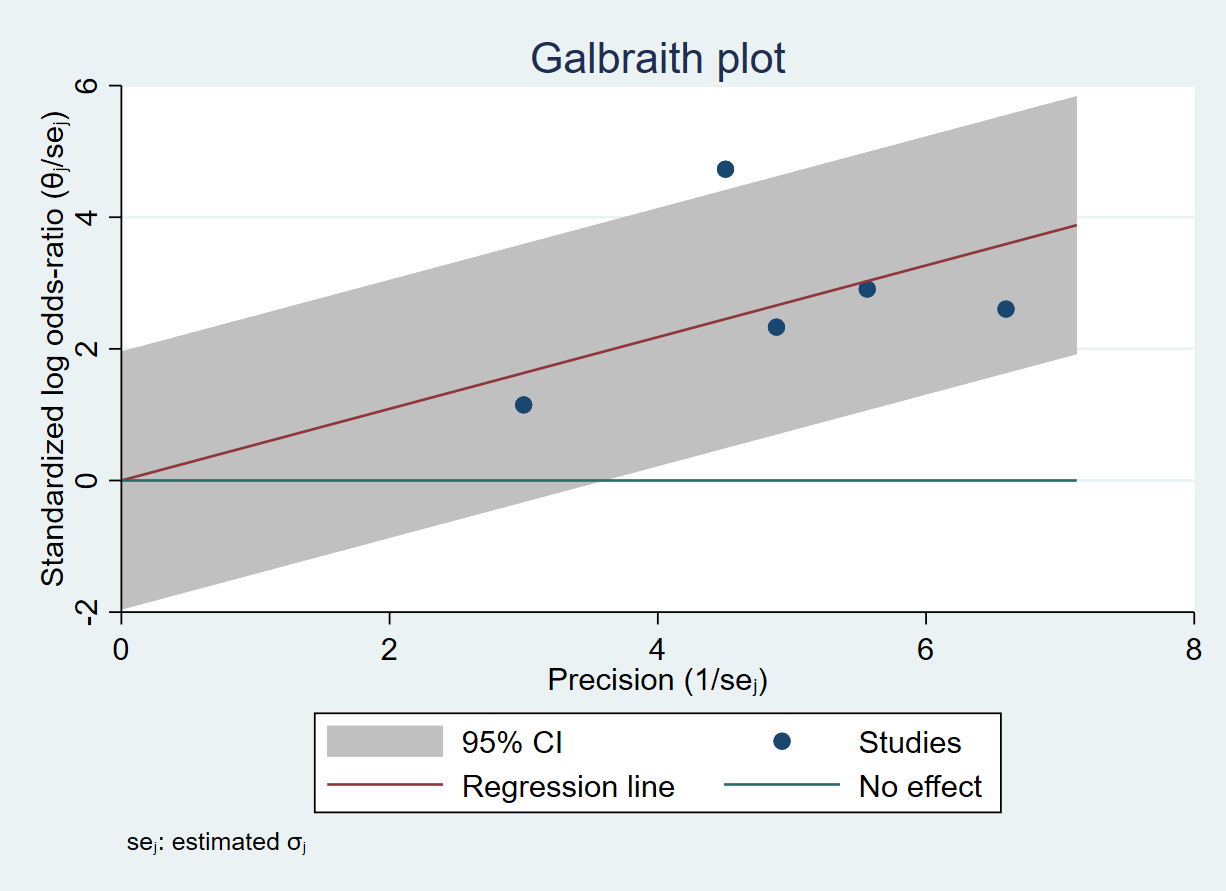


**Figure S45.** **Galbraith plot used to address heterogeneity of** **EMLO1 rs741301 polymorphism and DN risk under the allele model in DN vs. healthy patients**


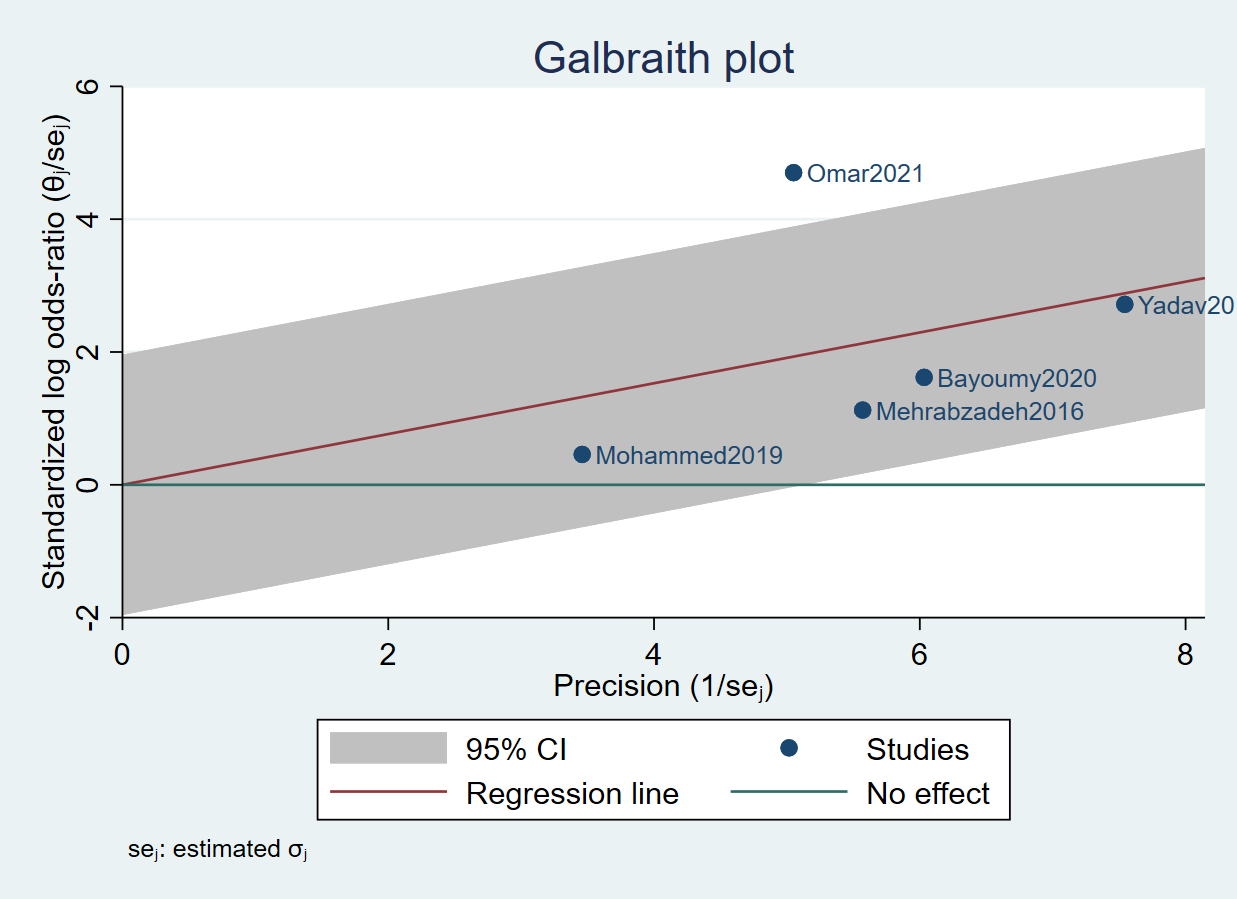


**Figure S46.** **Galbraith plot used to address heterogeneity of** **EMLO1 rs741301 polymorphism and DN risk under the allele model in DM vs. healthy patients**


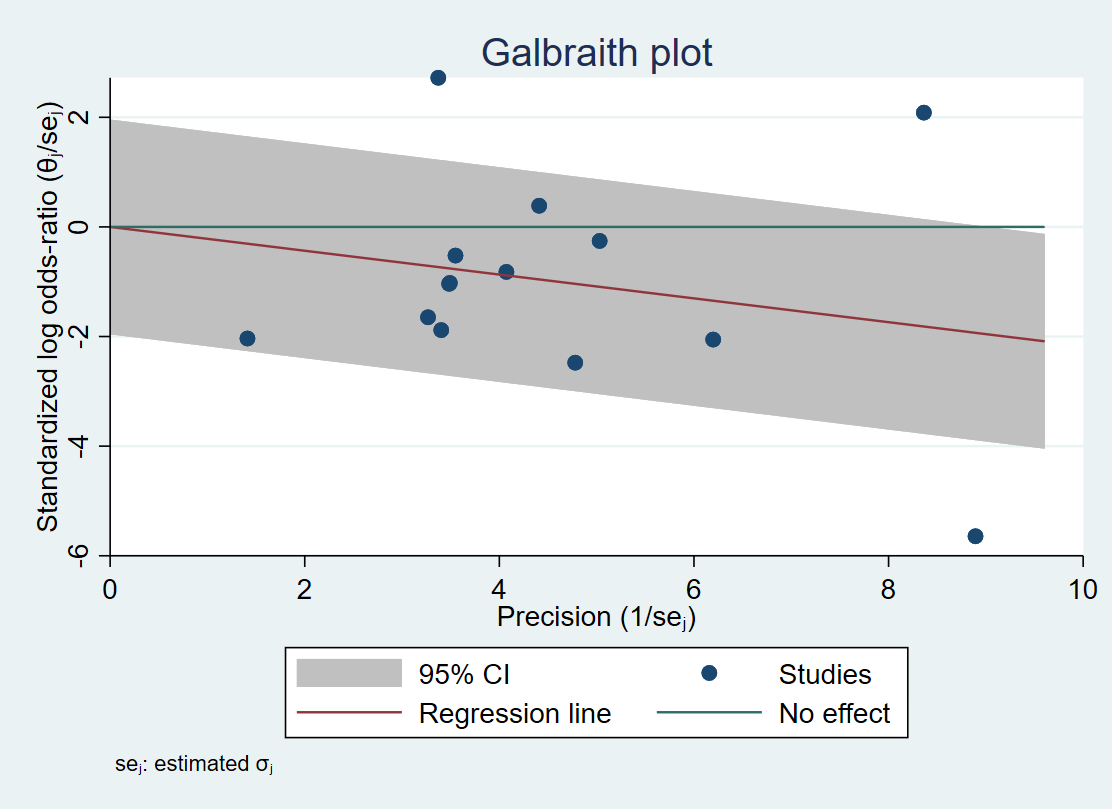


**Figure S47. Galbraith plot used to address heterogeneity of** **EMLO1 rs741301 polymorphism and DN risk under the dominant model in DN vs. DM patients**


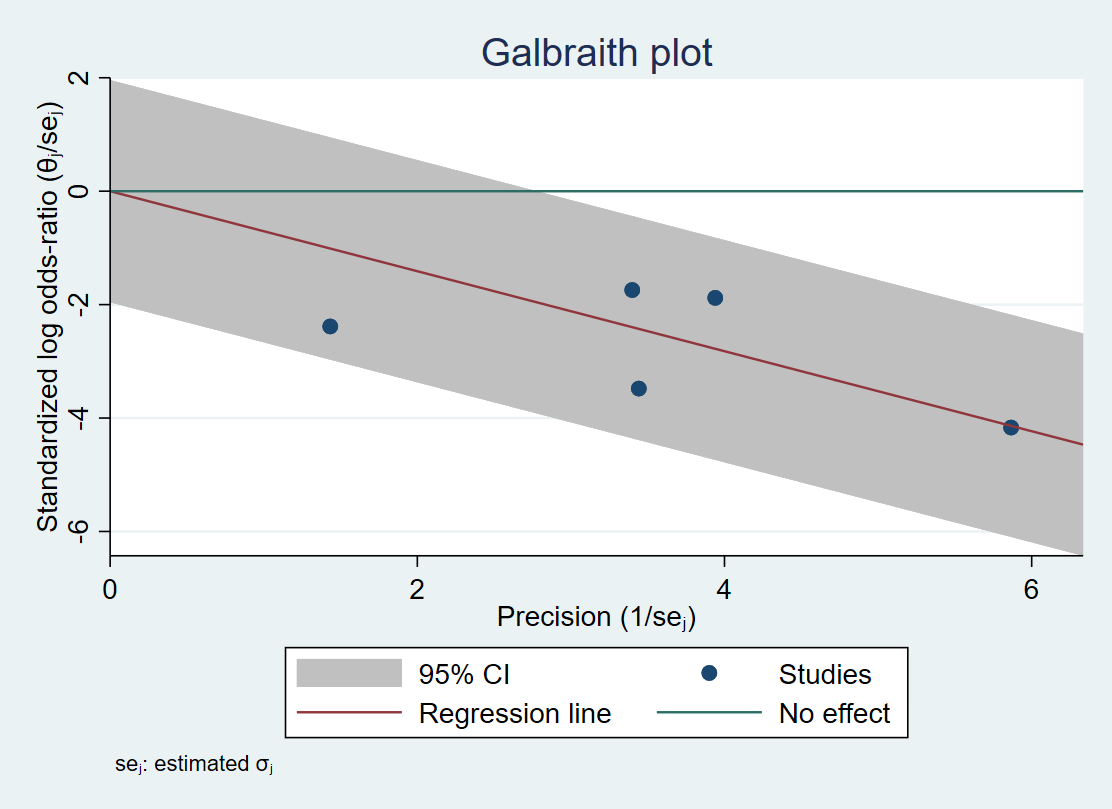


**Figure S48.** **Galbraith plot used to address heterogeneity of** **EMLO1 rs741301 polymorphism and DN risk under the dominant model in DN vs. healthy patients**


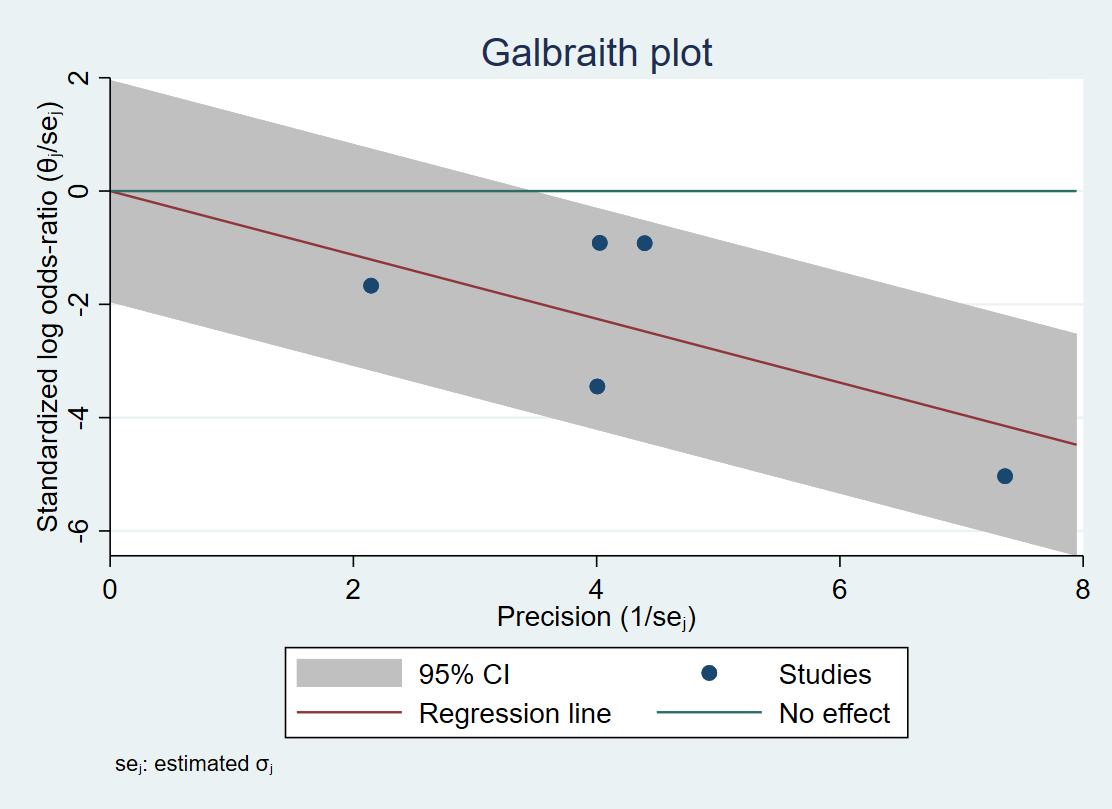


**Figure S49.** **Galbraith plot used to address heterogeneity of** **EMLO1 rs741301 polymorphism and DN risk under the dominant model in DM vs. healthy patients**


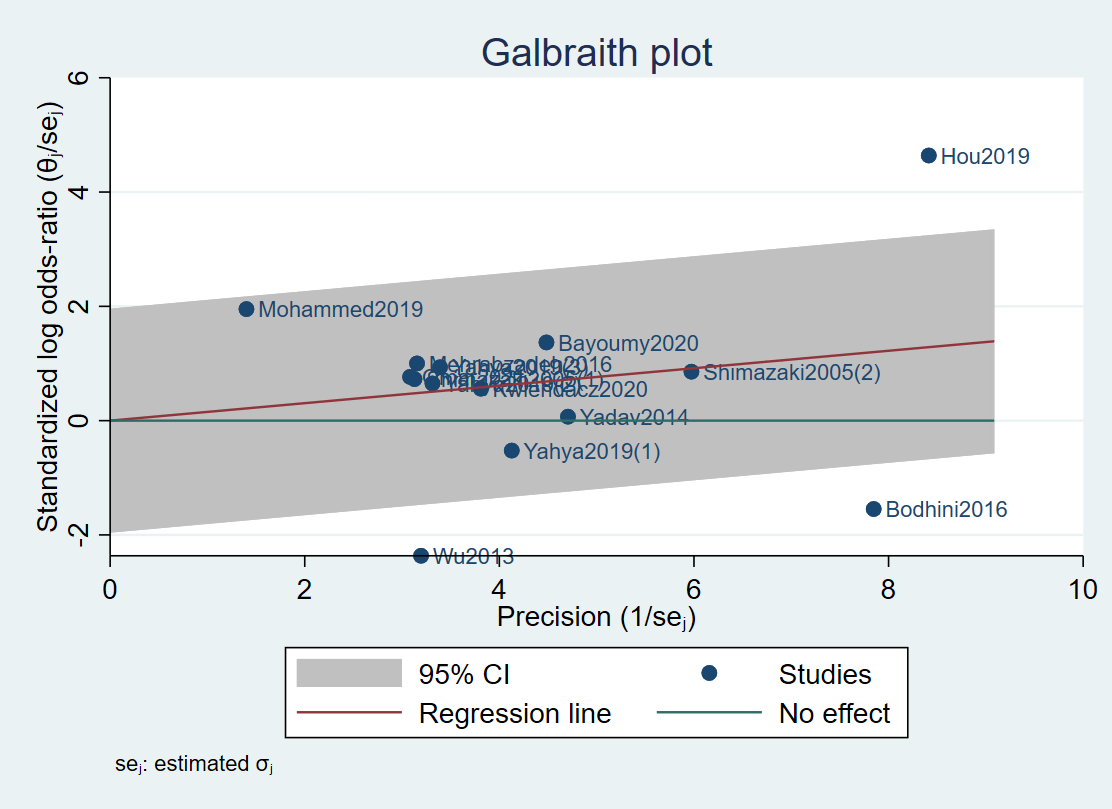


**Figure S50.** **Galbraith plot used to address heterogeneity of** **EMLO1 rs741301 polymorphism and DN risk under the codominant model in DN vs. DM patients**


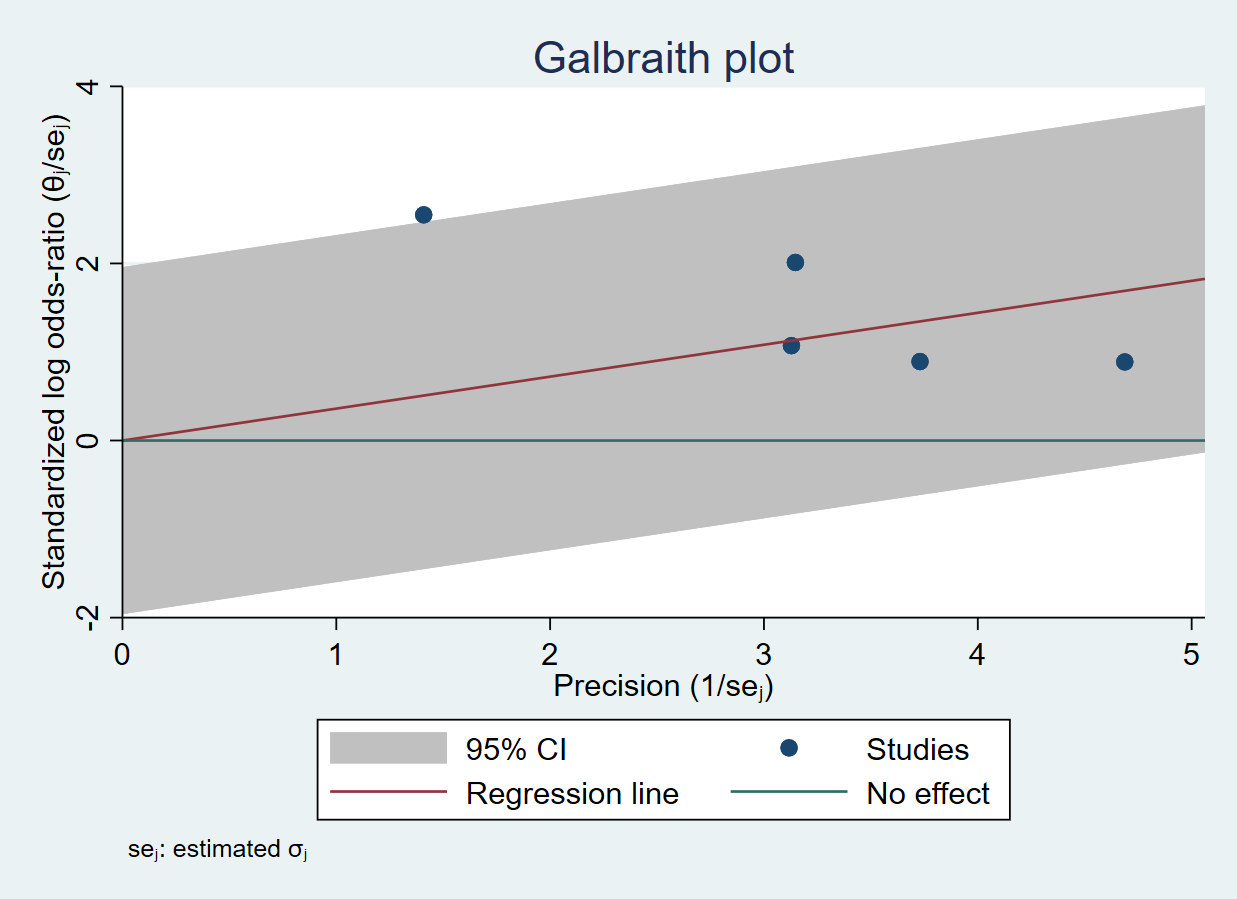


**Figure S51. Galbraith plot used to address heterogeneity of** **EMLO1 rs741301 polymorphism and DN risk under the codominant model in DN vs. healthy patients**


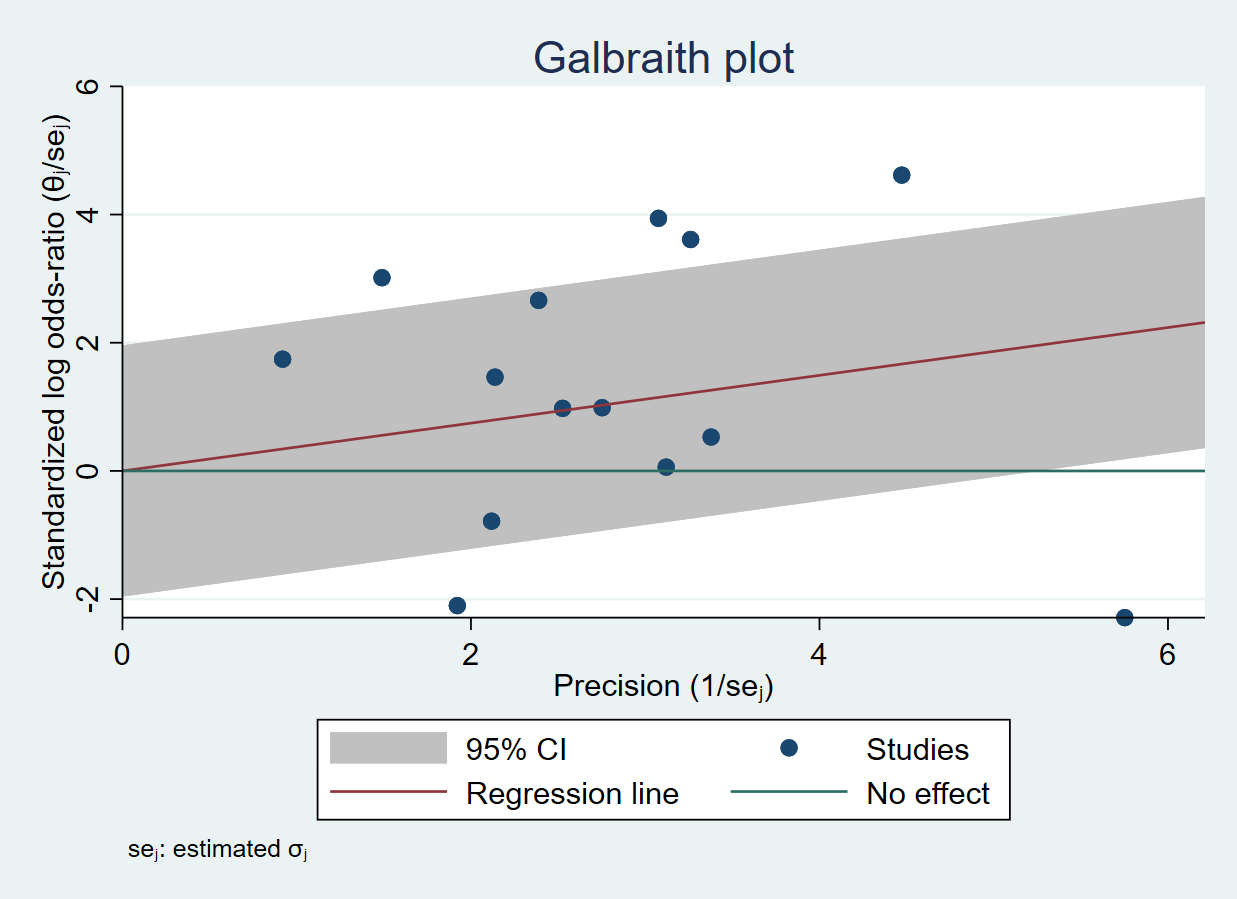


**Figure S52. Galbraith plot used to address heterogeneity of EMLO1 rs741301 polymorphism and DN risk under the homozygote model in DN vs. DM patients**


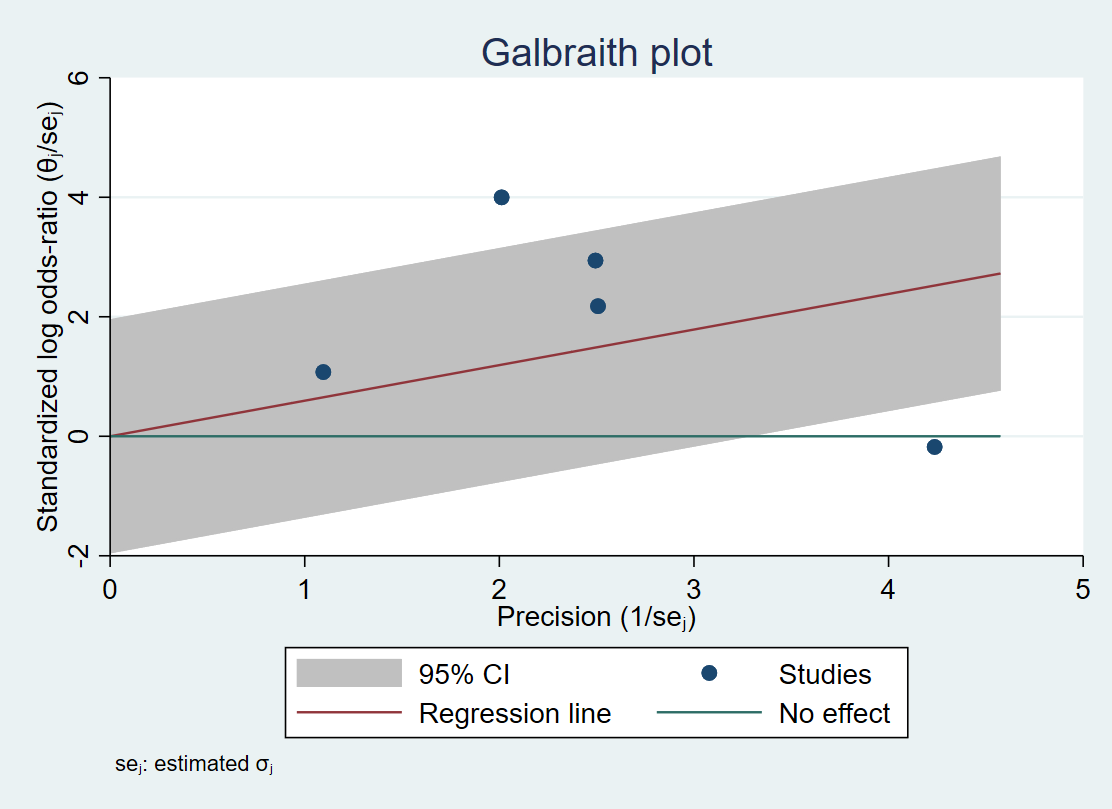


**Figure S53. Galbraith plot used to address heterogeneity of EMLO1 rs741301 polymorphism and DN risk under the homozygote model in DN vs. healthy patients**


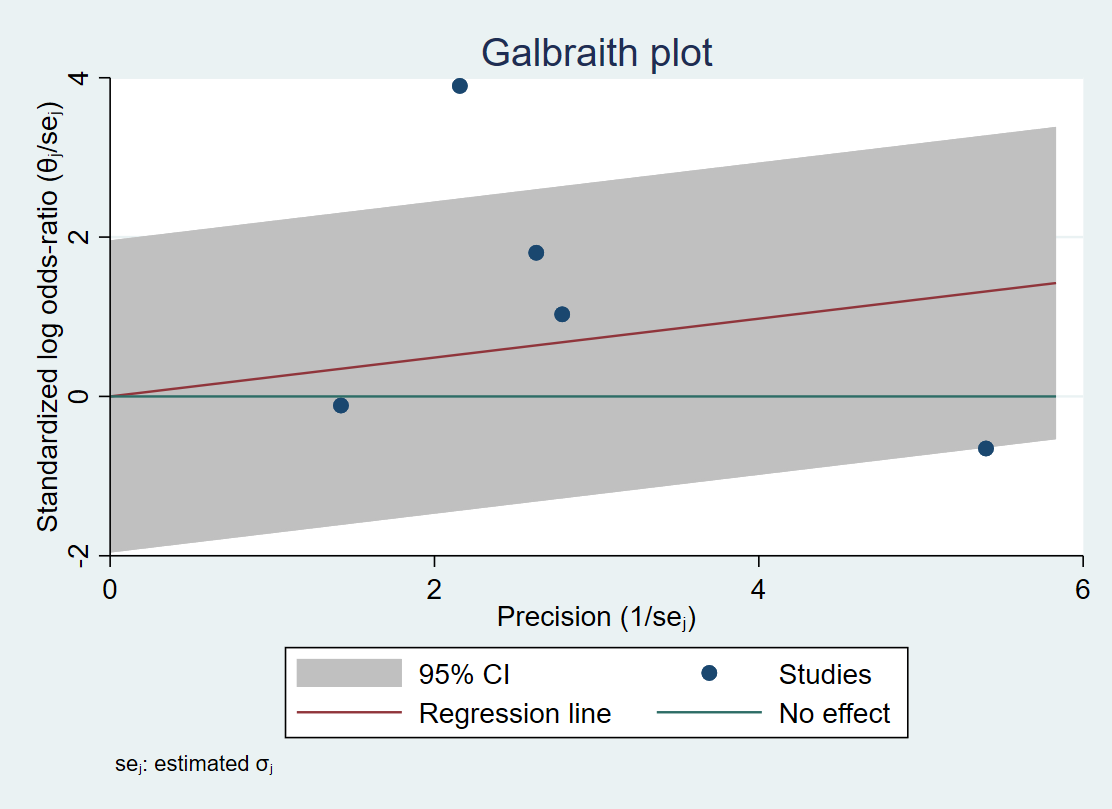


**Figure S54.** **Galbraith plot used to address heterogeneity of EMLO1 rs741301 polymorphism and DN risk under the homozygote model in DM vs. healthy patients**


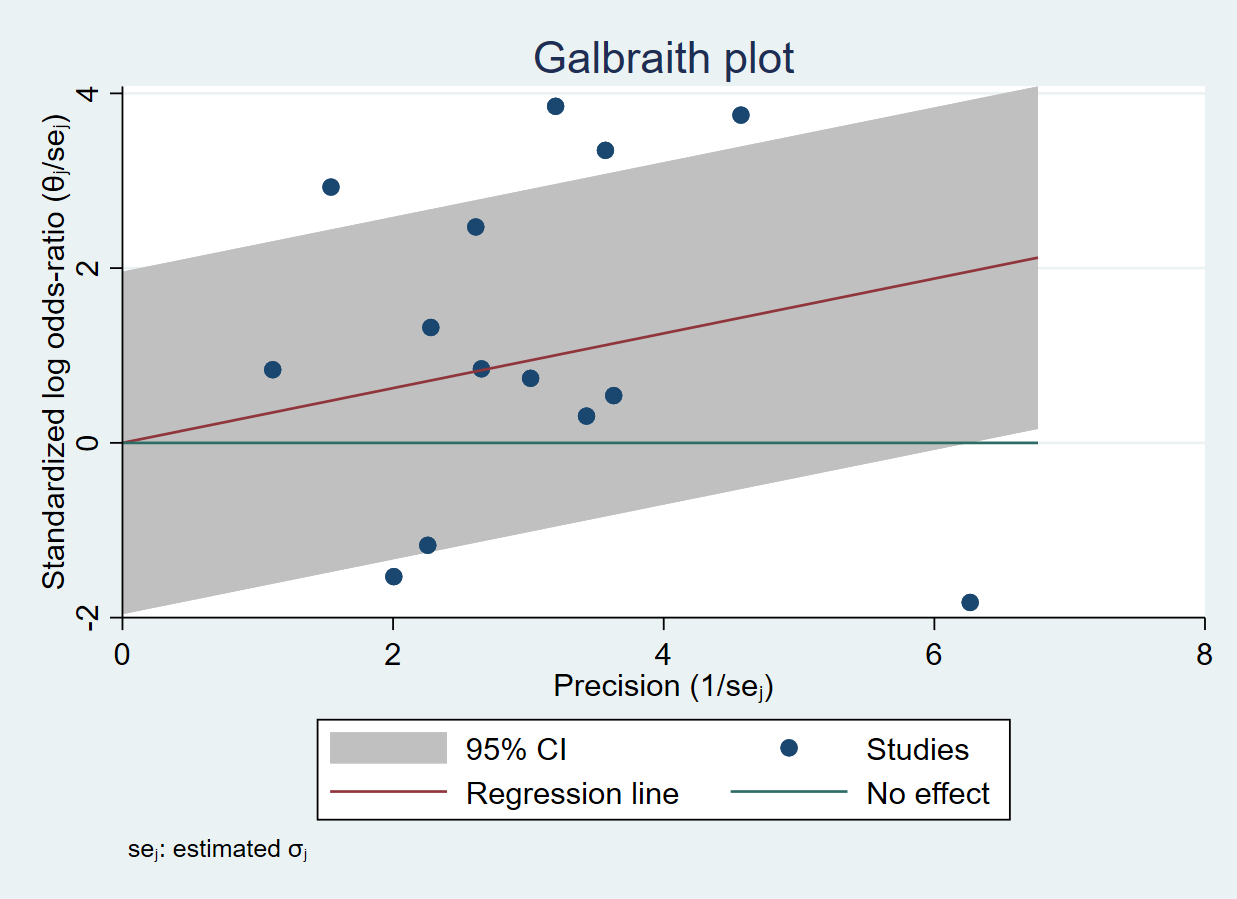


**Figure S55. Galbraith plot used to address heterogeneity of EMLO1 rs741301 polymorphism and DN risk under the recessive model in DN vs. DM patients**

**
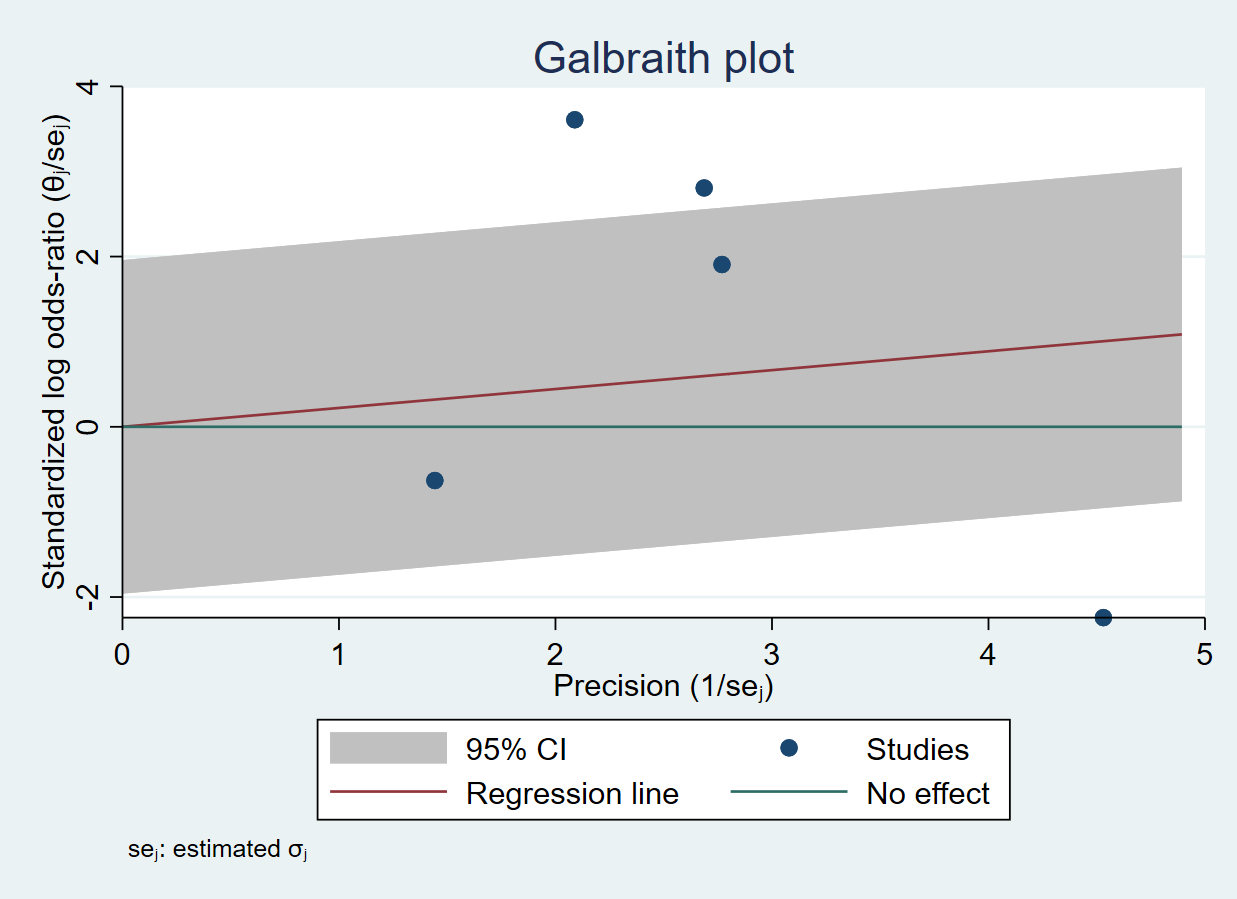
**

**Figure S56. Galbraith plot used to address heterogeneity of EMLO1 rs741301 polymorphism and DN risk under the recessive model in DN vs. healthy patients**


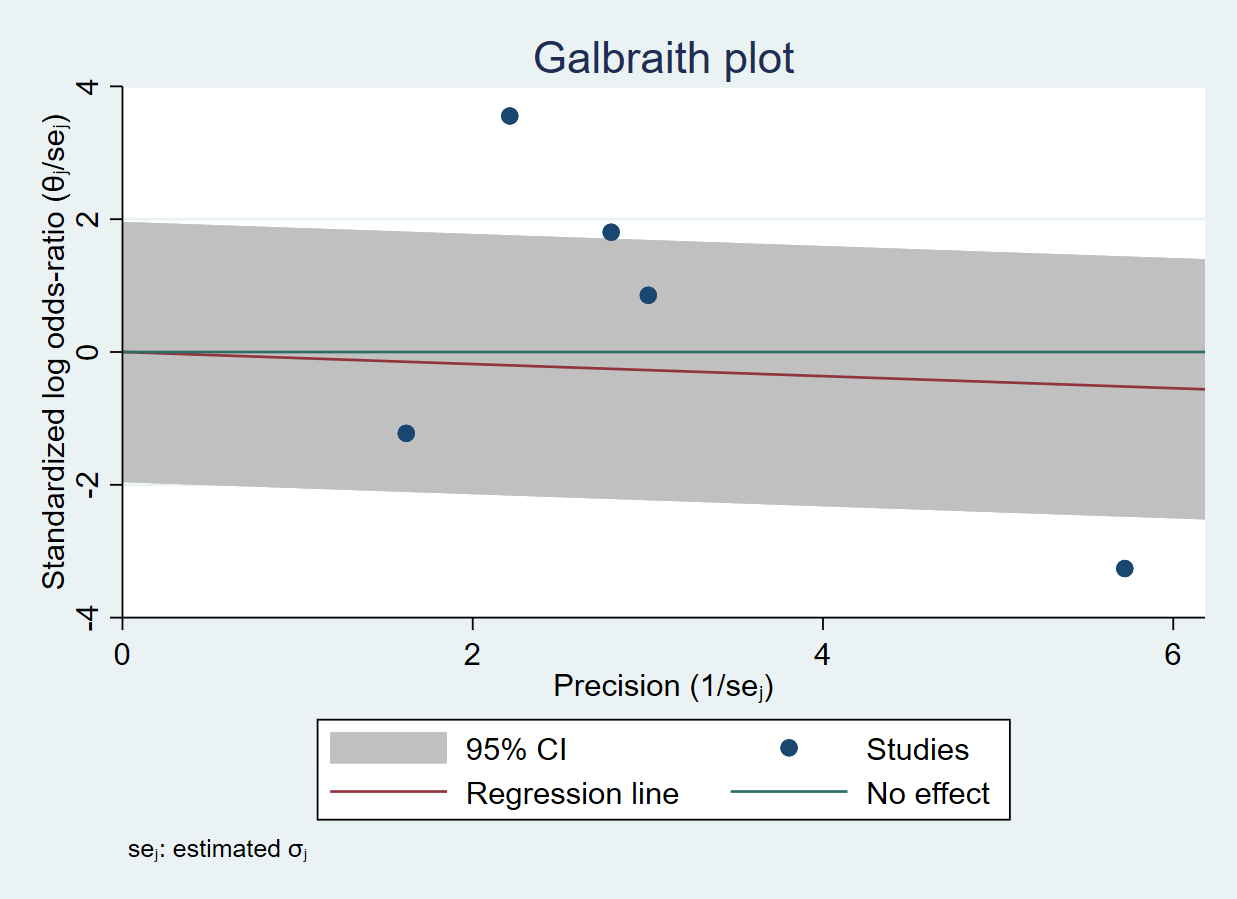


**Figure S57. Galbraith plot used to address heterogeneity of EMLO1 rs741301 polymorphism and DN risk under the recessive model in DM vs. healthy patients**


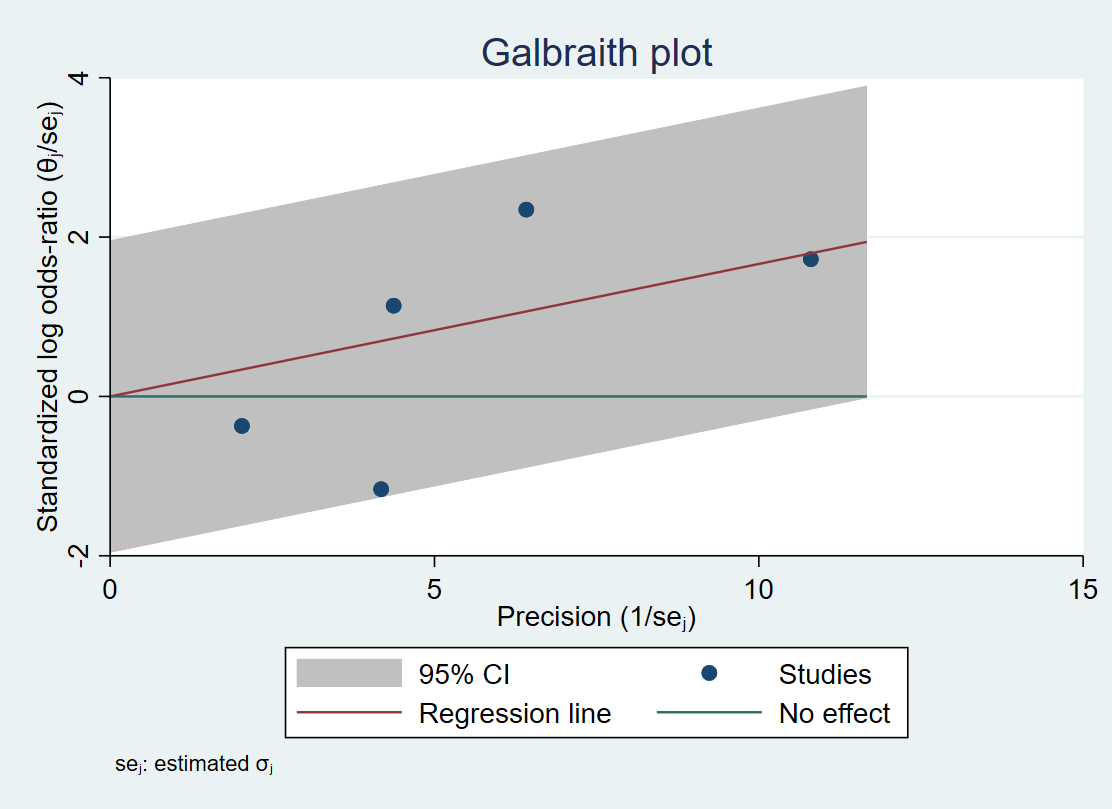


**Figure S58. Galbraith plot used to address heterogeneity of** **EMLO1 rs1345365 polymorphism and DN risk under the allele model in DN vs. DM patients**


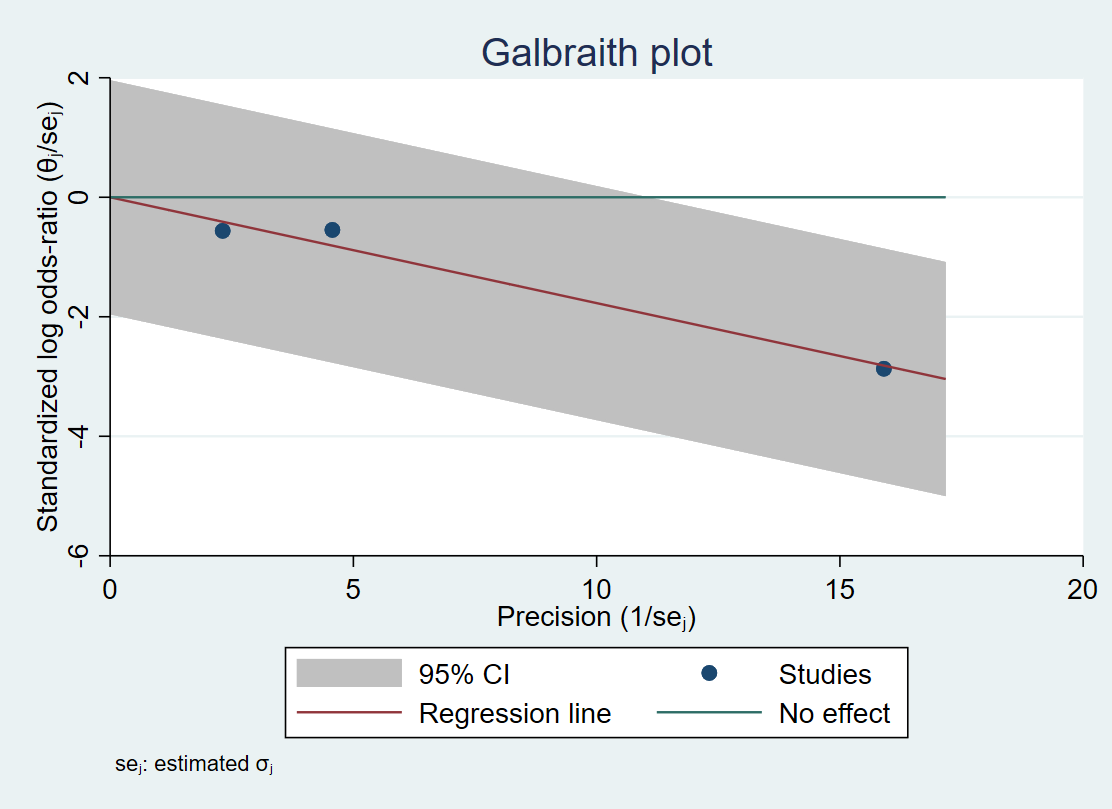


**Figure S59. Galbraith plot used to address heterogeneity of** **EMLO1 rs1345365 polymorphism and DN risk under the allele model in DN vs. healthy patients**


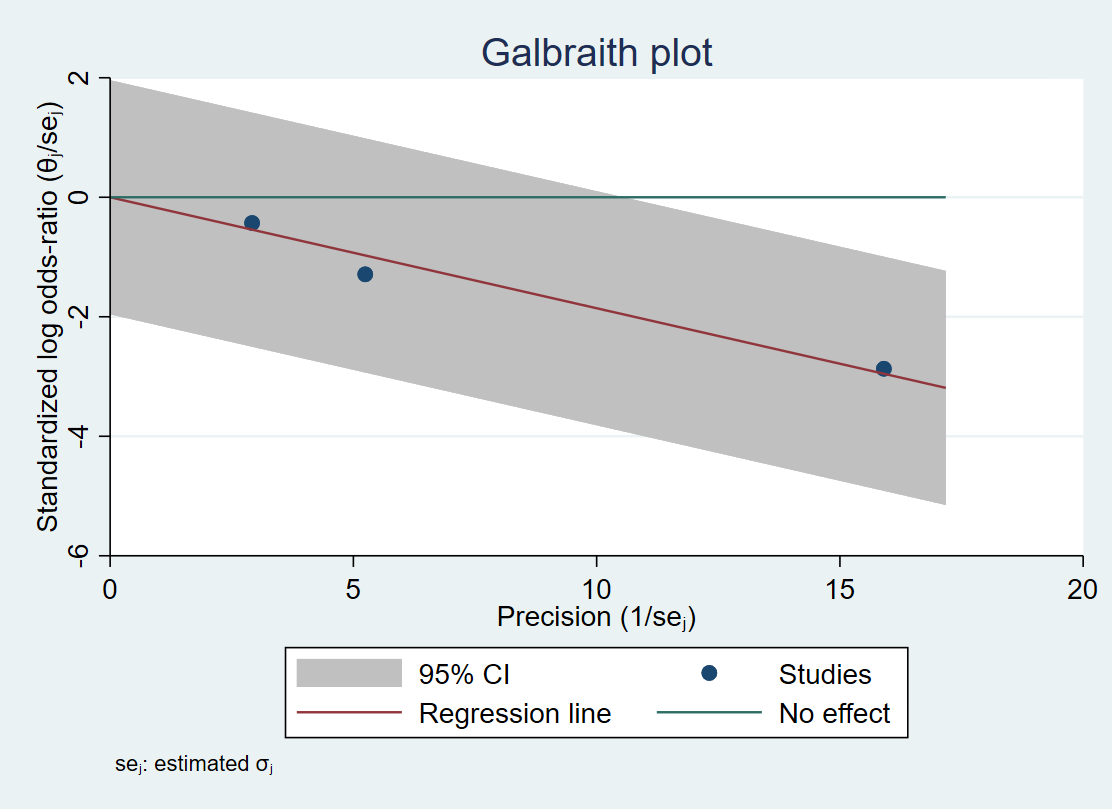


**Figure S60. Galbraith plot used to address heterogeneity of** **EMLO1 rs1345365 polymorphism and DN risk under the allele model in DM vs. healthy patients**


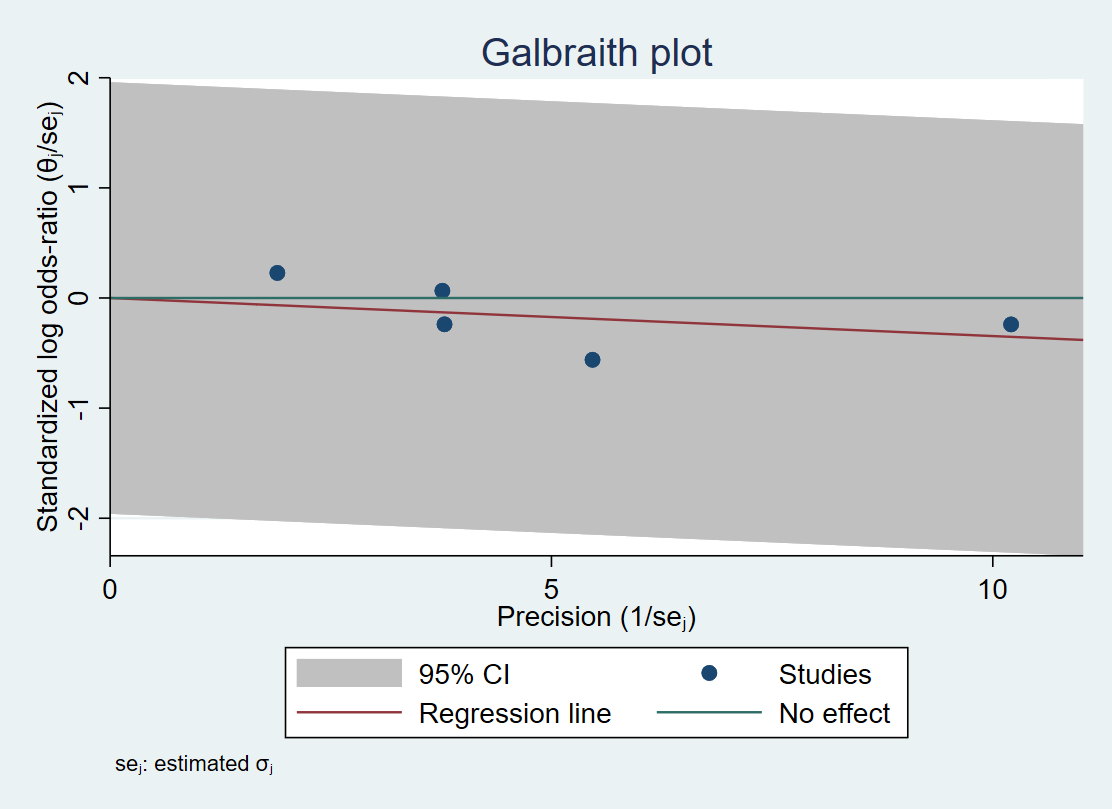


**Figure S61. Galbraith plot used to address heterogeneity of** **EMLO1 rs1345365 polymorphism and DN risk under the dominant model in DN vs. DM patients**


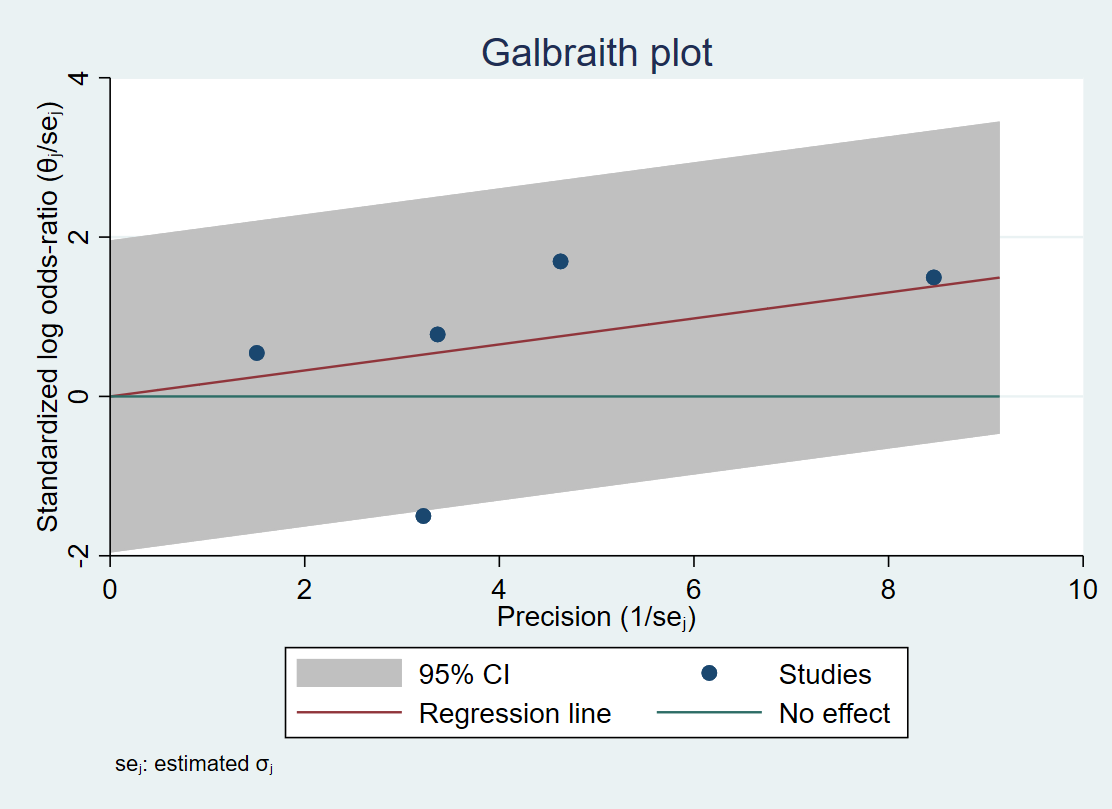


**Figure S62.** **Galbraith plot used to address heterogeneity of** **EMLO1 rs1345365 polymorphism and DN risk under the codominant model in DN vs. DM patients**


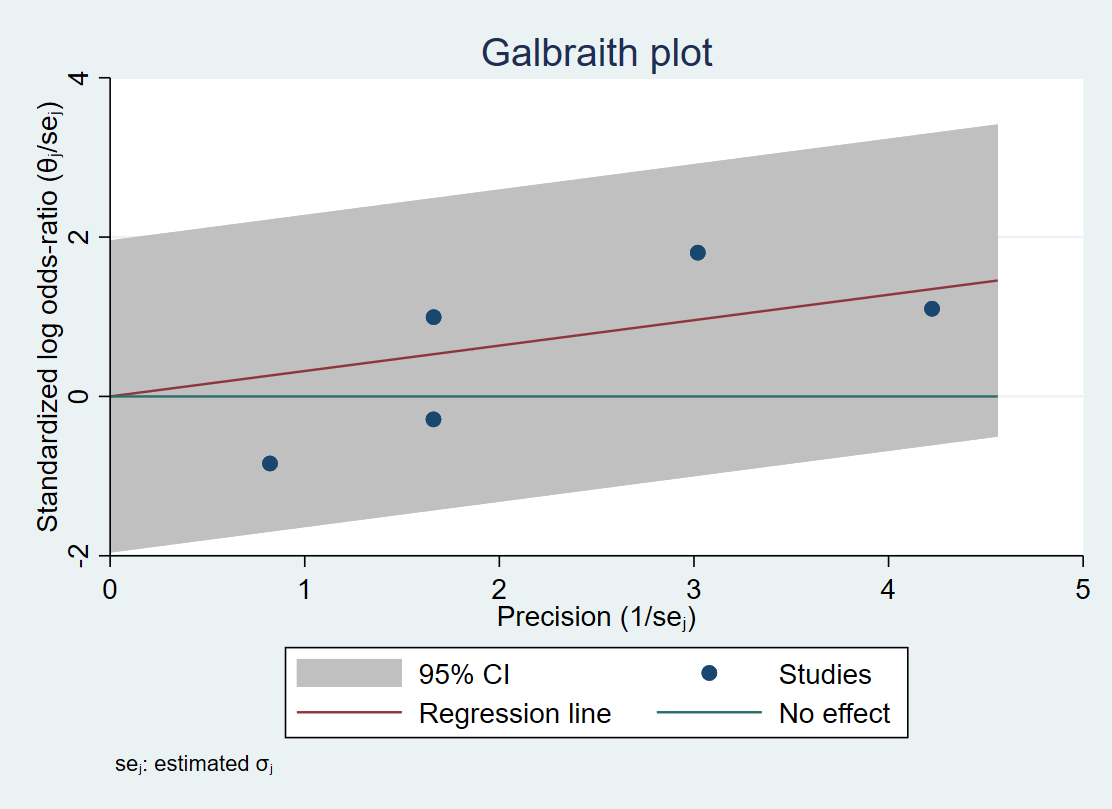


**Figure S63. Galbraith plot used to address heterogeneity of** **EMLO1 rs1345365 polymorphism and DN risk under the homozygote model in DN vs. DM patients**


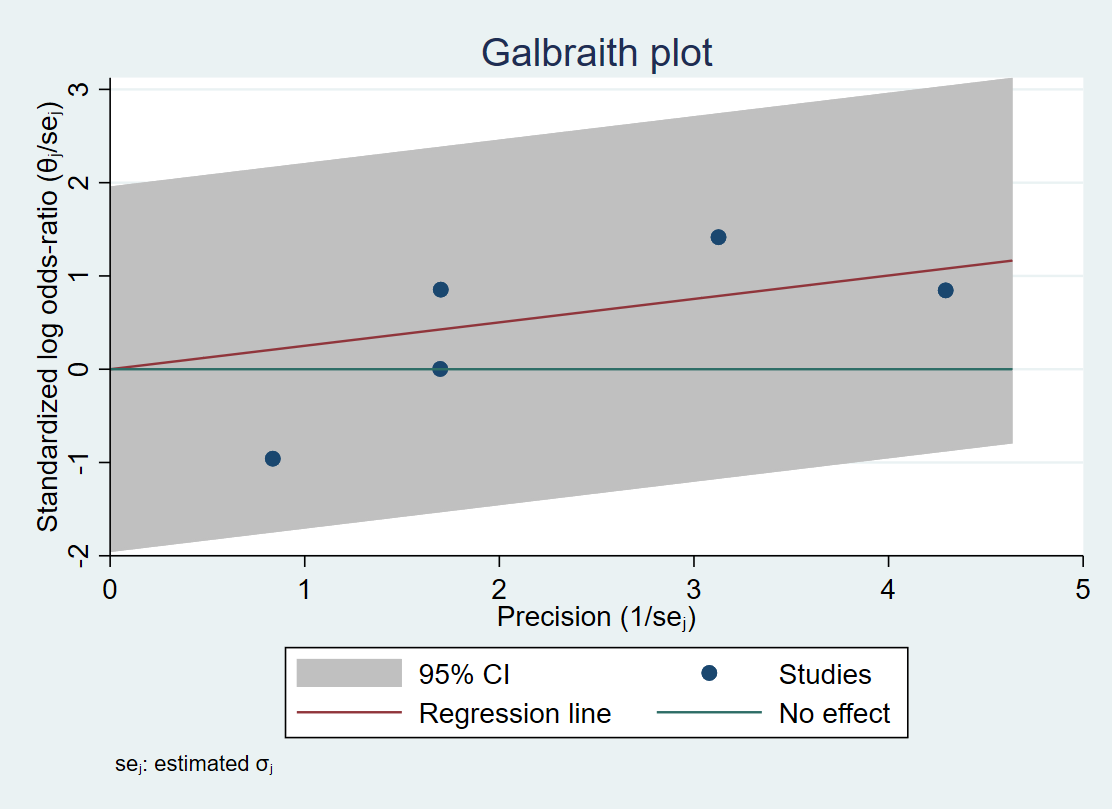


**Figure S64. Galbraith plot used to address heterogeneity of** **EMLO1 rs1345365 polymorphism and DN risk under the recessive model in DN vs. DM patients**


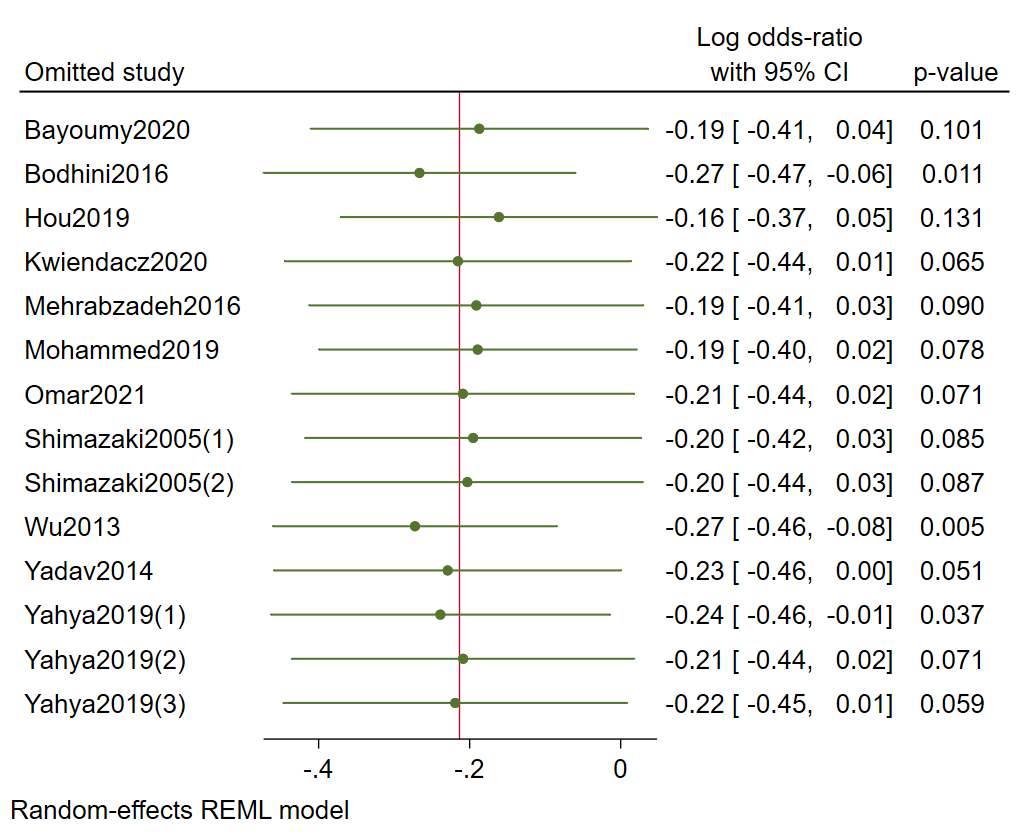


**Figure S65.** **Sensitivity analysis of** **EMLO1 rs741301 polymorphism and DN risk under the dominant model in DN vs. DM patients**


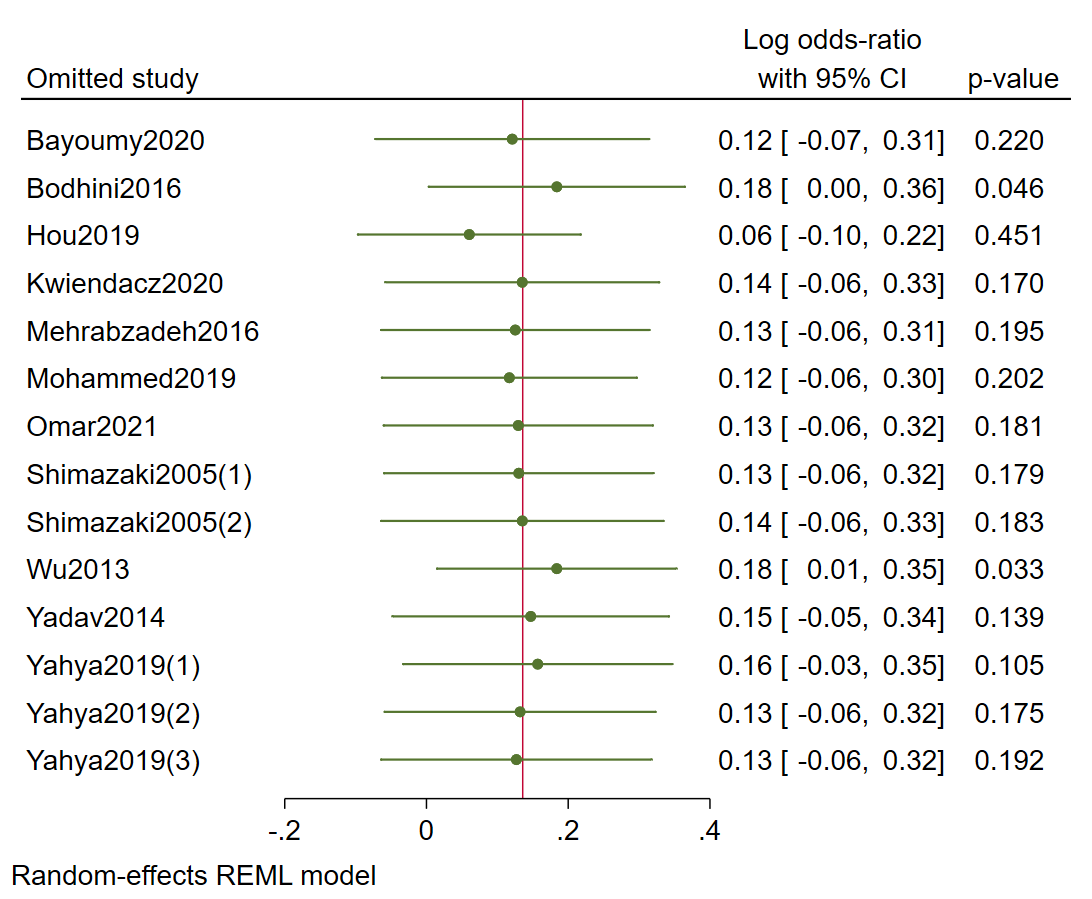


**Figure S66. Sensitivity analysis of** **EMLO1 rs741301 polymorphism and DN risk under the codominant model in DN vs. DM patients**


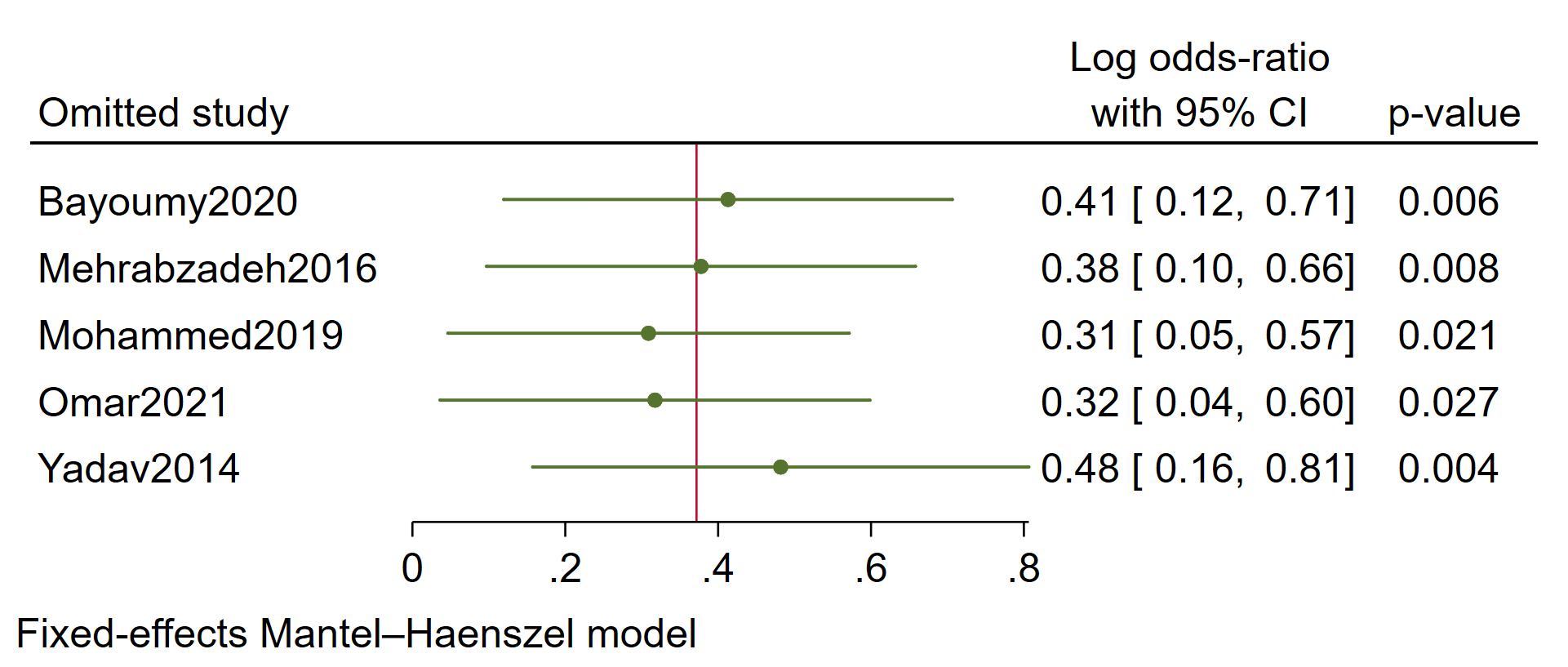


**Figure S67. Sensitivity analysis of** **EMLO1 rs741301 polymorphism and DN risk under the codominant model in DN vs. healthy patients**


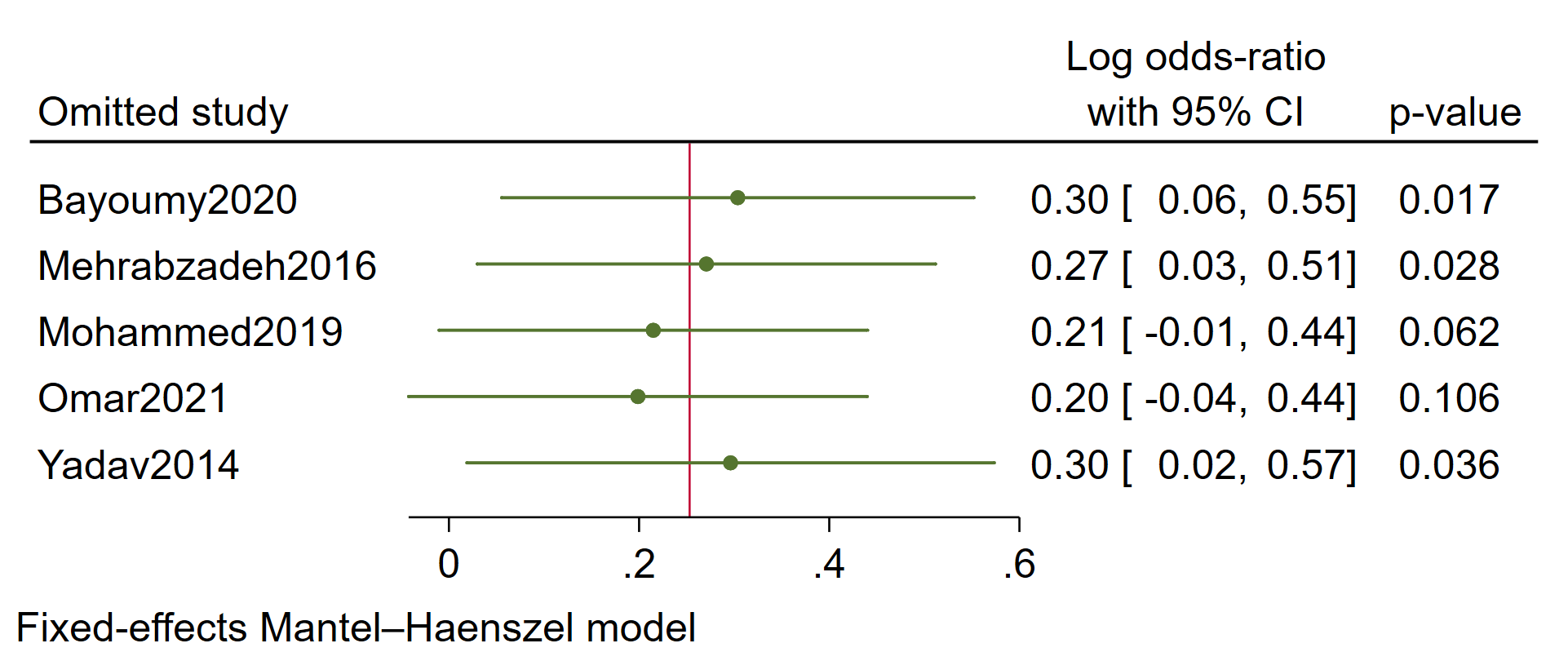


**Figure S68.** **Sensitivity analysis of** **EMLO1 rs741301 polymorphism and DN risk under the codominant model in DM vs. healthy patients**


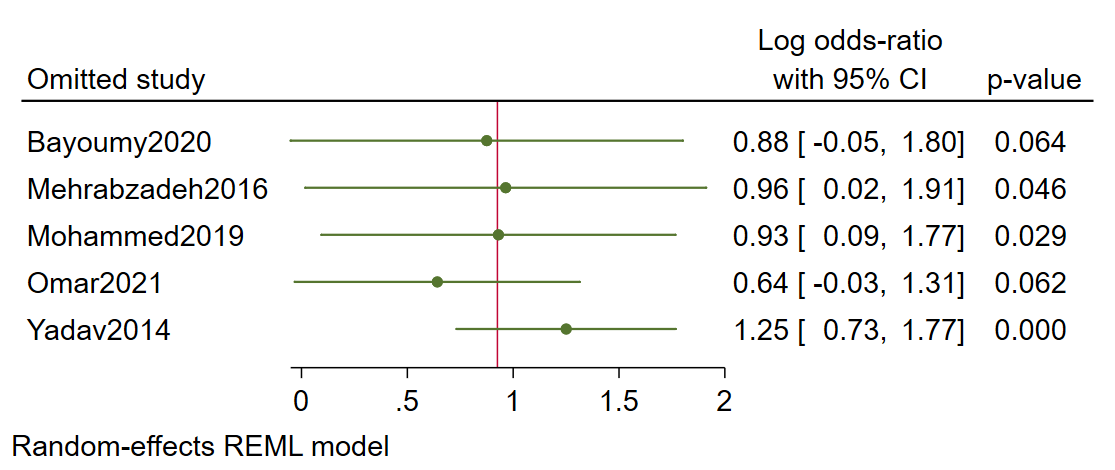


**Figure S69.** **Sensitivity analysis of** **EMLO1 rs741301 polymorphism and DN risk under the homozygote model in DN vs. healthy patients**


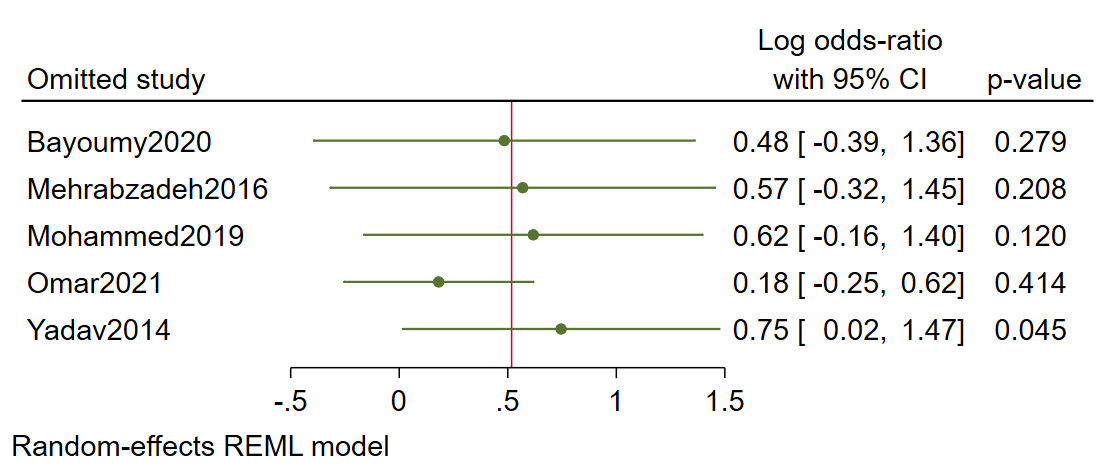


**Figure S70.** **Sensitivity analysis of** **EMLO1 rs741301 polymorphism and DN risk under the homozygote model in DM vs. healthy patients**


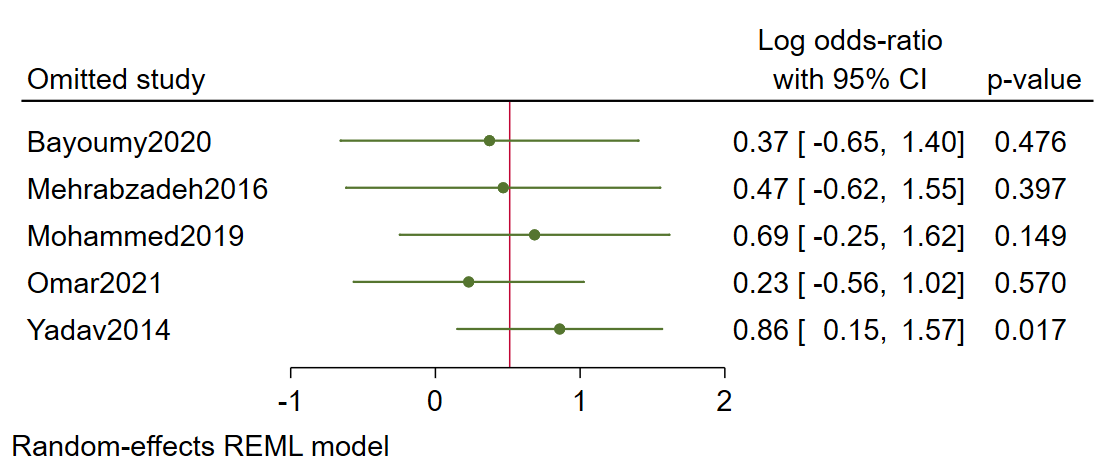


**Figure S71. Sensitivity analysis of** **EMLO1 rs741301 polymorphism and DN risk under the recessive model in DN vs. healthy patients**


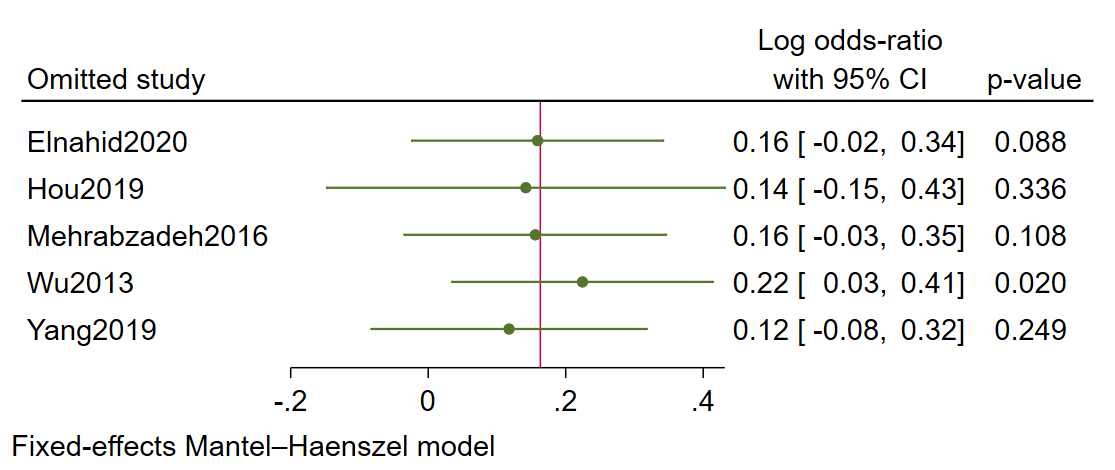


**Figure S72. Sensitivity analysis of** **EMLO1 rs1345365 polymorphism and DN risk under the codominant model in DN vs. DM patients**


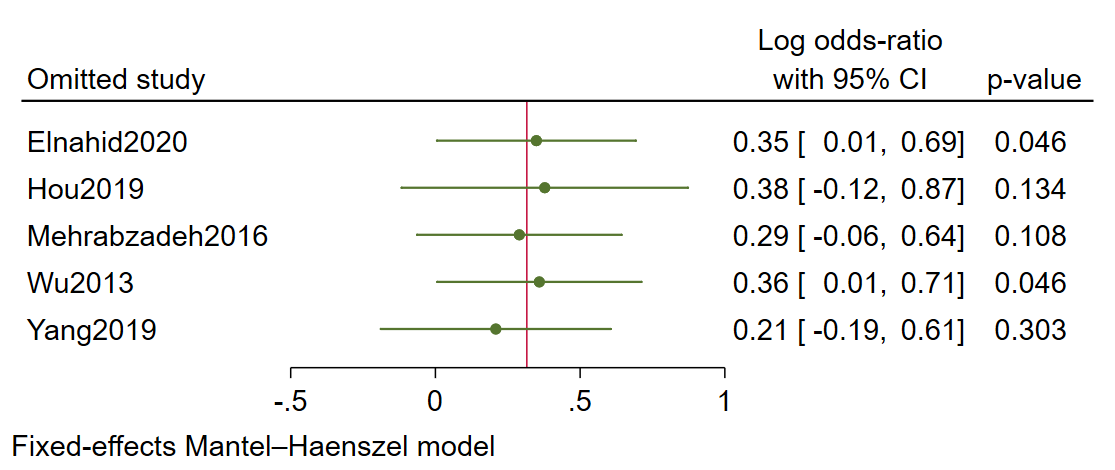


**Figure S73. Sensitivity analysis of** **EMLO1 rs1345365 polymorphism and DN risk under the homozygote model in DN vs. DM patients**


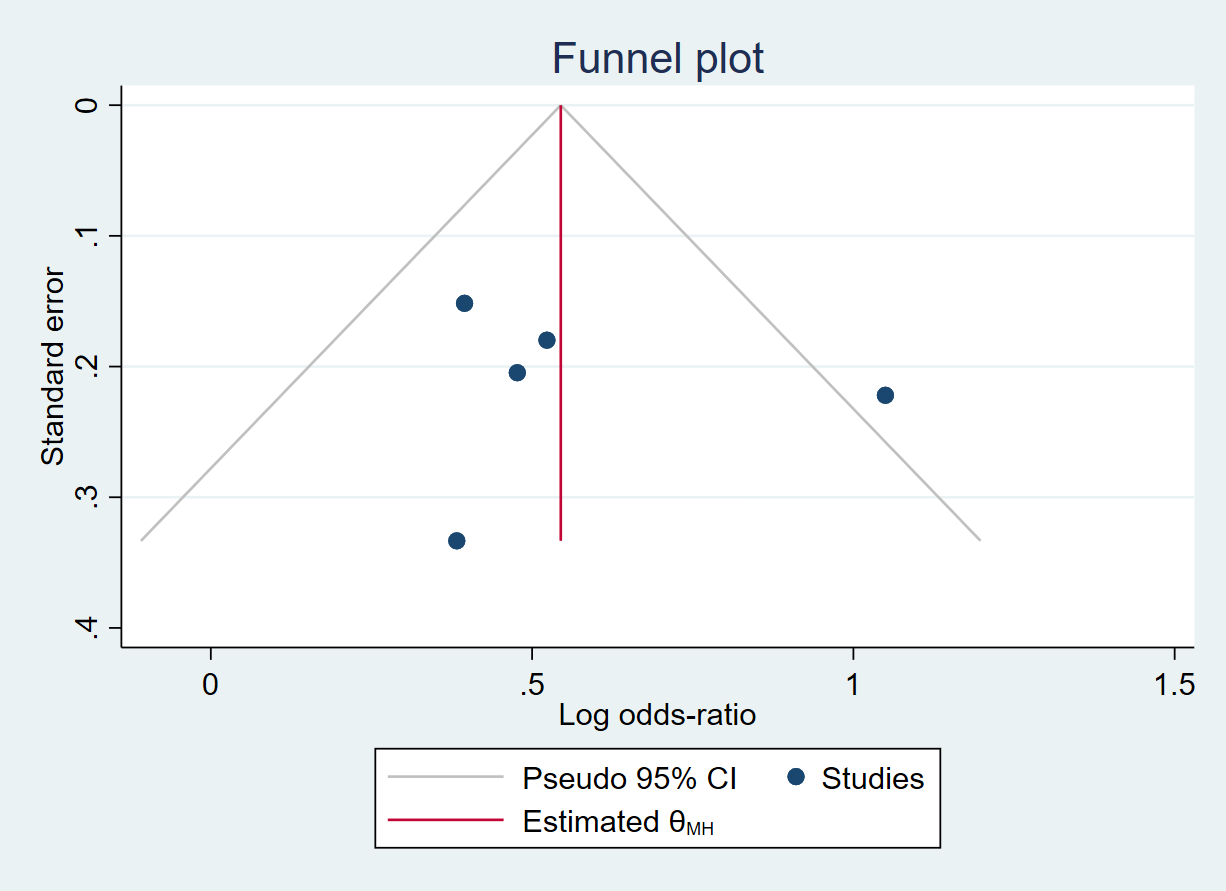


**Figure S74**. **Begg’s funnel plot of publication bias for the association between EMLO1 rs741301 polymorphisms and DN risk under the allele model in DN vs. healthy patients**


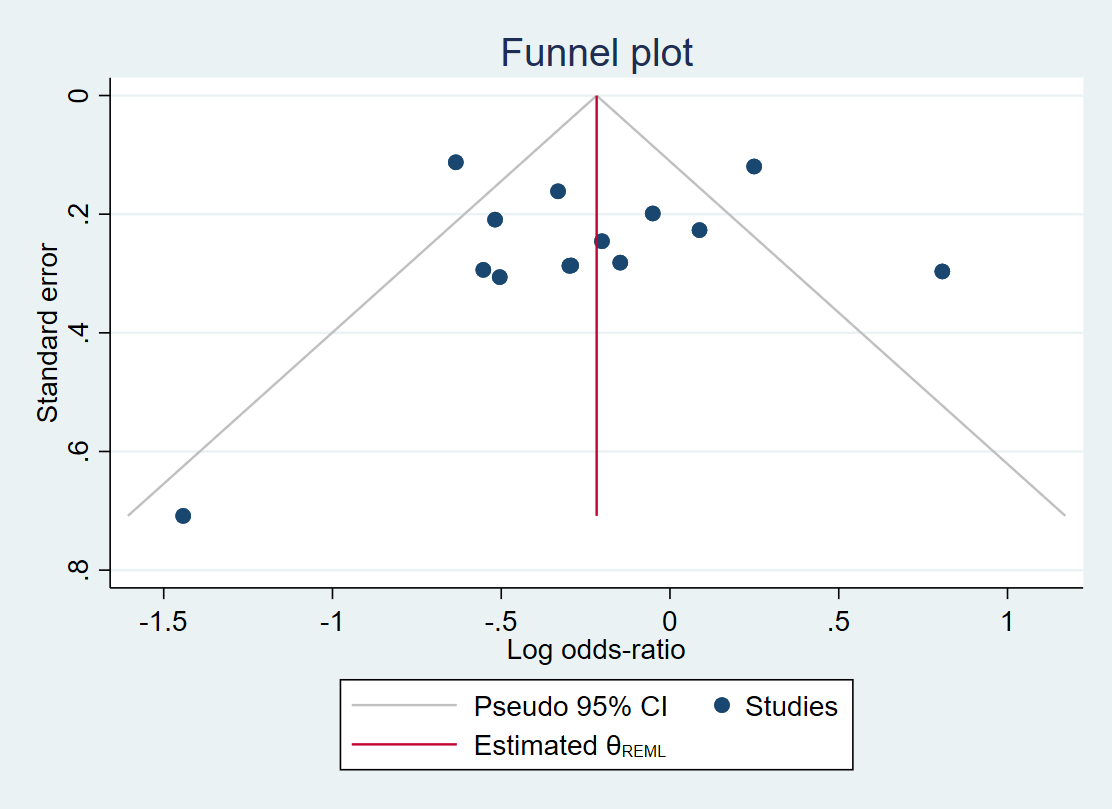


**Figure S75**. **Begg’s funnel plot of publication bias for the association between EMLO1 rs741301 polymorphisms and DN risk under the dominant model in DN vs. DM patients**


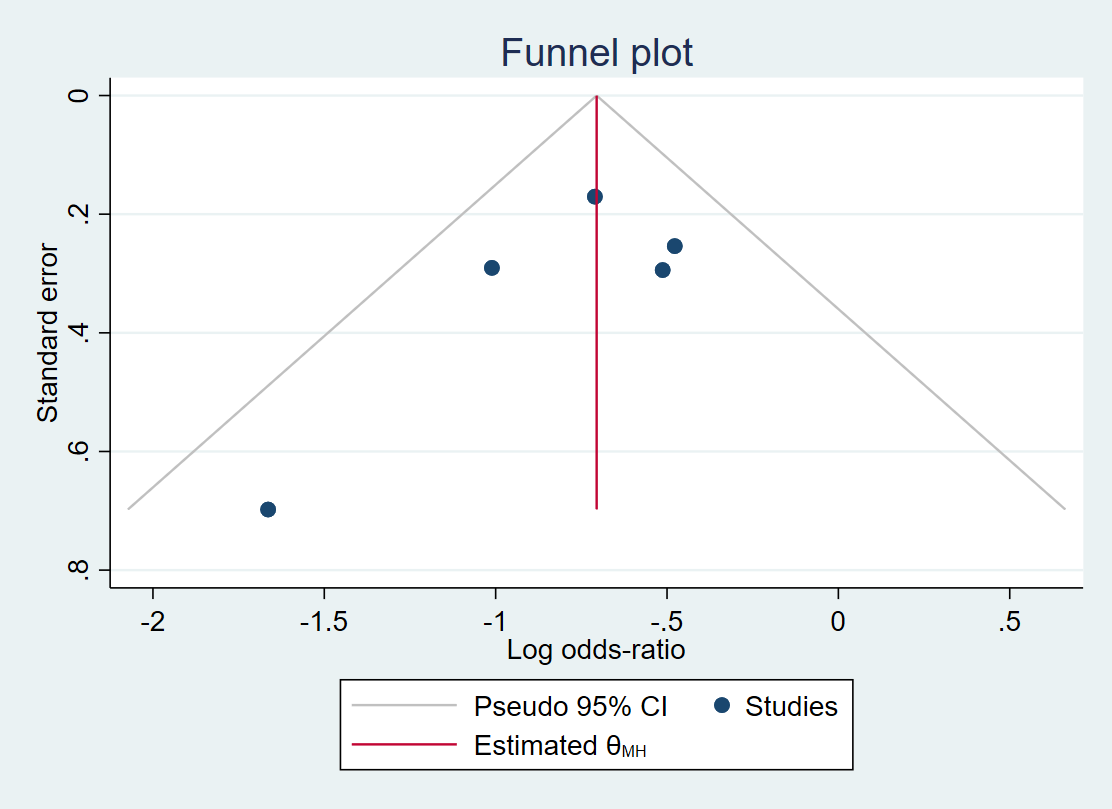


**Figure S76.** **Begg’s funnel plot of publication bias for the association between EMLO1 rs741301 polymorphisms and DN risk under the dominant model in DN vs. healthy patients**


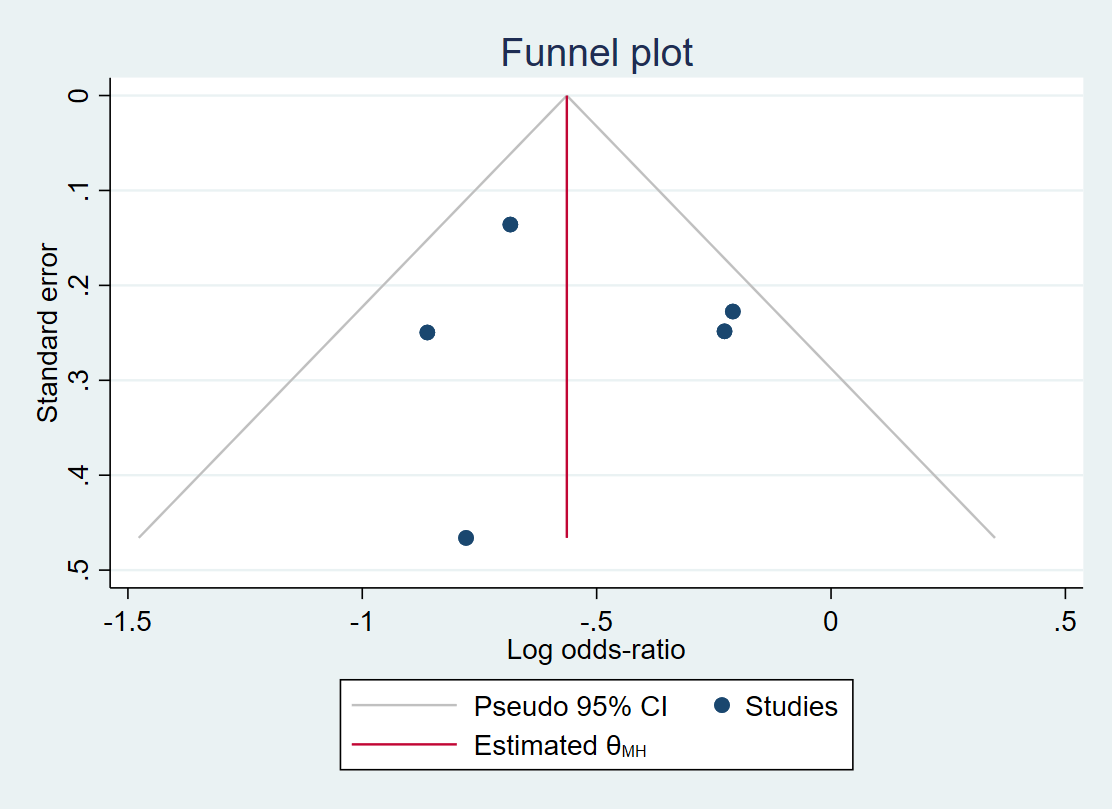


**Figure S77.** **Begg’s funnel plot of publication bias for the association between EMLO1 rs741301 polymorphisms and DN risk under the dominant model in DM vs. healthy patients**


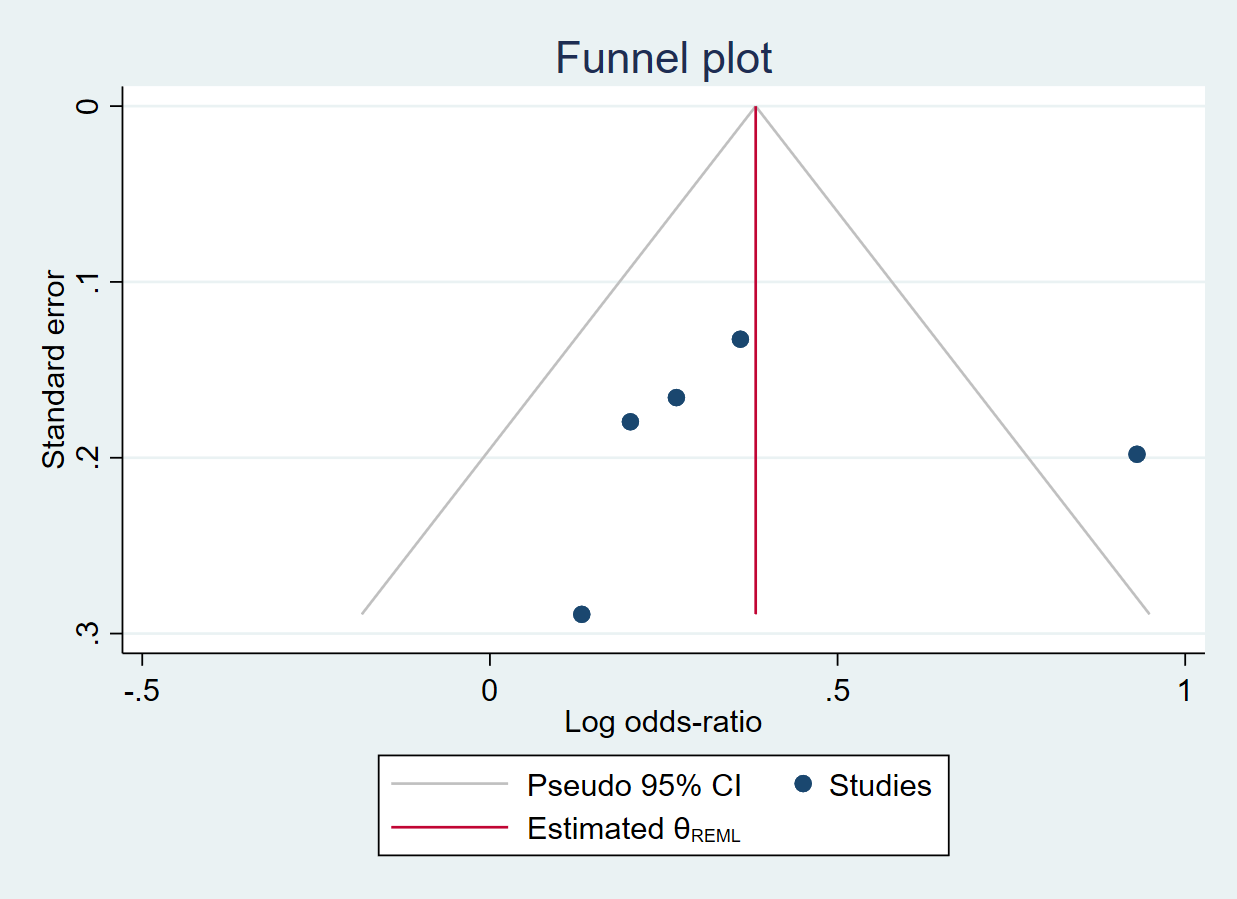


**Figure S78.** **Begg’s funnel plot of publication bias for the association between EMLO1 rs741301 polymorphisms and DN risk under the allele model in DM vs. healthy patients**


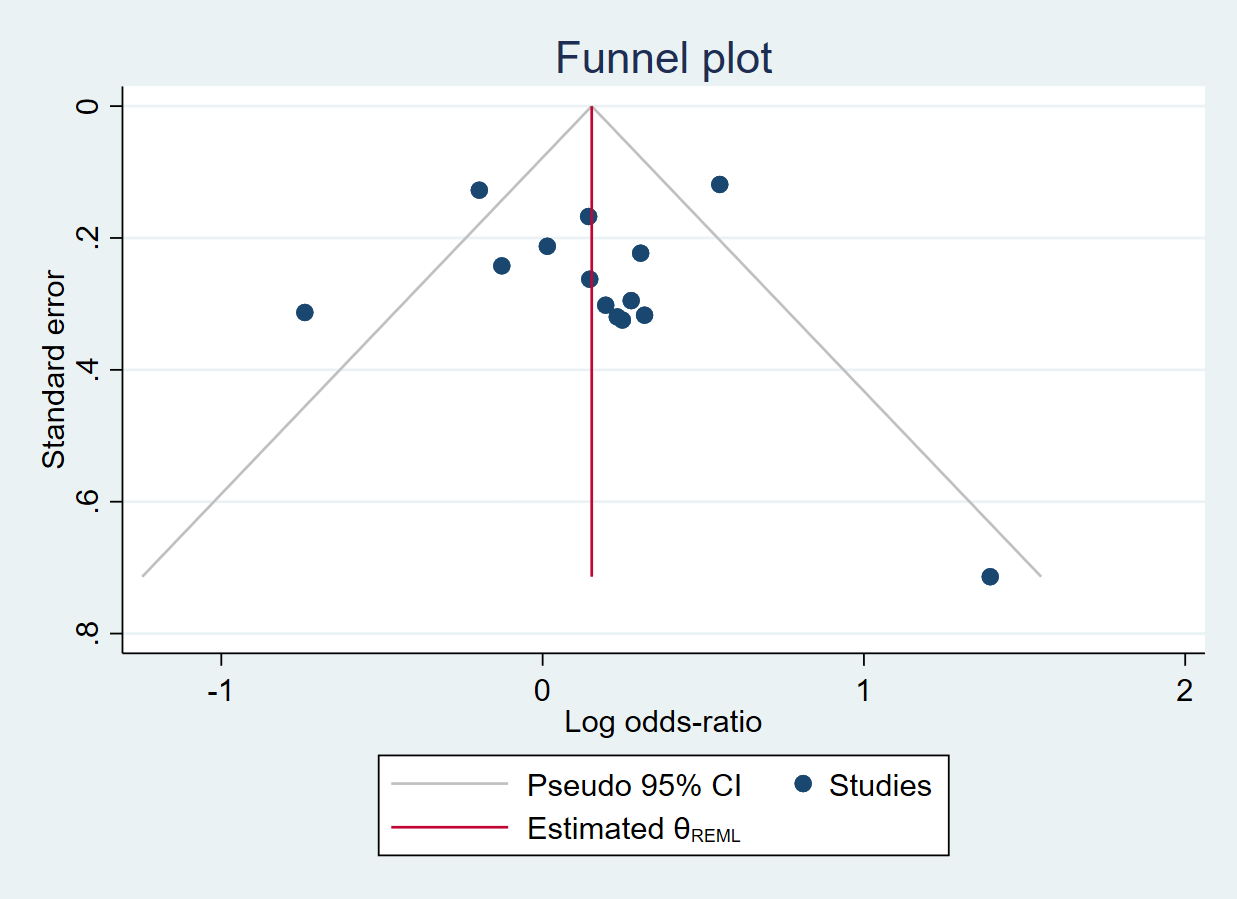


**Figure S79.** **Begg’s funnel plot of publication bias for the association between EMLO1 rs741301 polymorphisms and DN risk under the codominant model in DN vs. DM patients**


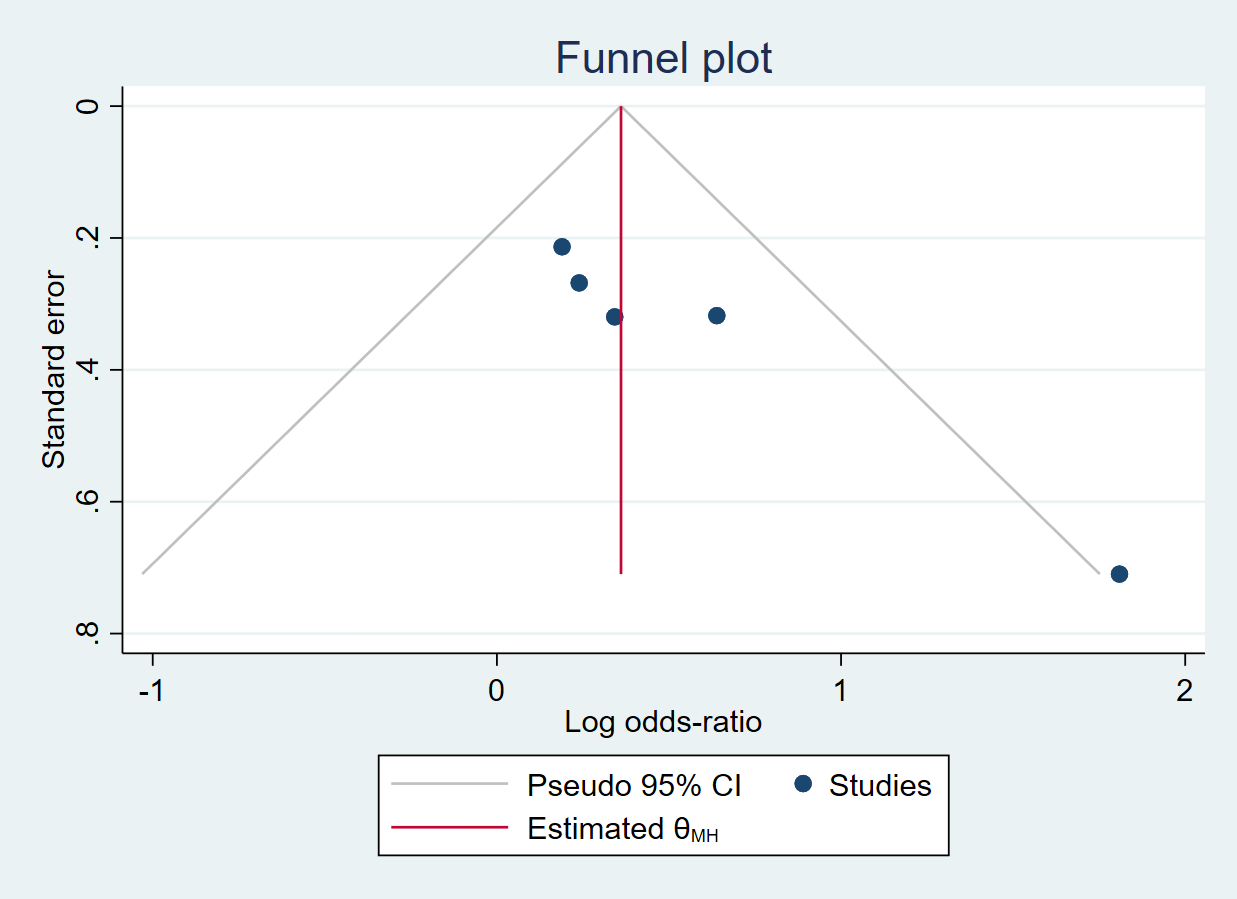


**Figure S80.** **Begg’s funnel plot of publication bias for the association between EMLO1 rs741301 polymorphisms and DN risk under the codominant model in DN vs. healthy patients**


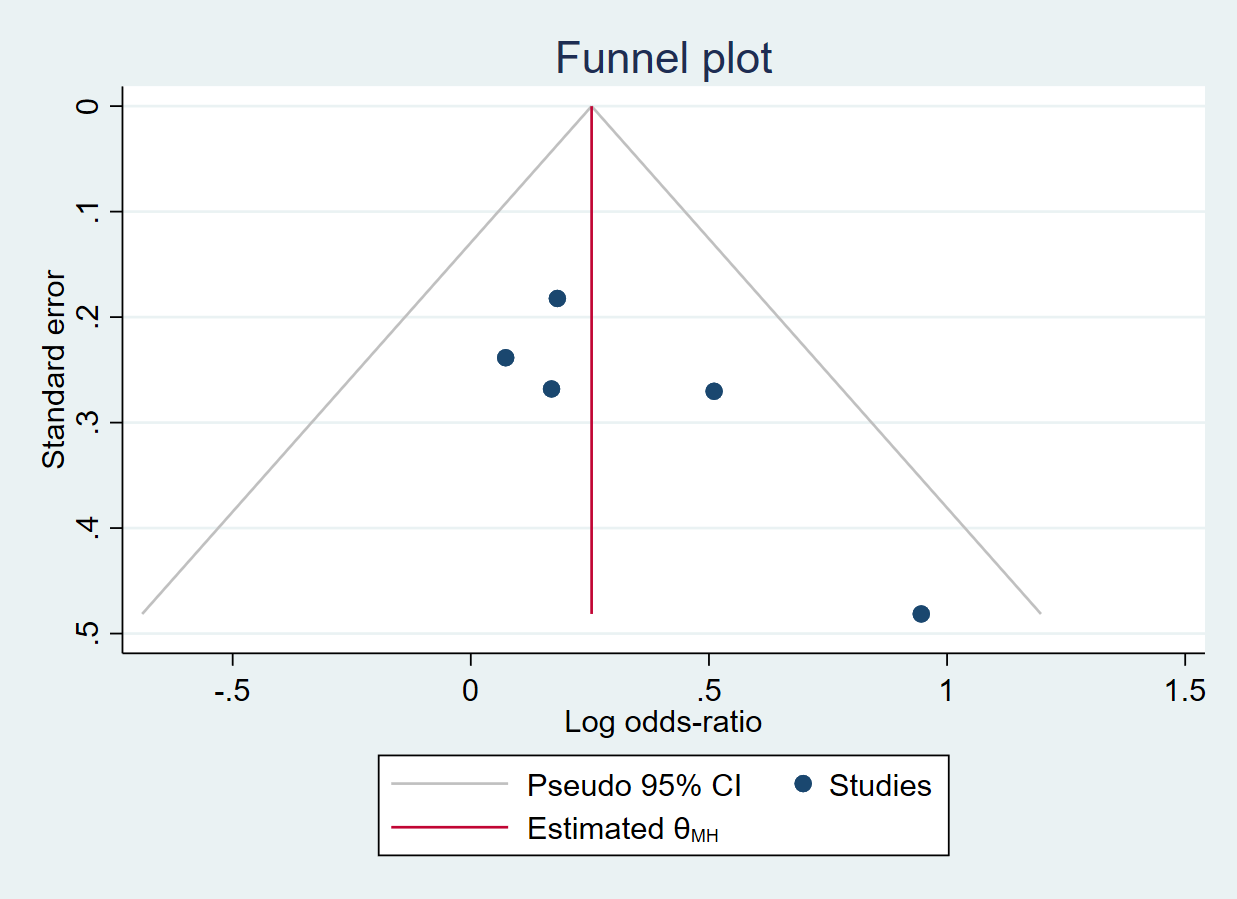


**Figure S81.** **Begg’s funnel plot of publication bias for the association between EMLO1 rs741301 polymorphisms and DN risk under the codominant model in DM vs. healthy patients**


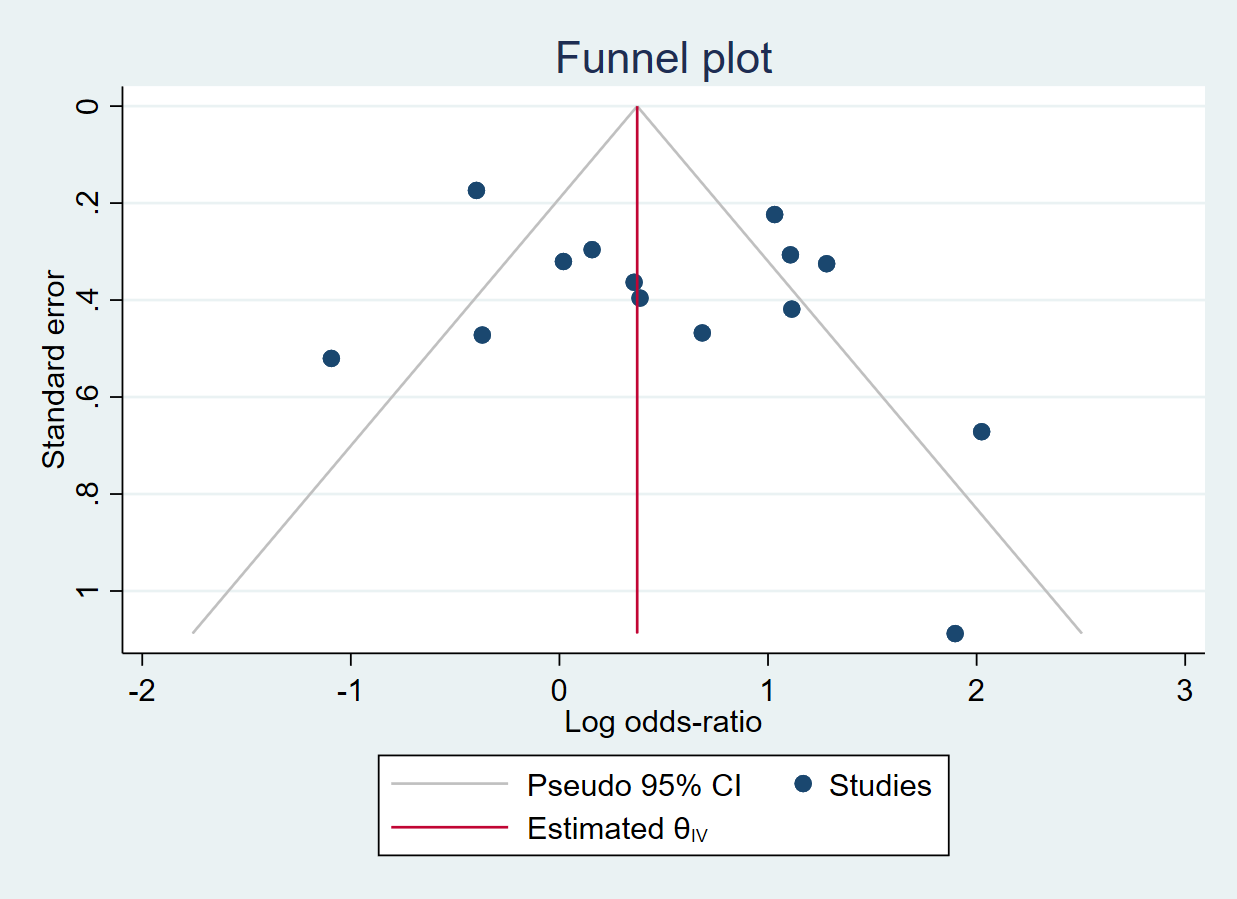


**Figure S82.** **Begg’s funnel plot of publication bias for the association between EMLO1 rs741301 polymorphisms and DN risk under the homozygote model in DN vs. DM patients**


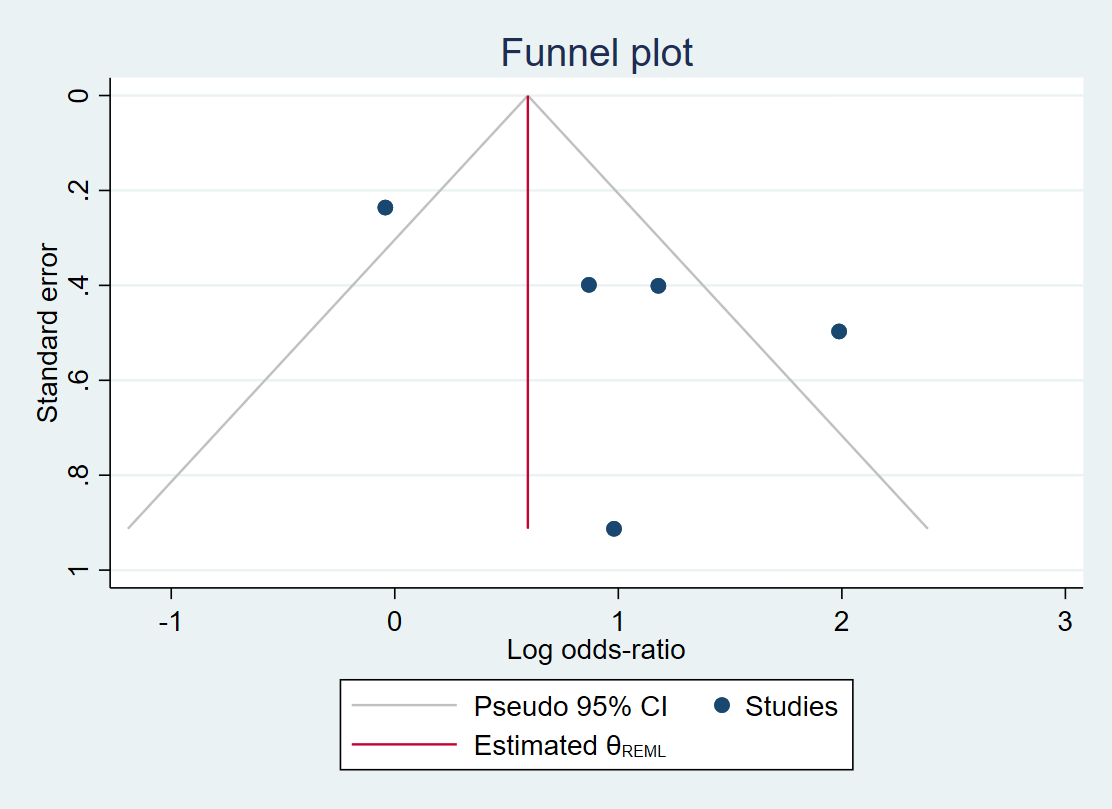


**Figure S83.** **Begg’s funnel plot of publication bias for the association between EMLO1 rs741301 polymorphisms and DN risk under the homozygote model in DN vs. healthy patients**


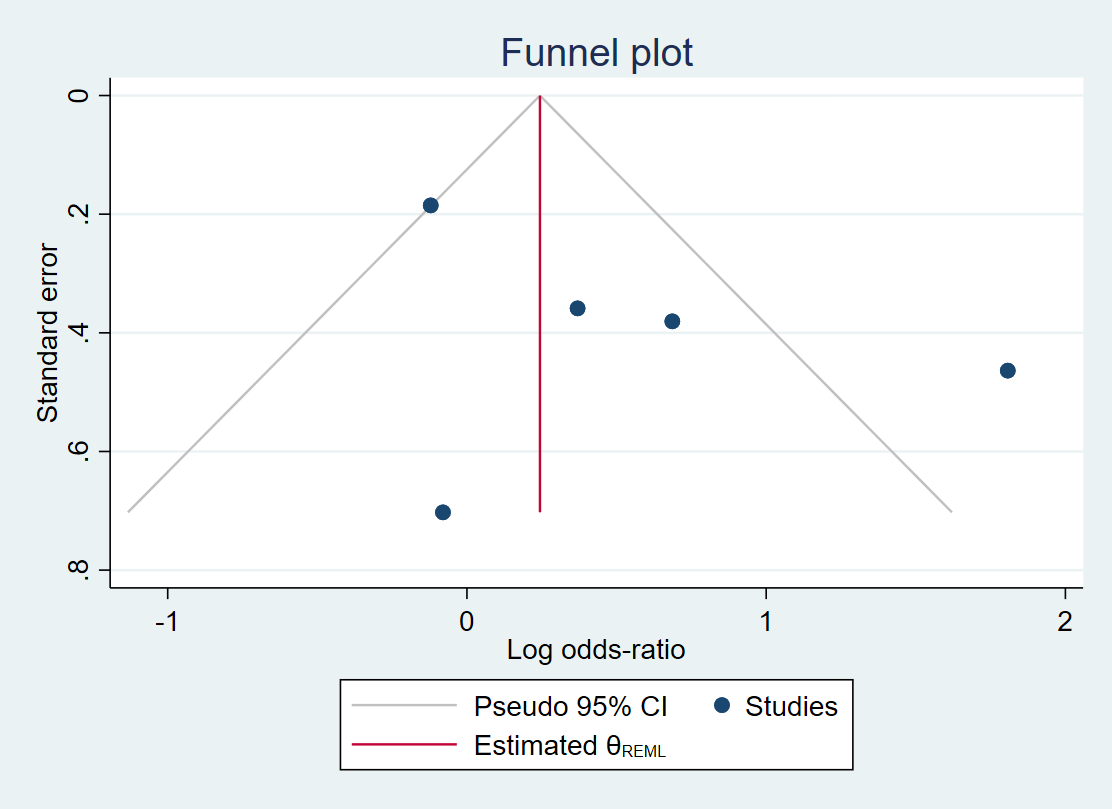


**Figure S84.** **Begg’s funnel plot of publication bias for the association between EMLO1 rs741301 polymorphisms and DN risk under the homozygote model in DM vs. healthy patients**


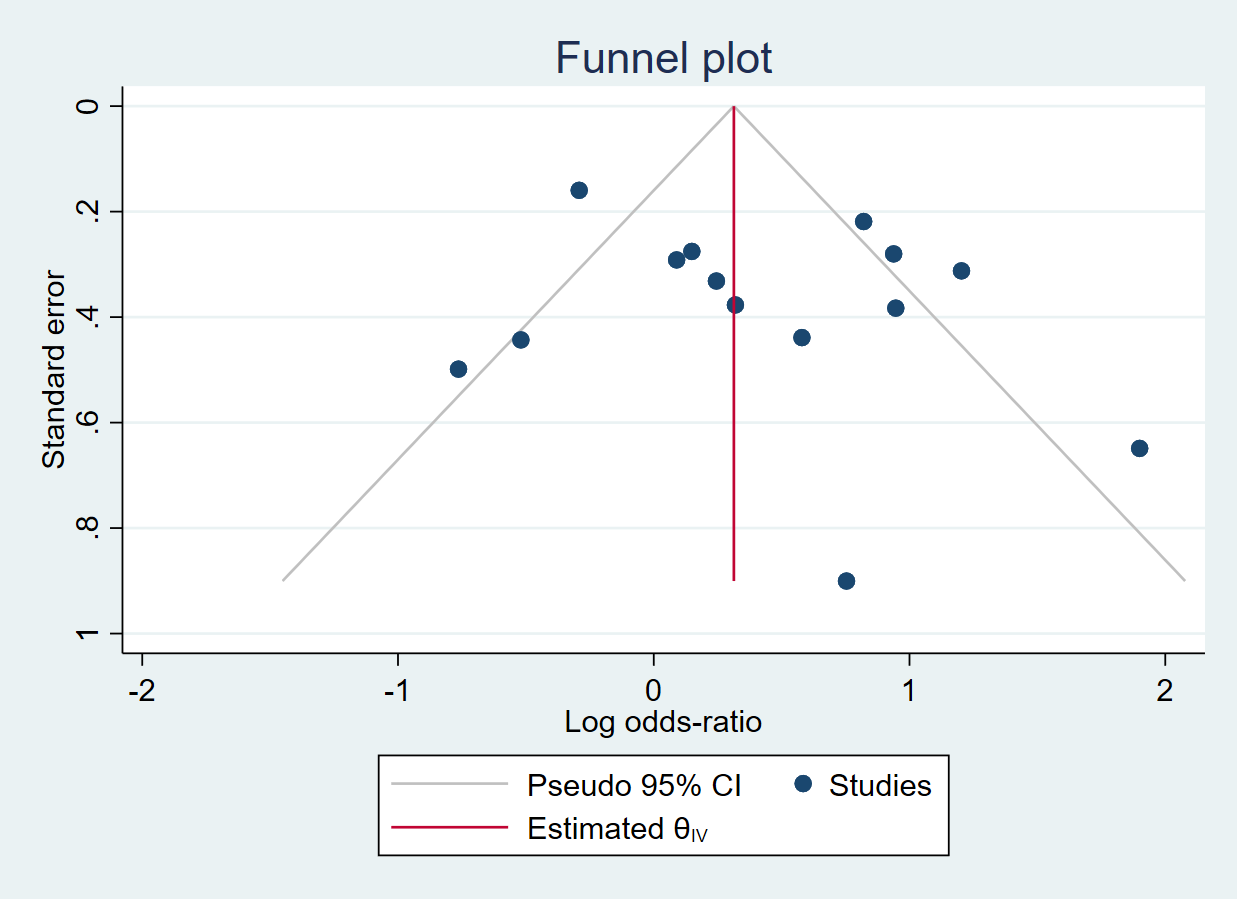


**Figure S85. Begg’s funnel plot of publication bias for the association between EMLO1 rs741301 polymorphisms and DN risk under the recessive model in DN vs. DM patients**


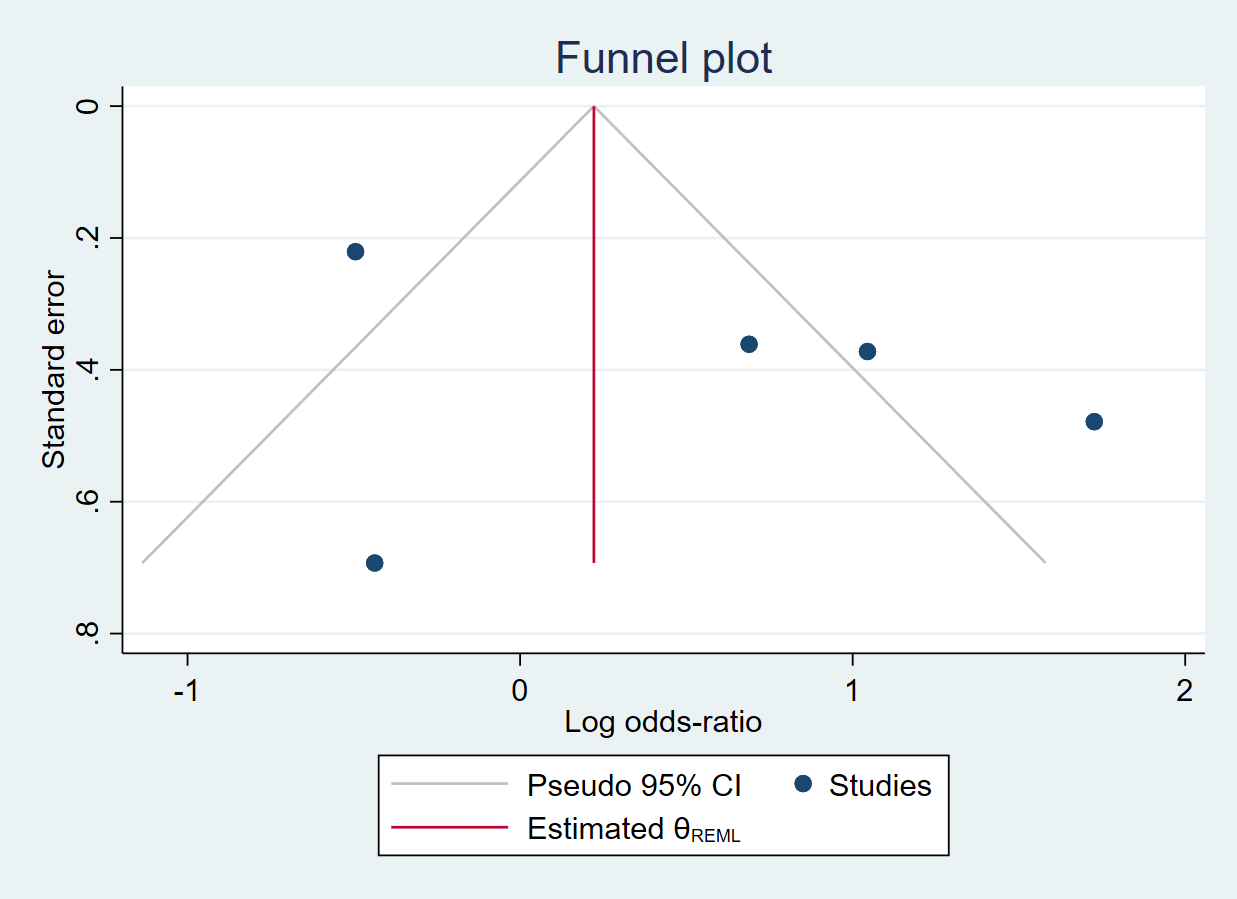


**Figure S86. Begg’s funnel plot of publication bias for the association between EMLO1 rs741301 polymorphisms and DN risk under the recessive model in DN vs. healthy patients**


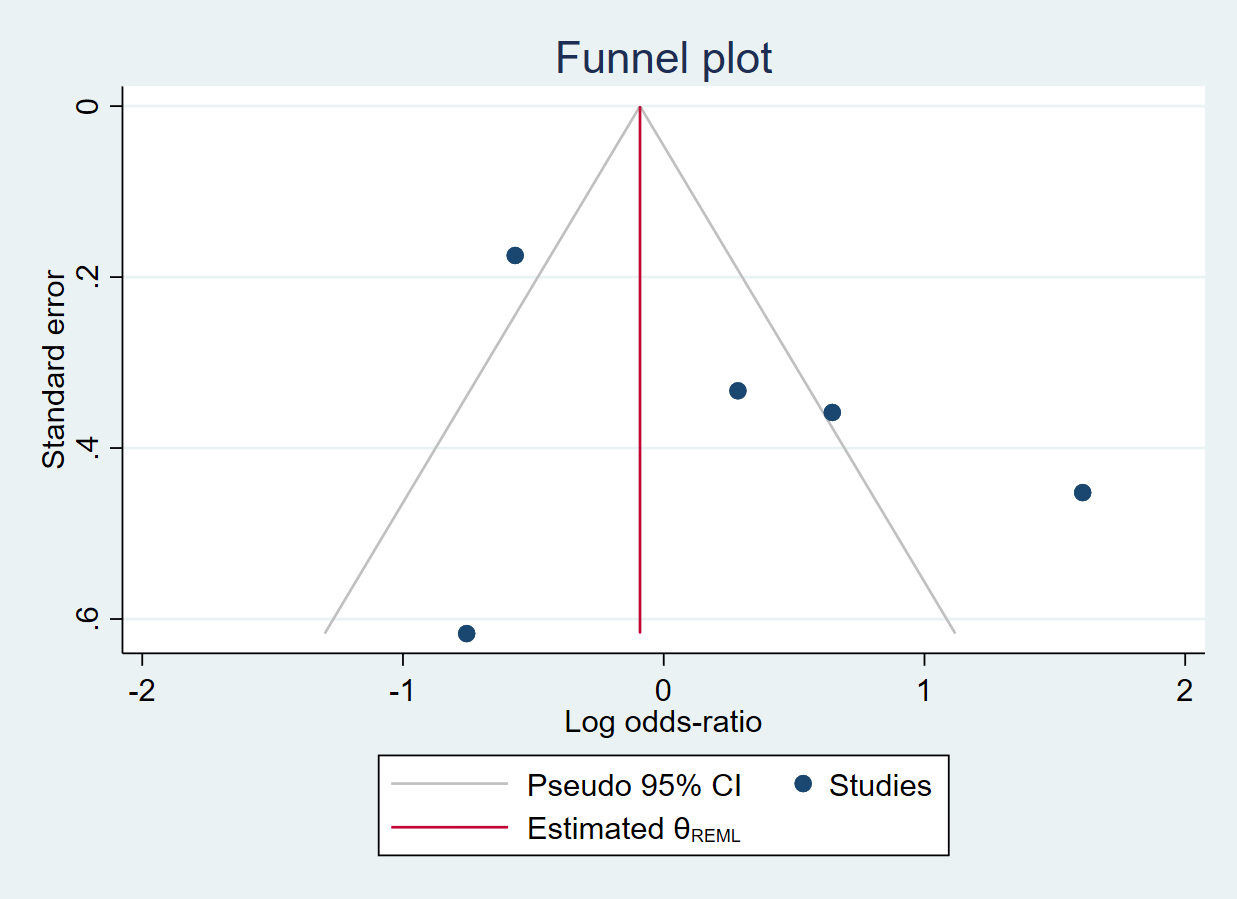


**Figure S87. Begg’s funnel plot of publication bias for the association between EMLO1 rs741301 polymorphisms and DN risk under the recessive model in DM vs. healthy patients**


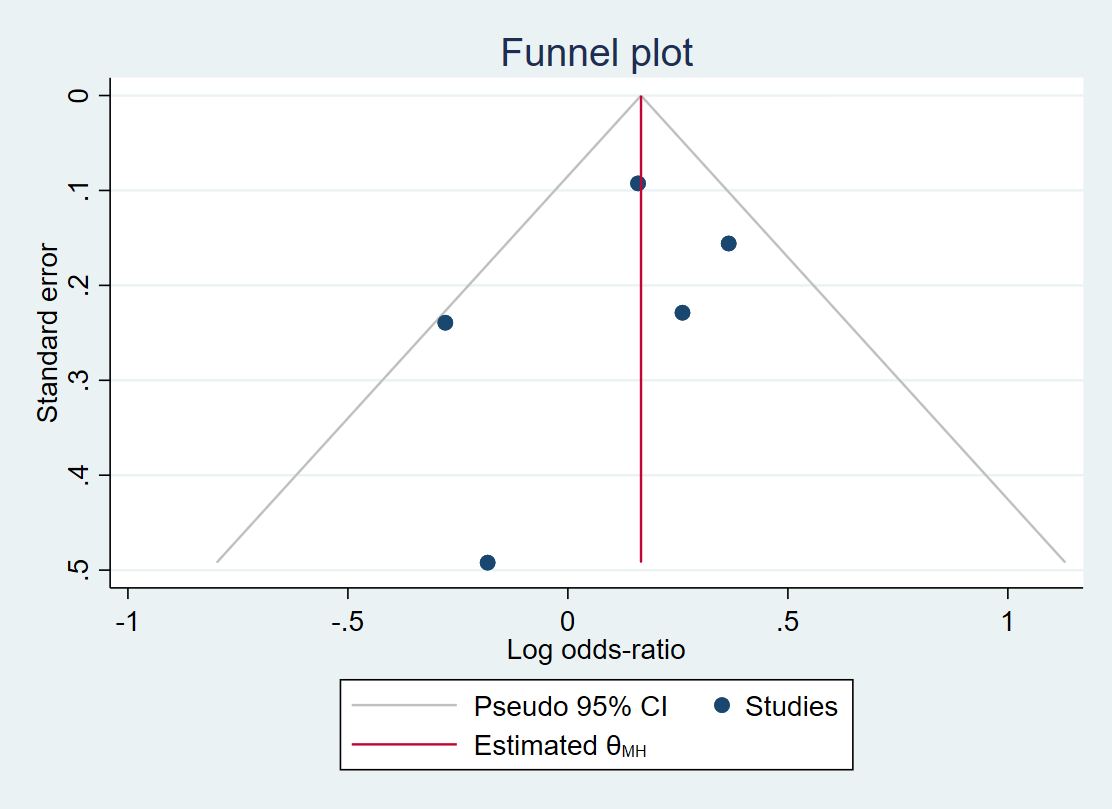


**Figure S88. Begg’s funnel plot of publication bias for the association between EMLO1 rs1345365 polymorphisms and DN risk under the allele model in DN vs. DM patients**


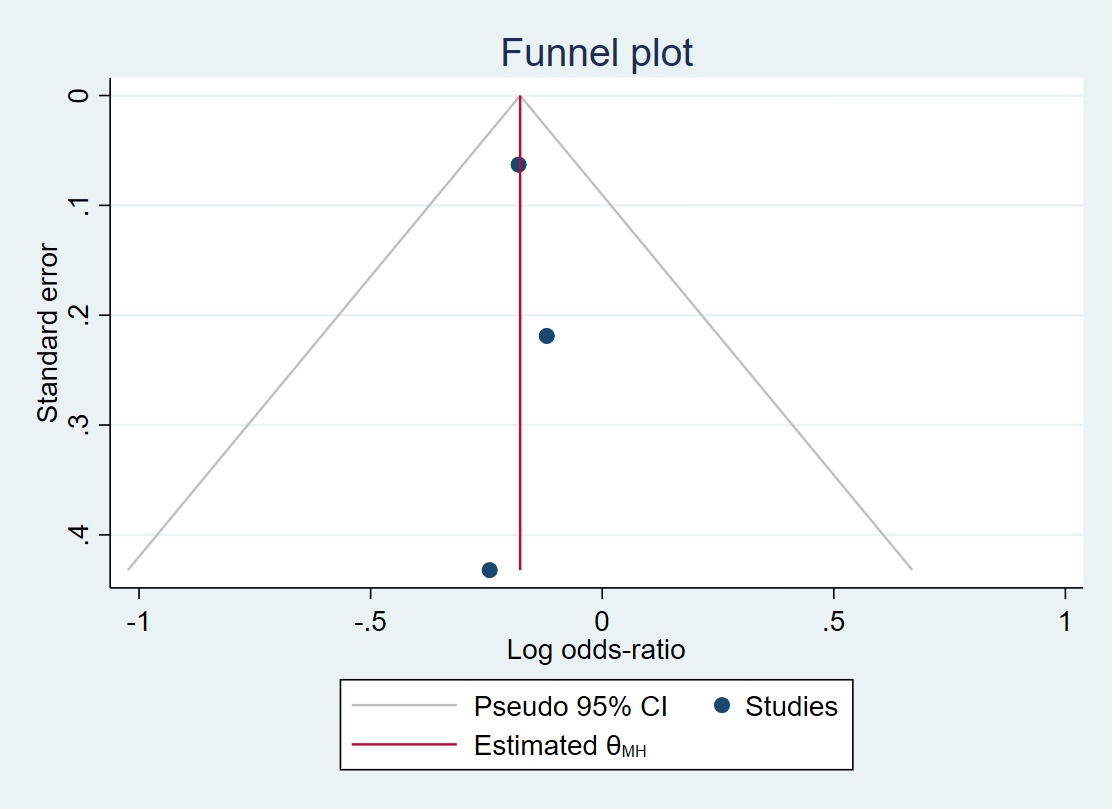


**Figure S89. Begg’s funnel plot of publication bias for the association between EMLO1 rs1345365 polymorphisms and DN risk under the allele model in DN vs. healthy patients**


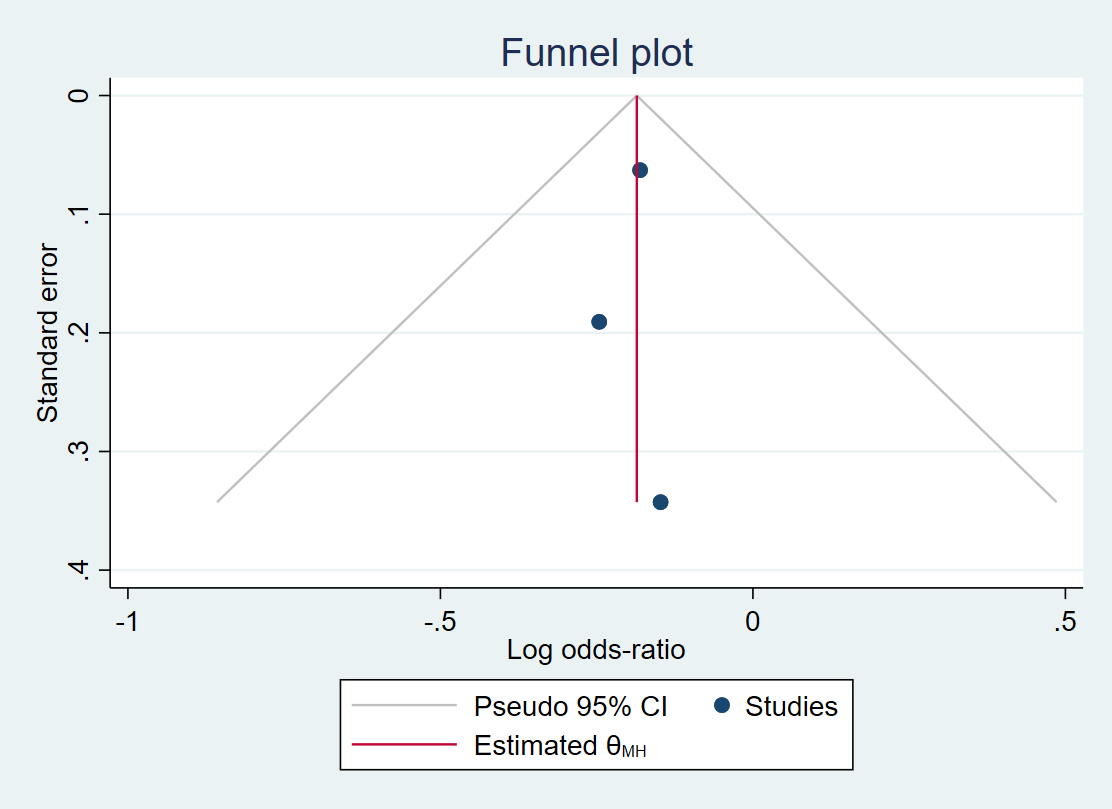


**Figure S90. Begg’s funnel plot of publication bias for the association between EMLO1 rs1345365 polymorphisms and DN risk under the allele model in DM vs. healthy patients**


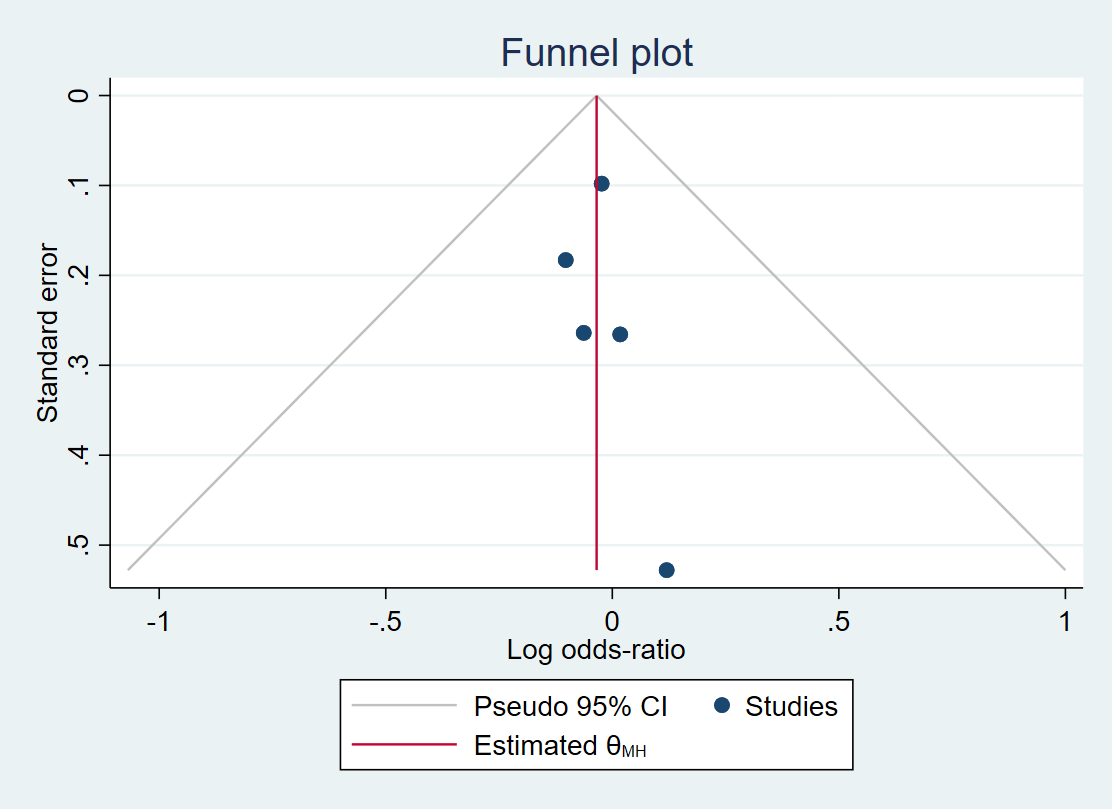


**Figure S91. Begg’s funnel plot of publication bias for the association between EMLO1 rs1345365 polymorphisms and DN risk under the dominant model in DN vs. DM patients**


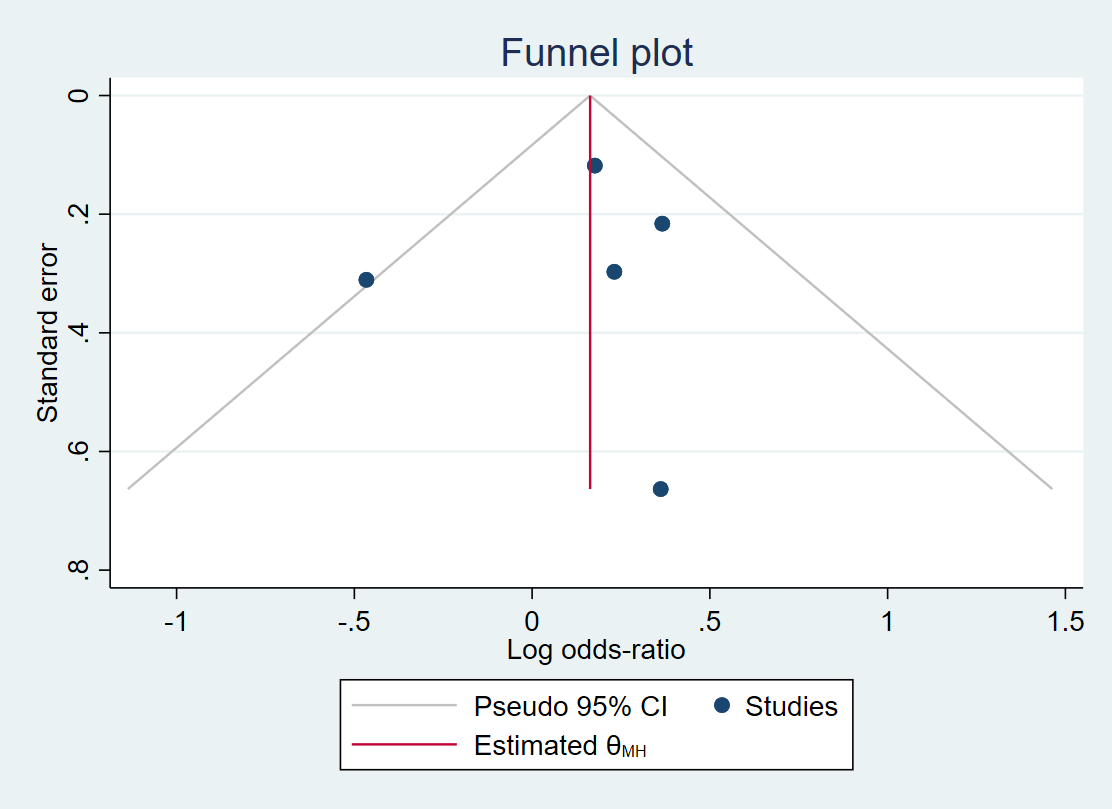


**Figure S92.** **Begg’s funnel plot of publication bias for the association between EMLO1 rs1345365 polymorphisms and DN risk under the codominant model in DN vs. DM patients**


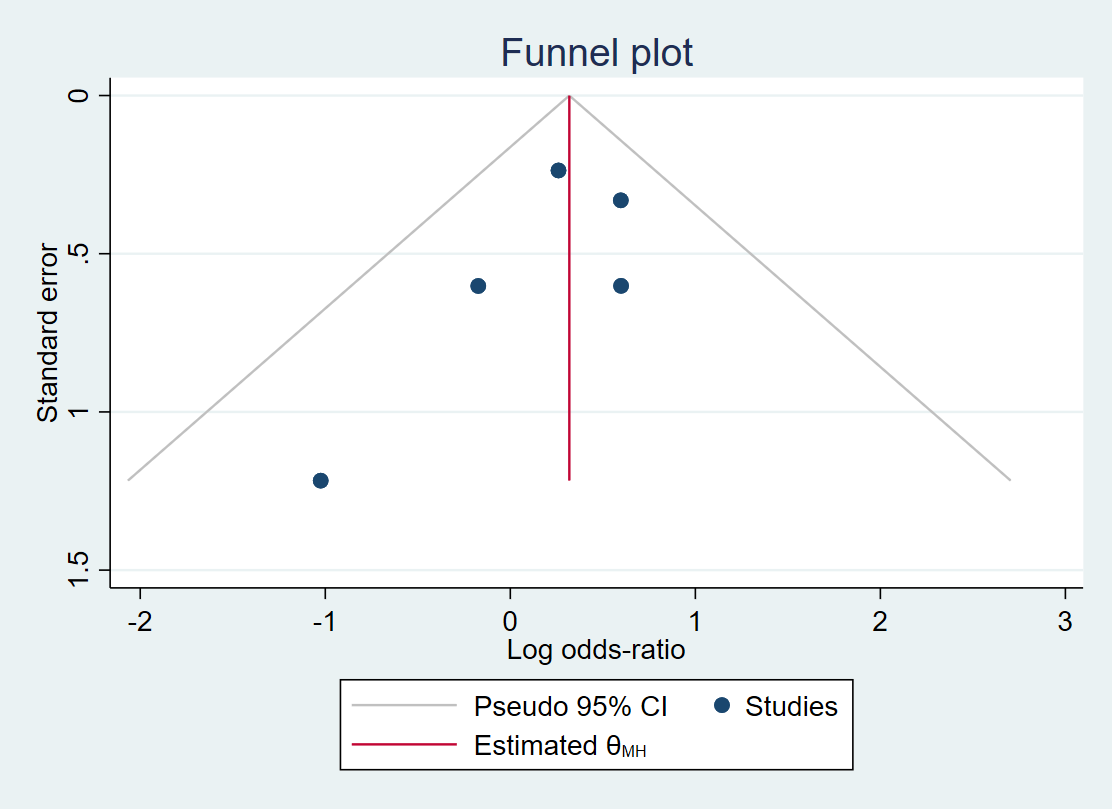


**Figure S93. Begg’s funnel plot of publication bias for the association between EMLO1 rs1345365 polymorphisms and DN risk under the homozygote model in DN vs. DM patients**

**
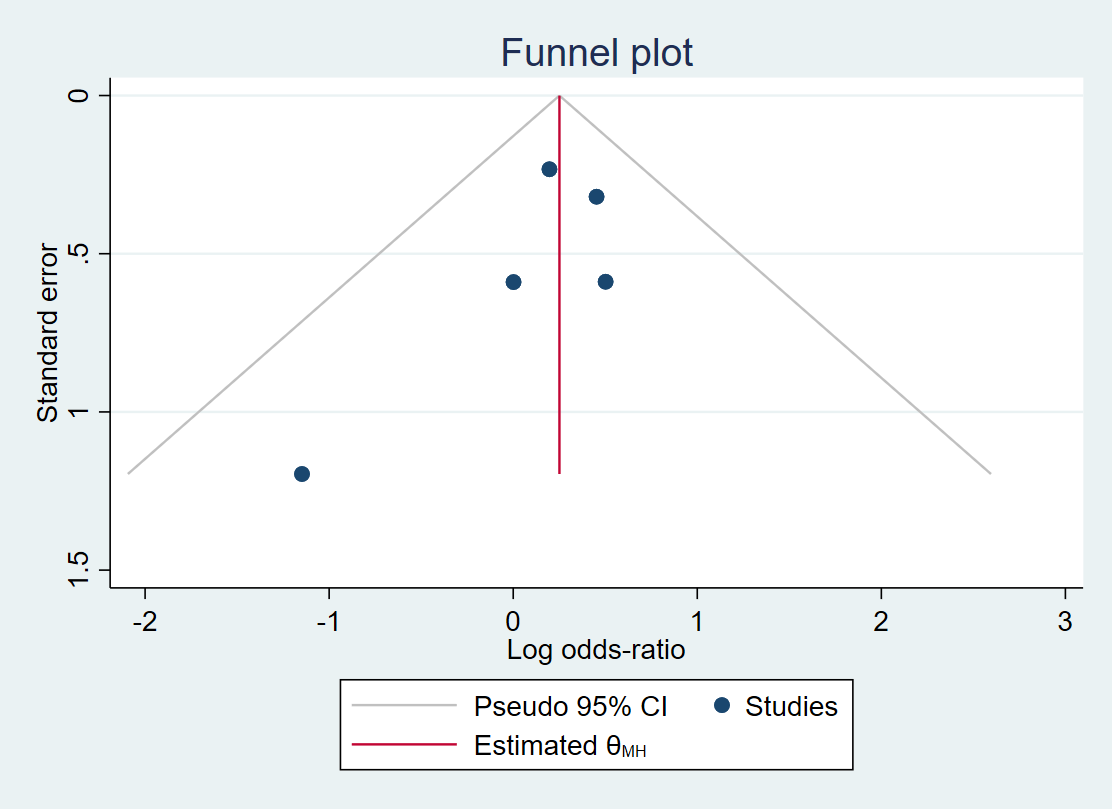
**

**Figure S94. Begg’s funnel plot of publication bias for the association between EMLO1 rs1345365 polymorphisms and DN risk under the recessive model in DN vs. DM patients**
